# Pharmacological activities of *Artemisia absinthium* and control of hepatic cancer by expression regulation of TGFβ1 and MYC genes

**DOI:** 10.1371/journal.pone.0284244

**Published:** 2023-04-13

**Authors:** Jannat Sohail, Muhammad Zubair, Khadim Hussain, Muhammad Faisal, Muhammad Ismail, Imran Haider, Rabia Mumtaz, Asif Ali Khan, Muhammad Asaf Khan

**Affiliations:** 1 Department of Bioinformatics & Biotechnology, Government College University Faisalabad, Faisalabad, Pakistan; 2 Institute of Plant Breeding and Biotechnology, MNS-University of Agriculture, Multan, Pakistan; Nigde Omer Halisdemir University, TURKEY

## Abstract

Plant derived compounds have always been an important source of medicines and have received significant attention in recent years due to their diverse pharmacological properties. Millions of plant-based herbal or traditional medicines are used to cure various types of cancers especially due to activation of proliferative genes. The aim of the present study was to characterize the altered and attenuated gene expression of the selected growth factor namely Transforming growth factor Beta -1 (TGFβ1) and MYC in human hepatoma-derived (Huh7) liver cancer cell lines in response to extracts of *Artemisia absinthium* dissolved in selected organic solvents. Ethanolic, methanolic and acetone extract of different plant parts (leaf, stem and flowers) was used to access the antiproliferative activity by MTT (3-(4,5-Dimethylthiazol-2-yl)-2,5-Diphenyltetrazolium Bromide) assay. Quantitative Real-Time RT-PCR revealed that the transcript levels of *TGFβ1* are induced in the samples treated with methanolic extract of *Artemisia absinthium*. Furthermore, reduced expression levels of MYC gene was noticed in cancerous cells suggesting antiproliferative properties of the plant. This study further highlights the resistance profile of various microbes by antimicrobial susceptibility test with plant extracts. In addition, antidiabetic effect of *Artemisia absinthium* have also shown positive results. Our study elucidates the potentials of *Artemisia absinthium* as a medicinal plant, and highlights the differential expression of genes involved in its mitogenic and anti-proliferative activity with a brief account of its pharmacological action.

## 1 Introduction

Globally liver cancer is the prominent cause of cancer associated deaths. In year 2020, according to estimates by World Health Organization, 905,677 new cases of liver cancer have been reported and out of which 830,180 people died in the world [1]. It is estimated that there will be 28.4 million cases in 2040 which is 47% more from 2020 [2].

It has been observed that liver disease remains the 5^th^ most in men and 7^th^ most basic malignant tumor in women around the world by International Agency for Research on Cancer [3]. The most common form of liver cancer is Hepatocellular carcinoma (HCC), whereas, approximately 80% cases are linked to infections caused by chronic hepatitis B virus (HBV) or hepatitis C virus (HCV) [4].

Over 38,000 cases of hepatocellular carcinoma (HCC) are estimated to occur in Latin America annually. A significant proportion of patients are still diagnosed in the later stages of the disease, although many efforts to implement effective screening programs have been reported [5]. But still a high prevalence of liver diseases in different regions is a matter of concern, reflecting the current scenario in many Western countries as well as Asian countries [6].

Recent advances examined in genomics investigation of Hepatocellular carcinoma give a logically far-reaching depiction of hepatocarcinogenesis [7]. Numerous hereditary changes that enact oncogenes or upset tumor silencer qualities are thought to go with the clearly observed histological changes. Changes in the p53 expression are a typical finding in Hepatocellular carcinoma and may assume a key job in cell multiplication [8]. Many genes like AKT-1, NFK β-1, c-MYC, STAT-3, JUN B, TGF-β1 have been involved in regulating the proliferation process in human liver cancer cells.

All around, the search for antimicrobials has found genuine serious challenge of resistance from different pathogenic microorganisms to anti-infection agents. Antimicrobial resistance represents to be a standout amongst the most concerning dangers to worldwide health and new anti-infectives are expected to conquer it [9]. Despite the fact that the expense to treat distinctive irresistible sicknesses/diseases and to discover novel antibiotic agents has been, to a great extent, expanded amid the previous decades but less success has been made [10].

The rapidly expanding occurrence of diabetes mellitus is turning into a genuine risk to humanity and its health all around. According to the World Health Organization projections, the diabetes population is likely to increase to 300 million or more by the year 2025 [11]. Also, during the last couple of years a significant part of the new bioactive medications drived from plants demonstrated anti-glycemic action with definitely more adequacy than oral hyper-glycemic arbiters utilized in clinical treatment [12]. Correlative and elective ways to deal with diabetes, for example, confinement of these phytochemicals with anti-hyperglycemic activities from restorative plants have also been practiced [11].

Restorative plants are the most critical foundation of life sparing medications for most of the total populace. Studies have been done worldwide to confirm the adequacy of plants and a portion of the discoveries have prompted the creation of various plant-based medicines for making antimicrobials and cure various diseases like hepatic cancers too [13]. Extensive works have been done on these plants to treat malignant growth and different ailments and some plant items have been advanced as anticancer medications, in light of the traditional uses and logical reports [14].

Being one of the top agrarian nation and having diverse biological areas, the therapeutic plants of Pakistan have not been investigated for their optional metabolites which are capable of treating various infections. Albeit, enormous significance of various concentrates of restorative plants from Pakistan have been accounted for their various exercises for example, antimicrobial, anti-cancer, antiviral and antioxidant, however, complete biochemical profiling of these therapeutic plants is deficient [15].

*Artemisia absinthium Linn*. (Wormwood) is a basic imperative & a shrubby plant that relates to the family of *Artemisia* with in excess of 500 species, conveyed all around in the mild territories over the globe and for the most part inside India has been reported in the Himalayan district [16]. It has an extraordinary significance as a society prescription in ancient times of Greek as an antiseptic, antimalarial, anthelmintic, antioxidant, hepatoprotective, antipyretic, and neuroprotective [17]. It has been researched that species types of *Artemisia* e.g *A*. *kulbadica*, *A*. *sieberi*, *A*. *turanica*, *A*. *santolina and A*. *diffusa* demonstrated the lethal characteristics against human Caucasian hepatocyte carcinoma (HepG-2) and human Caucasian larynx carcinoma (Hep-2) cell lines [18]. However, information about anti-cancerous properties and various phytochemicals restricting the growth of bacteria is still to be demonstrated on higher level, therefore, the aim of present study was to highlight the potential role of *A*. *absinthium* in controlling the expression of various genes involved in formation of malignant tumor and exploring the phytochemicals involved in restricting growth of selected bacteria under study, in addition, the anti-hyperglycemic role of *A*. *absinthium* has also been studied as in Pakistan there are less studies have been reported before especially on *Artemisia absinthium*.

## 2 Materials and methods

### 2.1 Extract preparation

Freshly harvested stems, leaves and flowers of *Artemisia absinthium* were collected from local market in Faisalabad (Pakistan) district. The plant was subjected to identification and authentication in the Department of Botany, Lahore College University, Lahore, Pakistan. All plant parts (stem, flower & leaves) were washed and dried at 37°C. Then these desiccated plant parts were grinded to powder. Nearly 10g of air-dried powder was taken in 100ml of aqueous, methanol, ethanol and acetone separately and placed in a shaking incubator at 37°C for overnight. Then centrifuged and filtered resulting into 5ml of liquid extract. The solvents were evaporated at 30°C by a vacuum evaporator. The extracts were then dissolved in 5ml of sterilized PBS.

### 2.2 HPLC analysis

Initially, 1g of dried leaf of *Artemisia absinthium* was extracted with 10mL of acetonitrile solution and with 0.5mM Phosphoric acid. Both mixtures were stirred for at least 2 hours at room temperature and filtered through Wattmans No. 1 filter paper and subjected to vacuum evaporator to get concentrated extracts. Residues after concentration from both extracts were dissolved into 80% methanol & 70% ethanol Solution. Extracts were filtered again through membrane of 0.45%. and filtrate was used for HPLC analysis.

The chromatographic examination was done by High Performance liquid chromatography (HPLC) utilizing diode array detection. The phenolic compounds in our extract were identified by hydro-RP 80A (250 mm × 4.60 mm, 4 μm) column. The temperature of the column was kept to ambient temperature. Among two phases, the Mobile phase was 0.1% glacial Acetic acid dissolved in distilled water (Solution A) whereas, Solution B was 0.1 glacial Acetic acid dissolved in acetonitrile solution. Injection volume was kept 4ul. The wavelength was kept 280nm to qualify the phenols. Analysis was performed under stable chromatographic conditions.

### 2.3 Antibacterial activity

#### 2.3.1 Sample collection

This retrospective analysis was conducted by examining the wound swab samples after superficial pre-cleansing of wounds with physiologic saline, each specimen was collected by rotating a sterile, pre-moistened swab, arrived at the Microbiology laboratory of Faisalabad Institute of Cardiology Hospital, Faisalabad, Pakistan from June to September 2018. Wounds from diverse aetiologies (predominantly leg wounds, diabetic foot wound and stomach and throat wounds, surgical wounds), location and duration progress (acute and chronic) were considered in this study. The wounds were sampled for microbiological analysis prior to any administration of antibiotic. In total, 200 wound samples were collected from 170 patients. Some patients had more than one wound. Then these swabs from the patients were cultured separately on different media (Blood agar, Clad Agar, MacConkey).

#### 2.3.2 Antimicrobial assay

The antimicrobial assay against Gram-positive Bacteria *Staphylococcus aureus* (*S*. *aureus*); *Gram-negative Bacilli*; *Acinetobacter* and *Klebsiella* was performed according to Kumar et al., 2011 with some modifications. Sterile 96-well microplates were used with 20 μg/mL plant extract in duplicate. The bacterial solution was prepared with turbidity comparable to the standard of 0.5 McFarland. Wells were filled with 10 μL of the bacterial culture, 20 μL of the plant extract, 50 μL of Liquid broth medium and incubated at 37°C for 24 h. PBS instead of plant extract, Liquid broth medium instead of bacterial culture were used as controls, respectively. After incubation period, bacteria growth inhibition was evaluated in a microplate reader at 640 nm. The inhibition efficiency of the particular plant extract was premeditated as percentage of bacterial growth inhibition according to the following equation:

%Inhibition=(ΔAcontrol–ΔAsample)/ΔAcontrol×100

### 2.4 Antidiabetic assay

Sterile 96-well micropipette plates were used with 12.5ul of Plant extract. Then 40ul of an enzyme d-glucosidase was added in each well. Then 140ul of 1x PBS (pH 7.4) was added in each well. These 96-well micropipette plates were then placed in incubator at 37°C for 5 min, then added 40μl of substrate (PNPG) (5mM) in each well and subjected to incubation at 37°C for 30 minutes. Na_2_CO_3_ was added to stop the reaction and optical density was measured at 405 nm. Acarbose was used as positive control (inhibitor of α-glucosidase).

### 2.5 Cytotoxicity assay

Hepatic liver cancer cell line (Huh7) was cultured in Dulbecco’s modified Eagle’s medium with, 5% inactivated fetal calf serum and supplements (serum, antibiotics) at 37°C. 190 μL of MRC-5SV2 inoculum (1.5 x 105 cells/mL) were seeded in sterile 96-well microtiter 96-well micropipette plates were incubated at 37°C and 5%CO^2^ for 72 h. Growth of cells was compared to untreated control wells of plate (100% cell growth) and control wells which contains only growth medium (0% cell growth). The viability of cells was assessed by fluorometer, 4 hours after the addition of 50 μL/well resazurin solution (2.2 μg/mL) using a microplate reader (TECAN GENios, Germany) at λex 495 nm, λem 570 nm & 650 nm. The results were expressed as % reduction in cell growth / viability compared to control wells.

### 2.6 Gene expression analysis

#### 2.6.1 RNA extraction and cDNA synthesis

For the isolation of highly pure mRNA from the cell culture line, FavorPrep Tissue Total RNA Mini Kit (Cat. No.: FATRK000) purchased from Favorgen® Biotech Corporation, Taiwan was used. The RNA extraction was performed according to manufacturer’s protocol. The cDNA was prepared from extracted RNA by using cDNA synthesis kit (Thermo Scientific RevertAid First Strand cDNA Synthesis Kit, Cat No. K1622). The cDNA was synthesized in two steps. In first step RNA was treated with DNase for the purpose of purification and then in second step cDNA was prepared according to given protocol by manufacturer.

#### 2.6.2 Real time PCR/ quantitative PCR

Quantitative PCR was performed in a CFX96 RT- PCR Detection System (Biorad), along-with Maxima^TM^ SYBR Green quantitative-PCR Master Mix (2X) from Fermentas, from USA which uses SYBR Green-I the fluorescent dye as detection system. Gene specific primers for MYC (MYC-F: 5’GAGGAGGAACAAGAAGATGAG3’ & MYC-R: 5’GTAGTTGTGCTGATGTGTGG3’) and TGFβ1 (TGFβ1-F: 5’AAGTGGACATCAACGGGTTC3’ & TGFβ1-R: 5’GTCCTTGCGGAAGTCAATGT3’. RT-PCR was performed in 20μl reactions; first cDNA was added at the bottom of PCR tube (flat cap tube) and then the reaction mixture. After the PCR has completed a melting curve between 65˚C and 95˚C withhold every 2 second was performed, allowing optimization of the acquisition temperature for each primer pair used, to avoid the formation of primer dimers.

### 2.7 Data analysis

The data were subjected to one-way and/or two-way analysis of variance (ANOVA), the means were compared using Tukey’s Honestly Significant Difference Test (HSD) using the Statistical Package for Social Sciences (SPSS) software version 26.0 Armonk, NY, USA. Optimization of the parameters was conducted using response surface methodology (RSM) via the Box–Behnken design (BBD) on Design-Expert^®^ software version 12.0 (Design-Expert version 12 Stat-Ease Inc. Suite 6400, Minneapolis, MN 55413, USA).

## 3 Results

### 3.1 Antimicrobial activity of *Artemisia absinthium*

The antimicrobial activity of different plant parts of *Artemisia absinthium was* tested against *Staphylococcus aureus*, *Acinetobacter*, *Klebsiella* and *Gram-negative bacilli* by 96 well micro plate method [19].

#### 3.1.1 Antimicrobial activity of leaf

**Fig 1:**
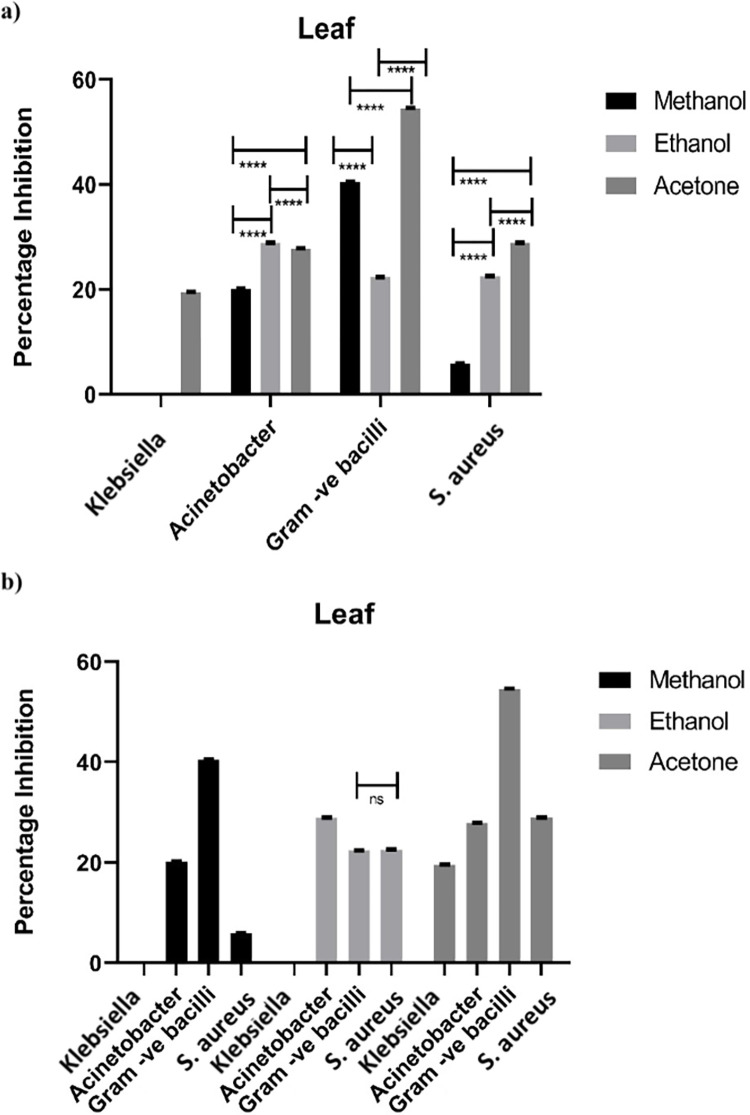
Statistical comparison of antimicrobial activity of leaf extracts against various bacteria. (a) Effect of all three solvents on an individual bacterium. (b) Comparison of each solvent with all selected bacterial spp. Bar represents ± SEM. Data was analyzed by two-way analysis of variance (ANOVA) (P<0.001). * = significant; ns = non-significant.

Antibacterial/antimicrobial activity of leaf of *Artemisia absinthium* was checked on nutrient broth media. (Fig 1). Three different kinds of solvents i.e., Methanol, Ethanol & Acetone of leaf extract was used to evaluate the antimicrobial activity. Each plant extracts have exhibited antimicrobial potential (i.e., percentage inhibition) ranging from 5.9% to 55%. All three extracts showed potent/positive results for *S*. *aureus*, and resulted into 28.8% of antimicrobial activity with acetone extract, 22.4% with ethanol and least with methanol as 5.8%. However, highest rate of inhibition was obtained with acetone extract for *Gram negative bacilli* as 57.7%, 40.5% with methanol and 22.3% with ethanol (Fig 1A). In case of *Acinetobacter*, all three solvents showed significantly different inhibition properties (ranging from 20% to 29) from each other. Ethanol was at the top with inhibition of 29% and methanol was least with 20%. Moreover, microbial growth of *Klebsiella* spp. was also impeded by extract of acetone with inhibition rate of 19.5%. So, it was clear that all the solvent significantly interdicted the growth rate of all the bacterial spp. on an individual level with varying intensity (Fig 1B). However, non-significant difference has been observed between ethanolic and acetone extract of leaf on *Gram negative bacilli* and *S*. *aureus*. Furthermore, highest level of percentage inhibition was observed with acetone and methanolic extract with *Gram negative bacilli*.

#### 3.1.2 Antimicrobial activity of flower

**Fig 2:**
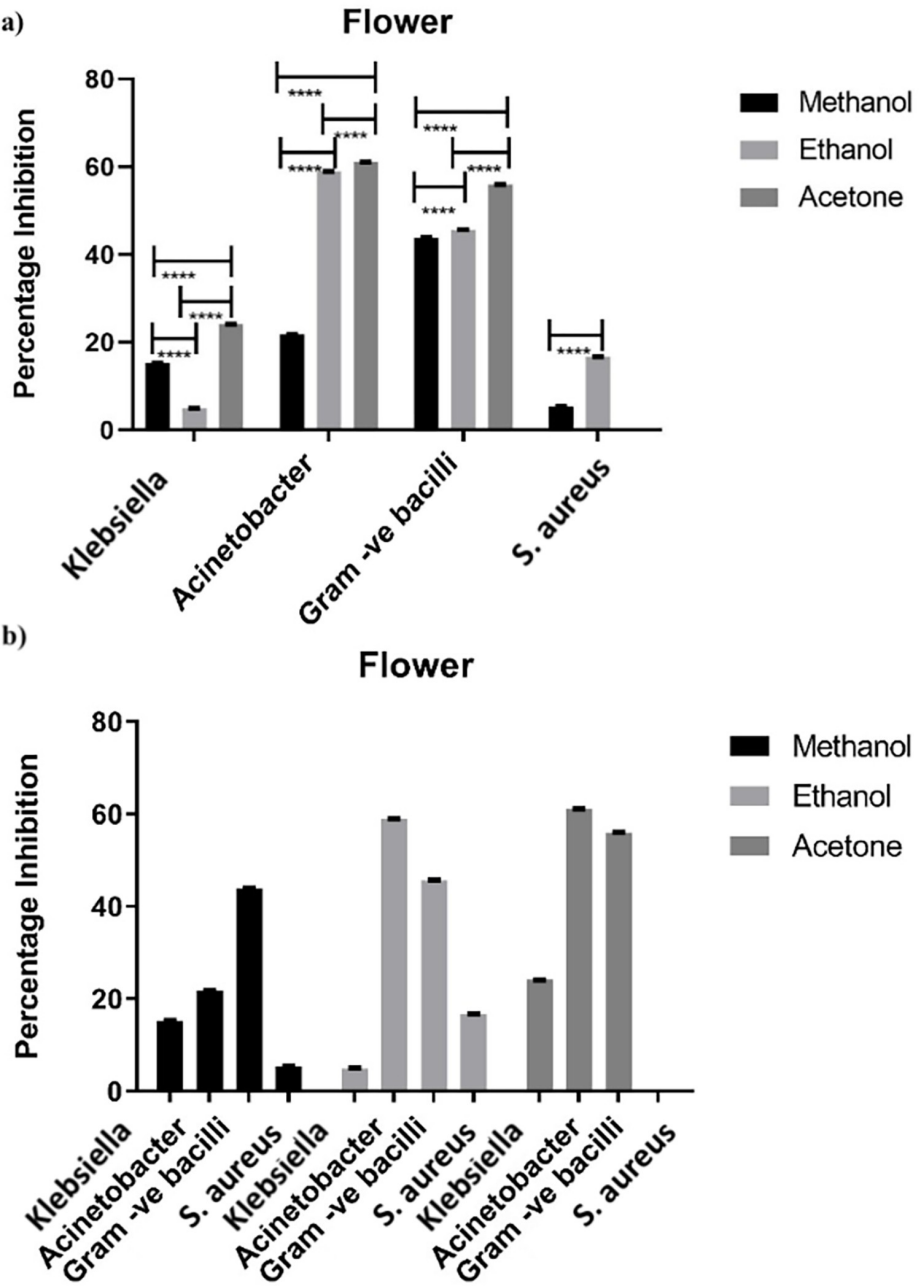
Statistical comparison of antimicrobial activity of flower against various bacteria. (a) Effect of each solvent on individual bacterium. (b) Comparison of each solvent with all selected bacterial spp. Bar represents ± SEM. Data was analyzed by two-way analysis of variance (ANOVA) (P<0.001). * = significant, ns = non-significant.

Flowers of *Artemisia absinthium* were also used to assess their antibacterial activity on nutrient broth media against the same selected bacterial species (Fig 2). Extracts of flower were dissolved in three organic solvents. Significant results have been found in all plant extracts having antimicrobial potential ranging from 5% to 62% (Fig 2A). However, all three extracts have proven to be superlative in case of *Gram-negative bacilli*, giving one of the towering rates of inhibition as 55.6% with acetone, 45.6% with ethanol and 43.8% with methanol. On the other hand, among all tested bacterial spp. acetone extract displayed highest rate of inhibition capacity as 61% against *Acinetobacter* followed by ethanol with 58.9%. While methanol extracts showed the least rate of inhibition of 21.7%.

Growth of *Klebsiella* was also hindered by all three extracts ranging from 5.1% to 24.1% at remarkable rate. Extracts of flowers were also interestingly good in the case of *Staphylococcus aureus*, as extract with each solvent obstructed its growth at quite minimal rate i.e., acetone as 16.6% and methanol as 5.3%. Comparison of effect of each solvent clearly depicts that peerless results by acetone and ethanol extract have been found against *Gram negative bacilli & Acinetobacter* (Fig 2B). However, methanol extract has played as pre-eminent inhibitor of each selected bacterial spp. under study (Fig 2B).

#### 3.1.3 Antimicrobial activity of stem

**Fig 3:**
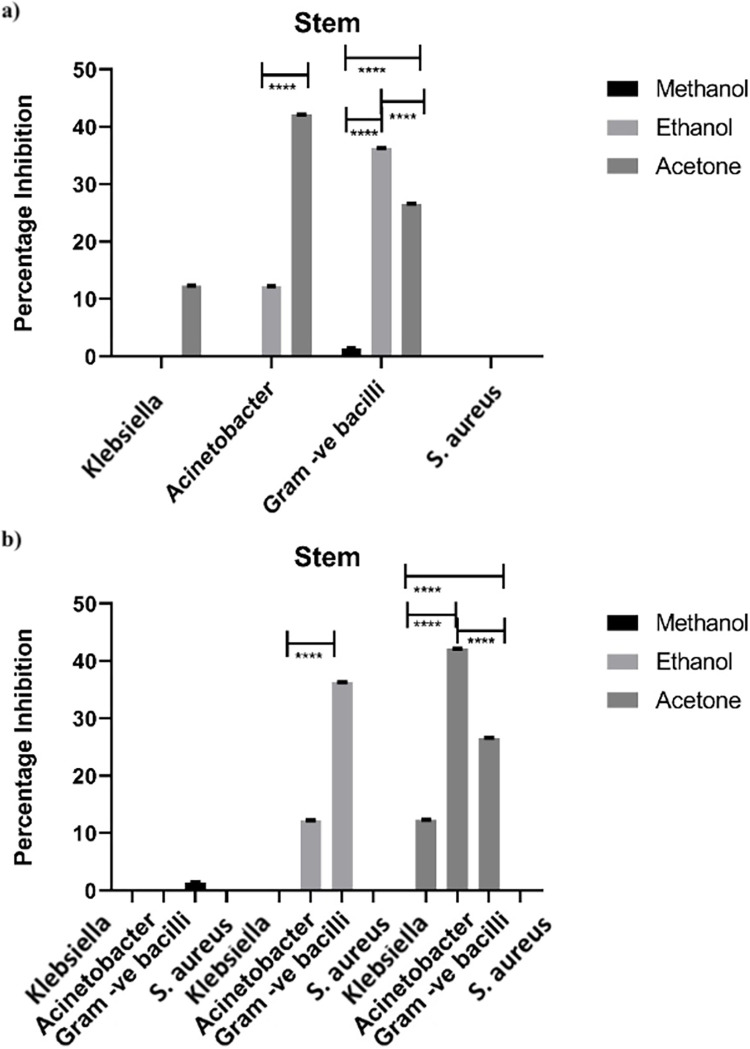
Statistical comparison of antimicrobial activity of stem against various bacteria (a) Effect of each solvent on individual bacterium. (b) Comparison of each solvent with all for selected bacterial spp. Bar represents ± SEM. Data was analyzed by two-way analysis of variance (ANOVA) (P<0.001). * = significant, ns = non-significant.

Antimicrobial activity of stem of *Artemisia absinthium* was also tested following the results of leaf and flower (Fig 3). Results manifested a noteworthy inhibitory activity of all samples with selected bacterial spp. (Fig 3A).

Adequate rate of inhibition was given by acetone as 36.2% and ethanol 26.5% against *Gram-negative bacilli* respectively. Unexpectedly, highest rate of growth hindrance w.r.t to other two solvents was 42.1% with acetone and 12.6% with ethanol in case of *Acinetobacter*. Extracts of methanol and ethanol were less efficient in obstructing the growth of bacteria in case of *Klebsiella* and *Staphylococcus aureus*. But only 12.3% interdiction was obtained with extract of acetone with *Klebsiella*.

Unexpectedly quite good results among all solvents were drawn by acetone and also somehow with ethanol extract as shown by graphs in Fig 3. It is interesting that the maximum intensity of inhibition was against *Gram negative bacilli & Acinetobacter* by acetone and ethanol, although it was least in case of methanol.

#### 3.1.4 Optimization of *Artemesia absenthia* and its anti-microbial activity from various plant parts via response surface methodology

For all the plant parts of *A*. *absenthia* which came under investigation was assessed based on response surface methodology via Box-Behnken design (Table 1). The following quadratic response surface model was fitted to the data.

$$Y=\beta_{o}+\sum_{i=1}^{k}\beta_{i}F_{i}+\sum_{i=1}^{k}\beta_{\mathrm{ii}}F_{i}^{2}+\sum \sum_{i<j=1}^{k}\beta_{\mathrm{ij}}F_{i}F_{j}+\varepsilon$$

where “*Y”* was the antimicrobial activity, “*β*_0_” was the intercept constant, “*βi”*, “*βii”*, “*βij”* was the regression coefficients. “*F*_*i*_*”*, “*F*_*j*_*”* was coded values of microbes while “ε" is error term.

**Table 1:** A design approach for determining the optimization of anti-microbial activity of *A*. *absenthia* via Box-Behnken design.

Microbes	Coded Symbol	Range																	
		Leaves						Flowers						Stem					
		Methanol		Ethanol		Acetone		Methanol		Ethanol		Acetone		Methanol		Ethanol		Acetone	
		+1	–1	+1	–1	+1	–1	+1	–1	+1	–1	+1	–1	+1	–1	+1	–1	+1	–1
*Klebsiella*	A	0	0.1	0	0.1	9	29	5	25	2	6	14	34	0	0.1	0	0.1	2	22
*Acinetobacter*	B	10	30	18	38	17	37	11	31	48	68	51	71	0	0.1	2	22	32	52
Gram–ve *Bacilli*	C	30	50	12	32	44	64	33	53	35	55	45	65	1	2	26	46	16	36
*S*. *aureus*	D	1	10	12	32	18	38	1	10	6	26	0	0.1	0	0.1	0	0.1	0	0.1

*3*.*1*.*4*.*1 Leaves*. ***3*.*1*.*4*.*1*.*1 Antimicrobial activity in A*. *absenthia leaves in response to methanol*.** For the optimization of anti-microbial activity, analysis of variance was performed via quadratic model and response to methanol and leaves was checked in *A*. *absenthia*. The following regression equations was obtained.

Inhibition (Leaf-Methanol) = 0.772–0.0058 A + 0.191 B– 0.117 C + 0.085 D– 0.0006 AB + 0.0018 AC– 0.00024 AD –0.011 BC– 0.0268 BD– 0.0045 CD + 0.00034 A^2^– 0.0293 B^2^ + 0.0179 C^2^ –0.0059 D^2^

According to this regression equation, leaf showed positive effect in treatment with methanol as well as the anti-microbial activity was found to be significant. According to analysis of variance, the model fitted was found to be significant as the P-value was less than 0.05. All the plant parts found significant in antimicrobial properties (P<0.05). Based on the analysis of variance applied to this model, it became obvious that this model has shown significant response at 5% level of significance while this model is also very suitable and reproducible due to having very little lack of fit (P>0.05). The coefficient of determination (R^2^) also confirms that with 99% surety this data is highly significant and can be applied under various conditions. Thus, the optimum parameters have also been defined as shown in S1 Table. The surface plots developed based upon this analysis also depicts that *A*. *absenthia* have substantial anti-microbial ability which can be good alternate to commonly available anti-microbial substances.

***3*.*1*.*4*.*1*.*2 Antimicrobial activity in A*. *absenthia leaves in response to ethanol*.** For the optimization of anti-microbial activity, analysis of variance was performed via quadratic model and response to ethanol and leaves was checked in *A*. *absenthia*. The following regression equations was obtained.

Inhibition (Leaf-Ethanol) = 1.217–0.004 A—0.0598 B + 0.0681 C + 0.0681 D + 0.0009 AB + 0.0003 AC + 0.0003 AD + 0.0676 BC + 0.0676 BD + 0.0012 CD + 0.0042 A^2^ - 0.058 B^2^ - 0.0614 C^2^ -0.0614 D^2^

According to this regression equation, leaf showed positive effect in treatment with ethanol as well as the anti-microbial activity was found to be significant. According to analysis of variance, the model fitted was found to be significant as the P-value was less than 0.05. All the plant parts found significant in antimicrobial properties (P<0.05). Based on the analysis of variance applied to this model, it became obvious that this model has shown significant response at 5% level of significance while this model is also very suitable and reproducible due to having very little lack of fit (P>0.05). The coefficient of determination (R^2^) also confirms that with 91% surety this data is highly significant and can be applied under various conditions. Thus, the optimum parameters have also been defined as shown in S2 Table. The surface plots developed based upon this analysis also depicts that *A*. *absenthia* have substantial anti-microbial ability which can be good alternate to commonly available anti-microbial substances.

***3*.*1*.*4*.*1*.*3 Antimicrobial activity in A*. *absenthia leaves in response to acetone*.** For the optimization of anti-microbial activity, analysis of variance was performed via quadratic model and response to acetone and leaves was checked in *A*. *absenthia*. The following regression equations was obtained.

Inhibition (Leaf-Acetone) = 0.859–0.029 A + 0.065 B—0.068 C + 0.073 D—0.045 AB + 0.0096 AC—0.037 AD—0.0003 BC—0.016 BD—0.001 CD + 0.0357 A^2^ + 0.010 B^2^ - 0.001 C^2^ + 0.0056 D^2^

According to this regression equation, leaf showed positive effect in treatment with acetone as well as the anti-microbial activity was found to be significant. According to analysis of variance, the model fitted was found to be significant as the P-value was less than 0.05. All the plant parts found significant in antimicrobial properties (P<0.05). Based on the analysis of variance applied to this model, it became obvious that this model has shown significant response at 5% level of significance while this model is also very suitable and reproducible due to having very little lack of fit (P>0.05). The coefficient of determination (R^2^) also confirms that with 95% surety this data is highly significant and can be applied under various conditions. Thus, the optimum parameters have also been defined as shown in S3 Table. The surface plots developed based upon this analysis also depicts that *A*. *absenthia* have substantial anti-microbial ability which can be good alternate to commonly available anti-microbial substances.

*3*.*1*.*4*.*2 Flower*. ***3*.*1*.*4*.*2*.*1 Antimicrobial activity in A*. *absenthia flowers in response to methanol*.** For the optimization of anti-microbial activity, analysis of variance was performed via quadratic model and response to acetone and leaves was checked in *A*. *absenthia*. The following regression equation was obtained.

Inhibition (Flower-Methanol) = 0.852 + 0.124 A + 0.139 B—0.101 C—0.0348 D—0.033 AB—0.0034 AC—0.033 AD—0.0043 BC -0.0021 BD + 0.011 CD—0.0018 A^2^ - 0.021 B^2^ + 0.0081 C^2^ + 0.011 D^2^

According to this regression equation, leaf showed positive effect in treatment with acetone as well as the anti-microbial activity was found to be significant. According to analysis of variance, the model fitted was found to be significant as the P-value was less than 0.05. All the plant parts found significant in antimicrobial properties (P<0.05). Based on the analysis of variance applied to this model, it became obvious that this model has shown significant response at 5% level of significance while this model is also very suitable and reproducible due to having very little lack of fit (P>0.05). The coefficient of determination (R^2^) also confirms that with 98% surety this data is highly significant and can be applied under various conditions. Thus, the optimum parameters have also been defined as shown in S4 Table. The surface plots developed based upon this analysis also depicts that *A*. *absenthia* have substantial anti-microbial ability which can be good alternate to commonly available anti-microbial substances.

***3*.*1*.*4*.*2*.*2 Antimicrobial activity in A*. *absenthia flowers in response to ethanol*.** For the optimization of anti-microbial activity, analysis of variance was performed via quadratic model and response to ethanol and flowers was checked in *A*. *absenthia*. The following regression equations was obtained.

Inhibition (Flower-Ethanol) = 0.991–0.016 A -0.071 B + 0.073 C + 0.082 D + 0.0026 AB—3.880 AC—3.880 AD + 0.0283 BC—0.0001 BD—0.0136 CD + 0.005 A^2^ - 0.0028 B^2^ -0.0162 C^2^ - 0.002 D^2^

According to this regression equation, flower showed positive effect in treatment with ethanol as well as the anti-microbial activity was found to be significant. According to analysis of variance, the model fitted was found to be significant as the P-value was less than 0.05. All the plant parts found significant in antimicrobial properties (P<0.05). Based on the analysis of variance applied to this model, it became obvious that this model has shown significant response at 5% level of significance while this model is also very suitable and reproducible due to having very little lack of fit (P>0.05). The coefficient of determination (R^2^) also confirms that with 97% surety this data is highly significant and can be applied under various conditions. Thus, the optimum parameters have also been defined as shown in S5 Table. The surface plots developed based upon this analysis also depicts that *A*. *absenthia* have substantial anti-microbial ability which can be good alternate to commonly available anti-microbial substances.

***3*.*1*.*4*.*2*.*3 Antimicrobial activity in A*. *absenthia flowers in response to acetone*.** For the optimization of anti-microbial activity, analysis of variance was performed via quadratic model and response to acetone and flowers was checked in *A*. *absenthia*. The following regression equations was obtained.

Inhibition (Flower-Acetone) = 1.128 + 0.064 A—0.043 B + 0.028 C—0.0003 D—0.001 AB—0.0072 AC + 6.339 AD + 0.0513 BC + 4.628 BD + 1.522 CD—0.0058 A^2^ - 0.0225 B^2^ - 0.0287 C^2^ -0.001 D^2^

According to this regression equation, flower showed positive effect in treatment with acetone as well as the anti-microbial activity was found to be significant. According to analysis of variance, the model fitted was found to be significant as the P-value was less than 0.05. All the plant parts found significant in antimicrobial properties (P<0.05). Based on the analysis of variance applied to this model, it became obvious that this model has shown significant response at 5% level of significance while this model is also very suitable and reproducible due to having very little lack of fit (P>0.05). The coefficient of determination (R^2^) also confirms that with 97% surety this data is highly significant and can be applied under various conditions. Thus, the optimum parameters have also been defined as shown in S6 Table. The surface plots developed based upon this analysis also depicts that *A*. *absenthia* have substantial anti-microbial ability which can be good alternate to commonly available anti-microbial substances.

*3*.*1*.*4*.*3 Stem*. ***3*.*1*.*4*.*3*.*1 Antimicrobial activity in A*. *absenthia stem in response to methanol*.** For the optimization of anti-microbial activity, analysis of variance was performed via quadratic model and response to methanol and stem was checked in *A*. *absenthia*. The following regression equations was obtained.

Inhibition (Stem-Methanol) = 0.129 + 0.040 A + 0.040 B—0.176 C + 0.040 D—0.0189 AB—0.0161 AC -0.0189 AD—0.0161 BC—0.0189 BD—0.0161 CD + 0.0227 A^2^ + 0.0227 B^2^ + 0.122 C^2^ + 0.0227 D^2^

According to this regression equation, stem showed positive effect in treatment with methanol as well as the anti-microbial activity was found to be significant. According to analysis of variance, the model fitted was found to be significant as the P-value was less than 0.05. Stem found significant in antimicrobial properties (P<0.05). Based on the analysis of variance applied to this model, it became obvious that this model has shown significant response at 5% level of significance while this model is also very suitable and reproducible due to having very little lack of fit (P>0.05). The coefficient of determination (R^2^) also confirms that with 99% surety this data is highly significant and can be applied under various conditions. Thus, the optimum parameters have also been defined as shown in S7 Table. The surface plots developed based upon this analysis also depicts that *A*. *absenthia* have substantial anti-microbial ability which can be good alternate to commonly available anti-microbial substances.

***3*.*1*.*4*.*3*.*2 Antimicrobial activity in A*. *absenthia stem in response to ethanol*.** For the optimization of anti-microbial activity, analysis of variance was performed via quadratic model and response to ethanol and stem was checked in *A*. *absenthia*. The following regression equations was obtained.

Inhibition (Stem-Ethanol) = 0.501 + 0.0005 A + 0.328 B—0.100 C + 0.0005 D—0.00048 AB + 1.118 AC -0.001 AD—0.052 BC—0.00048 BD + 1.118 CD + 0.0018 A^2^ - 0.0698 B^2^ + 0.0204 C^2^ + 0.0018 D^2^

According to this regression equation, stem showed positive effect in treatment with ethanol as well as the anti-microbial activity was found to be significant. According to analysis of variance, the model fitted was found to be significant as the P-value was less than 0.05. Stem found significant in antimicrobial properties (P<0.05). Based on the analysis of variance applied to this model, it became obvious that this model has shown significant response at 5% level of significance while this model is also very suitable and reproducible due to having very little lack of fit (P>0.05). The coefficient of determination (R^2^) also confirms that with 99% surety this data is highly significant and can be applied under various conditions. Thus, the optimum parameters have also been defined as shown in S8 Table. The surface plots developed based upon this analysis also depicts that *A*. *absenthia* have substantial anti-microbial ability which can be good alternate to commonly available anti-microbial substances.

***3*.*1*.*4*.*3*.*3 Antimicrobial activity in A*. *absenthia stem in response to acetone*.** For the optimization of anti-microbial activity, analysis of variance was performed via quadratic model and response to acetone and stem was checked in *A*. *absenthia*. The following regression equations was obtained.

Inhibition = 0.949 + 0.134 A—0.111 B + 0.126 C—0.0005 D -0.0016 AB—0.0349 AC -1.066 AD + 0.0233 BC + 0.00015 BD—1.066 CD—0.0131 A^2^ + 0.0069 B^2^- 0.025 C^2^ + 0.0044 D^2^.

According to this regression equation, stem showed positive effect in treatment with acetone as well as the anti-microbial activity was found to be significant. According to analysis of variance, the model fitted was found to be significant as the P-value was less than 0.05. Stem found significant in antimicrobial properties (P<0.05). Based on the analysis of variance applied to this model, it became obvious that this model has shown significant response at 5% level of significance while this model is also very suitable and reproducible due to having very little lack of fit (P>0.05). The coefficient of determination (R^2^) also confirms that with 99% surety this data is highly significant and can be applied under various conditions. Thus, the optimum parameters have also been defined as shown in S9 Table. The surface plots developed based upon this analysis also depicts that *A*. *absenthia* have substantial anti-microbial ability which can be good alternate to commonly available anti-microbial substances.

*3*.*1*.*4*.*4 Interaction among treatments*. The interaction between leaf and flower; flower and stem were found to be significant, leaf and flower interaction showed negative impact while leaf and stem exhibited no interaction at P<0.05. The interaction of all the plant parts except leaf and flower was found significant which depicts that all the plant parts showed substantial antimicrobial activity in response to organic solvents in combinations as well (Table 2). This reveals that *A*. *absenthia* have appropriate ability to effectively retard the microbial attack.

**Table 2:** Comparative summary of antimicrobial properties of *A*. *absenthia* leaves, flower and stem extracts (methanol, ethanol and acetone) against all microbes under study.

	Leaves			Flower			Stem		
	Combination #	Table	Fig.	Combination #	Table	Fig.	Combination #	Table	Fig.
Methanol	1, 2, 4, 7, 15, 25	S10	S1	1, 9, 12, 17, 22 and 27	S14	S4	8, 11, 17, 21, 25 and 28	S17	S7
Ethanol	2, 4, 5, 6, 11, 20, 21, 26 and 28	S11	S2	2, 5, 6, 7, 9, 11, 20, 26 and 29	S15	S5	1, 3, 11, 12, 14, 24, 26, and 28	S18	S8
Acetone	2, 3, 5, 6, 11, 12, 13, 14, 18, 19, 26, 27 and 28	S13	S3	4, 5, 7, 8, 9, 12, 13, 14, 15, 17, 19, 21, 22, 24, 25, 27 and 29	S16	S6	1, 2, 3, 6, 8, 11, 13, 24 and 29	S19	S9

### 3.2 Anti-diabetic assays

The anti-diabetic activity of *Artemisia absinthium* was assessed by α-glucosidase activity assay. The α-glucosidase inhibitory activity was calculated and presented statistically.

#### 3.2.1 Anti-diabetic assay of leaf, stem and flower

**Fig 4:**
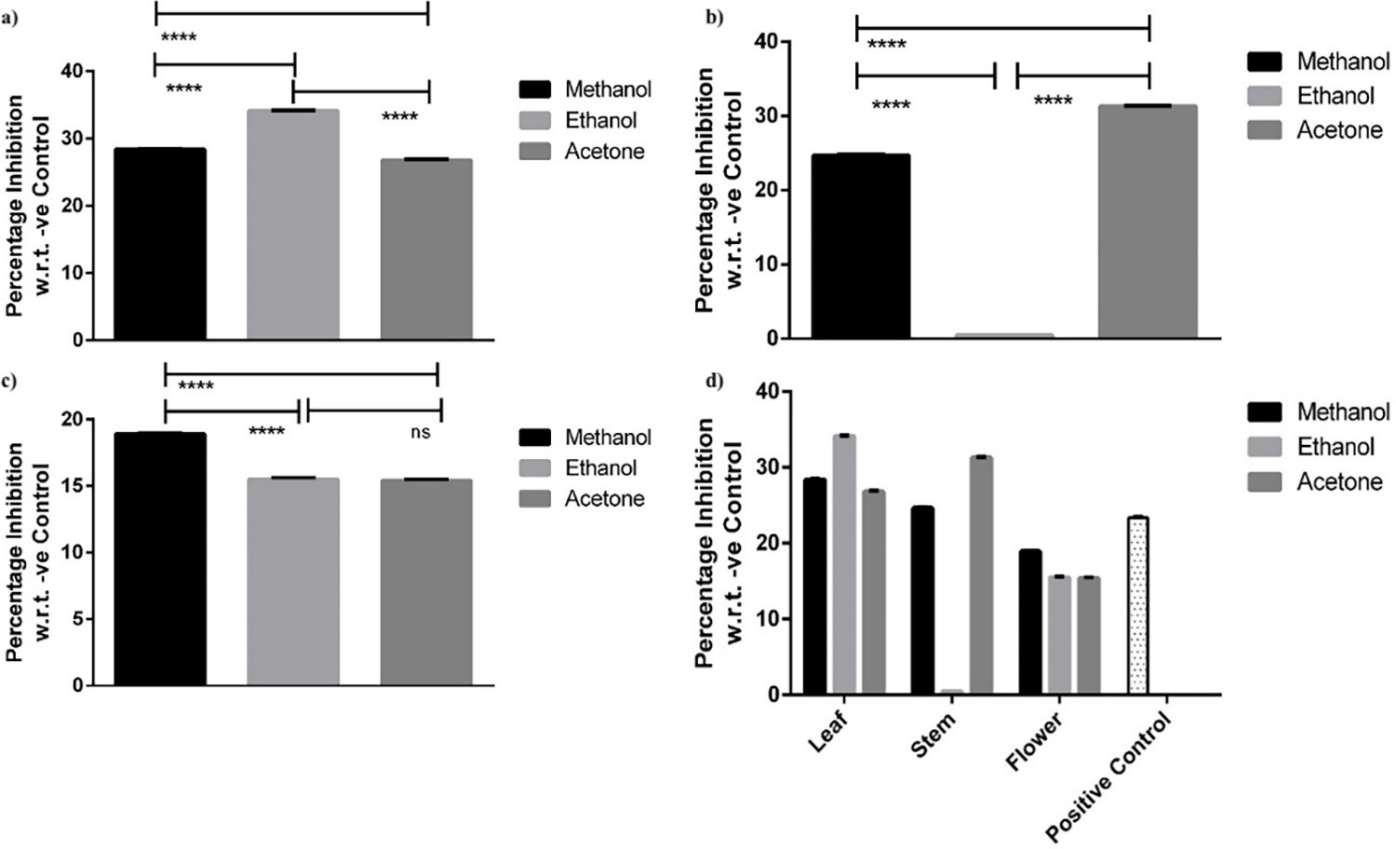
Comparison of Anti-diabetic activity of *Artemisia absinthium* of methanolic, ethanolic & acetone extracts of selected plant parts i.e. Leaf (a), Stem (b) & Flower (c). Statistical relationship of all plant parts (d) was determined by Tukey’s multiple comparison test. Data was analyzed by two-way analysis of variance (ANOVA) (P< 0.001). Bars represents the ± SEM and comparison with Positive control. * = Significant, ns = Non-Significant.

The reticence effect of leaf, stem and flower extracts of *Artemisia absinthium* dissolved in various organic solvents on α- glucosidase enzyme was investigated *in-vitro*. It was found that all the plant parts have potent inhibitory effect on activity of α- glucosidase ranging from 0.5% to 34% and proved to be anti-diabetic (Fig 4).

All the solvent-based extract of leaf showed anti hyperglycemic activity of > 26%. Maximum anti-diabetic activity was exhibited by ethanolic extract of leaf i.e. 34.1%. Whereas, 28.4% and 26.8% inhibitory capacity was found with methanol and acetone extract respectively (Fig 4A).

In case of stem extracts of *Artemisia absinthium*, acetone was found to be potent inhibitor of enzyme with 31.28% anti-diabetic activity, whereas, methanol extract unveiled 24.6% inhibition of enzyme activity (Fig 4B).

Enzyme activity of α- glucosidase was also hindered by all organic extracts of flower of *Artemisia absinthium* as depicted in Fig 4C. Statistical analysis demonstrated that among all flower extracts dissolved in organic solvents methanol was found to be significantly different than ethanol and acetone (Fig 4C). Methanolic extract showed highest (18.9%) inhibitory activity against enzyme. However, both ethanol and acetone extracts revealed similar percentage of inhibition as 15.5%.

Comparison of all plant parts for their anti-hyper glycemic activity has been represented in Fig 4D. Anti-diabetics potential of *Artemisia absinthium* has also been corelated with a synthetic alpha-glucosidase inhibitor i.e. Acarbose. It was found that all three organic extracts of leaf exhibited higher anti-diabetic activity over the positive control. Among these ethanol extract of leaf has highest percentage of inhibition 34.1%. In case of stem methanol and acetone extracts were significantly different from positive control where acetone depicted more inhibition percentage of 31.28% than acarbose. Organic extracts of flower of *Artemisia absinthium* did inhibit the activity of alpha-glucosidase but lower than the acarbose.

#### 3.2.2 Optimization of *Artemesia absenthia* and its anti-diabetic activity from various plant parts via response surface methodology

For all the plant parts of *A*. *absenthia* which came under investigation was assessed based on response surface methodology via Box-Behnken design (Table 3). The following quadratic response surface model was fitted to the data.

$$Y=\beta_{o}+\sum_{i=1}^{k}\beta_{i}F_{i}+\sum_{i=1}^{k}\beta_{\mathrm{ii}}F_{i}^{2}+\sum \sum_{i<j=1}^{k}\beta_{\mathrm{ij}}F_{i}F_{j}+\varepsilon$$

where “*Y”* was the antidiabetic activity, “*β*_0_” was the intercept constant, “*βi”*, “*βii”*, “*βij”* was the regression coefficients. “*F*_*i*_*”*, “*F*_*j*_*”* was coded values of plant parts while “ε" is error term.

**Table 3:** A design approach for determining the optimization of antidiabetic activity of *A*. *absenthia* via Box-Behnken design.

Plant Parts	Coded Symbol	Range					
		Methanol		Ethanol		Acetone	
		–1	+1	–1	+1	–1	+1
**Leaves**	A	1	1.05	1	1.05	1	1.05
**Flower**	B	1.1	1.15	1.1	1.15	1.1	1.15
**Stem**	C	1.1	1.3	1.1	1.3	1.1	1.3

*3*.*2*.*2*.*1 Antidiabetic activity in A*. *absenthia leaves in response to methanol*, *ethanol & acetone*. For the optimization of antidiabetic activity, analysis of variance was performed via quadratic model and response to methanol, ethanol and acetone along with leaf was checked in *A*. *absenthia*. The following regression equation was obtained.

Anti–diabetic Activity (Leaf) = 1.1 + 0.00625 A + 0.0375 B + 0.00625 C– 0.0125 AB + 0.00 AC + 0.0125 BC + 0.0125 A^2^ + 0.00 B^2^– 0.0125 C^2^

According to this regression equation, leaf showed positive effect as well as the anti-diabetic activity was found to be significant.

According to analysis of variance, the model fitted was found to be significant as the P-value was less than 0.05. Leaves found significant in antidiabetic properties (P<0.05). Stem was found more significant than other plant parts. This reveals that leaves of *A*. *absenthia* have appropriate ability to effectively degrade the effect of diabetes. Based on the analysis of variance applied to this model, it became obvious that this model has shown significant response at 5% level of significance while this model is also very suitable and reproducible due to having very little lack of fit (P>0.05). The coefficient of determination (R^2^) also confirms that with 96% surety this data is highly significant and can be applied under various conditions. Thus, the optimum parameters have also been defined as shown in S19 Table. The surface plots developed based upon this analysis also depicts that *A*. *absenthia* have substantial anti-diabetic ability which can be good alternate to commonly available anti-diabetics.

*3*.*2*.*2*.*2 Antidiabetic activity in A*. *absenthia flower in response to methanol*, *ethanol & acetone*. For the optimization of antidiabetic activity, analysis of variance was performed via quadratic model and response to methanol, ethanol and acetone along with flower was checked in *A*. *absenthia*. The following regression equation was obtained.

Anti–diabetic Activity (Flower) = 1.225 + 0.0125 A + 0.1 B + 0.05625 C– 0.025 AB + 0.00 AC + 0.0375 BC + 0.0125 A^2^– 0.1 B^2^– 0.0125 C^2^

According to this regression equation, flower showed positive effect as well as the anti-diabetic activity was found to be significant.

According to analysis of variance, the model fitted was found to be significant as the P-value was less than 0.05. Flowers found significant in antidiabetic properties (P<0.05). Stem was found more significant than other plant parts. This reveals that flowers of *A*. *absenthia* have appropriate ability to effectively degrade the effect of diabetes. Based on the analysis of variance applied to this model, it became obvious that this model has shown significant response at 5% level of significance while this model is also very suitable and reproducible due to having very little lack of fit (P>0.05). The coefficient of determination (R^2^) also confirms that with 97% surety this data is highly significant and can be applied under various conditions. Thus, the optimum parameters have also been defined as shown in S20 Table. The surface plots developed based upon this analysis also depicts that flowers of *A*. *absenthia* have substantial anti-diabetic ability which can be good alternate to commonly available anti-diabetics

*3*.*2*.*2*.*3 Antidiabetic activity in A*. *absenthia stem in response to methanol*, *ethanol & acetone*. For the optimization of antidiabetic activity, analysis of variance was performed via quadratic model and response to methanol, ethanol and acetone along with stem was checked in *A*. *absenthia*. The following regression equation was obtained.

Anti–diabetic Activity (Stem) = 1.225 + 0.0125 A + 0.1 B + 0.05625 C– 0.025 AB + 0.00 AC + 0.0375 BC + 0.0125 A^2^– 0.1 B^2^– 0.0125 C^2^

According to this regression equation, stem showed positive effect as well as the anti-diabetic activity was found to be significant.

According to analysis of variance, the model fitted was found to be significant as the P-value was less than 0.05. Stems found significant in antidiabetic properties (P<0.05). Stem was found more significant than other plant parts. This reveals that stems of *A*. *absenthia* have appropriate ability to effectively degrade the effect of diabetes. Based on the analysis of variance applied to this model, it became obvious that this model has shown significant response at 5% level of significance while this model is also very suitable and reproducible due to having very little lack of fit (P>0.05). The coefficient of determination (R^2^) also confirms that with 96% surety this data is highly significant and can be applied under various conditions. Thus, the optimum parameters have also been defined as shown in S21 Table. The surface plots developed based upon this analysis also depicts that stem of *A*. *absenthia* have substantial anti-diabetic ability which can be good alternate to commonly available anti-diabetics

*3*.*2*.*2*.*4 Interaction among treatments*. The interaction between leaf and flower; flower and stem were found to be significant, leaf flower interaction showed negative impact while leaf and stem exhibited no interaction at P<0.05. The interaction of all the plant parts except leaf and flower was found significant which depicts those leaves showed substantial antidiabetic activity in response to organic solvents in combinations as well at P≤0.05.

Combinations displayed in S22 Table and S10–S12 Figs in run 4, 13 and 15 showed maximum anti-diabetic properties of all plant parts of *A*. *absenthia* in response to methanol, ethanol and acetone, respectively.

### 3.3 Anti-proliferative assay

**Fig 5:**
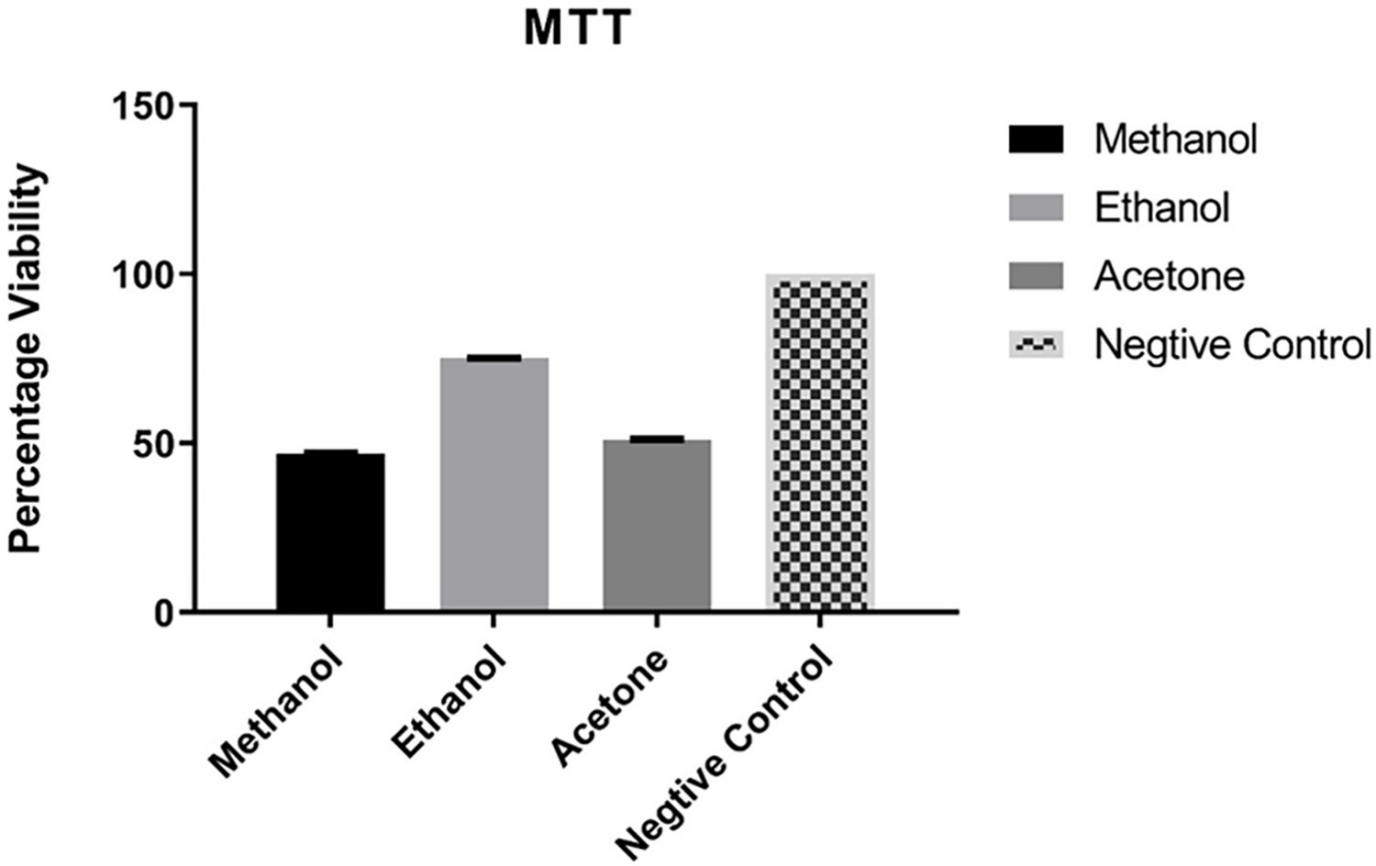
Anti-proliferative activity of leaf of *Artemisia absinthium*. Viable cell (percentage) response of Huh7 liver cancer cell line to three different kinds of organic solvents i.e., Methanol, Ethanol, Acetone. Bar represents ± SEM. Data was analyzed by one-way analysis of variance (ANOVA) (P<0.001). * = significant, ns = non-significant.

For the present study leaf extract of *Artemisia absinthium* was made in different organic solvents i.e. Ethanol, methanol and acetone. The anti-proliferative/cytotoxic effect of these organic extracts was evaluated on Hepatic liver cancer cell line Huh7). These results have been presented in term of percentage cell viability of Huh7 Cell lines in response to the leaf extracts as determined by MTT assay (Fig 5). Accordingly, all solvents significantly restrict the cell growth of Huh7 when compared with the negative control. Whereas, leaf extract made in methanol was found to be most effective showing inhibition of 53.01% as there were least viable cells in response to leaf extract. Likewise, acetone extract of same plant part also significantly suppressed the cell growth activity by 48.92% with 51.08% viable cells left over. On the other hand, ethanolic extract of leaf exhibited the viability of 75.13% cells demonstrating its least capacity of prohibition (25%), when compared to negative control. All the solvents revealed the significant anti-cancer potential of leaf extract of *Artemisia absinthium* restricting cell growth by 25% to 53%.

### 3.4 Reverse transcriptase RT-PCR analysis

Reverse transcriptase PCR was used to evaluate differential regulation of anti-cancer genes. Huh7 cell lines treated with methanol extracts of leaf have shown significant results for anti-cancerous activities compared to the control group (Fig 5), therefore was selected for further studies in RT-PCR.

#### 3.4.1 Relative expression profile of Transforming growth factor-beta (TGF-β1)

**Fig 6:**
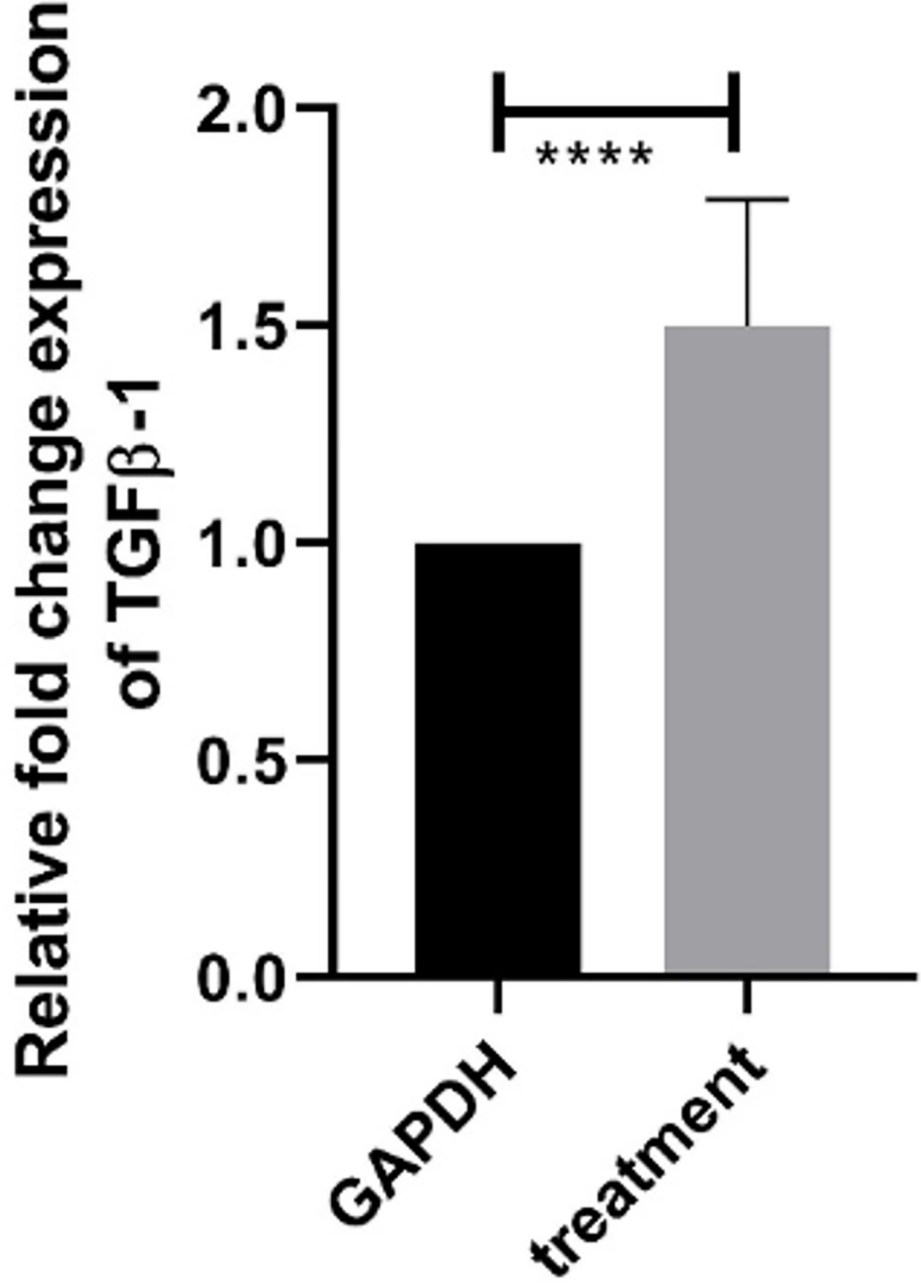
Relative expression analysis of TGF- β1. Relative fold change in expression of Huh7 cell lines treated with methanol extract of leaf of *Artemisia absinthium*. Bars are expressed as Mean ± SEM and ****, represents significant differences at P<0.0001.

Differential effects of methanol leaf extracts of *Artemisia absinthium* can be seen upon expression level of TGF-β1 in Huh7 (Fig 6). Methanolic extract of leaf of *Artemisia absinthium* resulted into significant change (approx. half fold increase) in expression level in treated Huh7 as compared to its non-treated control.

#### 3.4.2 Relative expression profile of MYC gene

**Fig 7:**
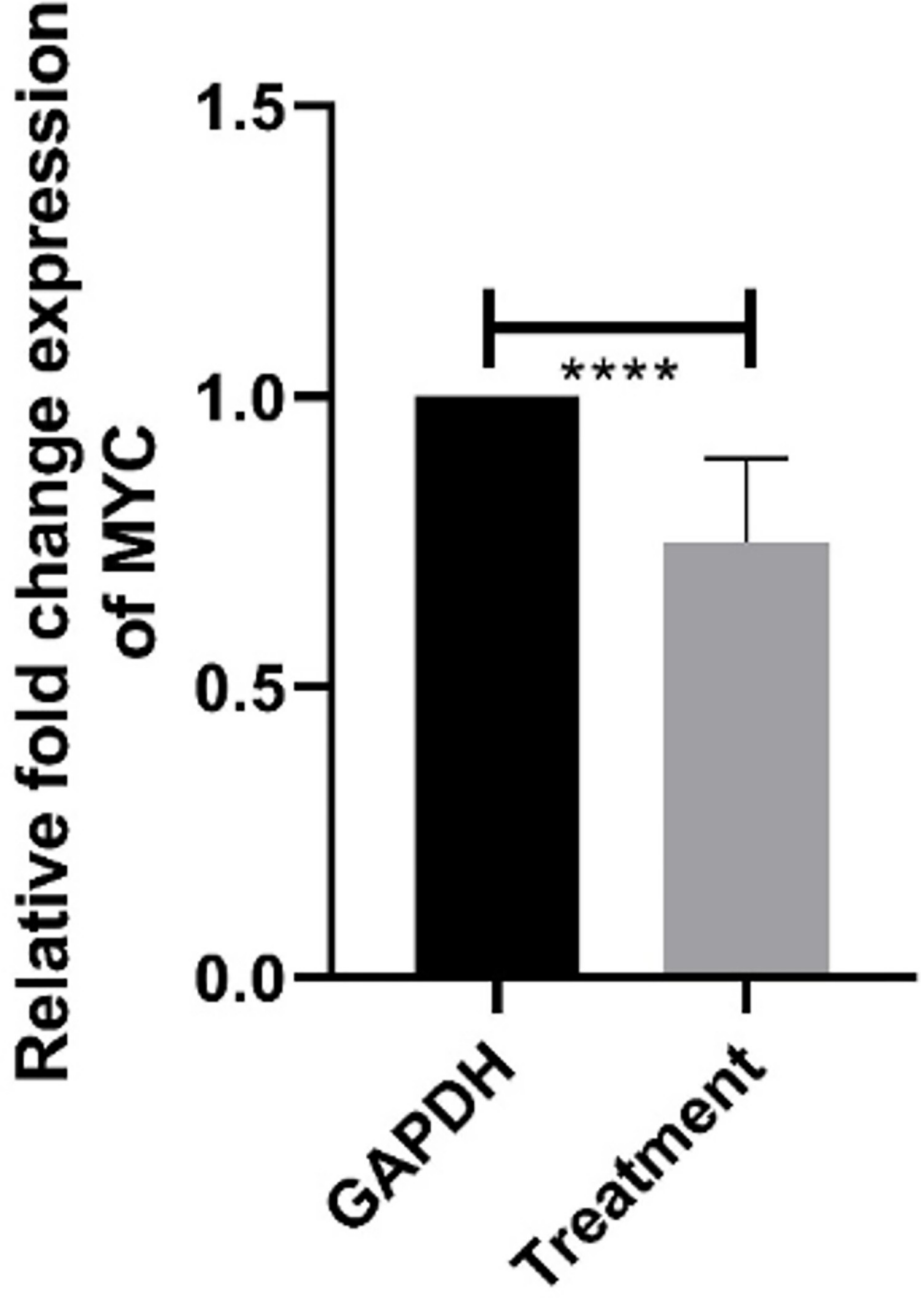
Relative expression analysis of MYC gene. Relative fold change in expression of Huh7 cell lines treated with methanol extract of leaf of *Artemisia absinthium*. Bars are expressed as Mean ± SEM and ****, represents significant differences at P<0.0001.

Differential effects of extracts of *Artemisia absinthium* upon expression level of MYC in Huh7 has been depicted in Fig 7. It is clear that application of extract resulted into altered mRNA abundance level of MYC in such a way that reduction in MYC expression level reduced the abnormal proliferation of cells. Overall, the reduction of gene expression (approx. 1/3-fold) can be attributed to the methanol extract of leaf of *Artemisia absinthium*.

### 3.5 HPLC analysis of polyphenolic compounds

The HPLC analysis of methanol & ethanol leaf extracts of *Artemisia absinthium* contains various major poly-phenolic compounds. The compounds were identified and quantified by HPLC using hydro-RP 80A (250 mm × 4.60 mm, 4 μm) column. The compounds or phenolic acids were estimated by measuring/comparing the Retention time of each compound against the synthetic chemicals/standard (Table 4). HPLC analysis showed several peaks among which 7 main peaks were prominent at different retention times ranging from 2 minutes to 22 minutes. The compounds that were identified by retention time includes Gallic acid, Catechin & Rutin representing the major fraction of phenols in both extracts of methanol & ethanol. Whereas, HB acid, Caffeic acid and Sinapic acid were also recognized at diversified retention times i.e. 7.352, 7.868 & 12.667 only in ethanol extracts. Furthermore, Ferulic acid was found only in methanol extract with retention time of 12.861 minutes. Apart from this several other unknown peaks were also recognized in both extracts by comparing retention time in already reported literature. This includes Quercitrin, Isolongefinone, β-Myrcene, Rutoside, Allo-Ocimene as reported by [12, 20, 21]. Invanescu et al. 2010 reported that compound with retention time 22.54 is Quercetin, and same was evaluated by present results of HPLC. Many other unknown compounds were also present in the results when compared with literature.

**Table 4:** Contents of major polyphenols (μg/g of dry weight) in two different extracts of *Artemisia absinthium*, based on ethanol and methanol as solvent.

Sr. No	Retention Time (min)	Name of Compound	Ethanol (μg/g dry weight of sample)	Methanol (μg/g dry weight of sample)
1	3.222	Catechin	2583.60 ± 2.62	1710.58 ± 2.31
2	7.352	HB Acid	Not found	203.05 ± 0.29
3	10.374	β-Myrcene	3784.60 ± 6.59	1210.92 ± 1.23
4	12.667	Sinapic Acid	242.15 ± 0.42	2517.21 ± 4.39
5	15.33	Isolongefinone	1408.55 ± 1.43	1018.94 ± 1.03
6	20.21	Rutoside	1060.59 ± 1.07	319.22 ± 0.32
7	22.544	Quercitrin	91.94 ± 0.27	2315.11 ± 6.77
8	29.940	Rutin	264.12 ± 0.77	1150.10 ± 5.40

## 4. Discussion

During the past few decades, there is flourishing interest to unlock the secrets of ancient plants, herbal and remedies related to them. Most of the populations in Pakistan rely on medicinal plants to seek treatment for their minor even in some cases major diseases. Some wild plants e.g. *Ephedra*, *Artimisia*, *St*. *John’s wort*, *Hippophae* are now being commonly used, beside some of them that have been grown in houses as domestic plants e.g. Garlic, Ginseng and Cumin etc [22]. Native medicinal plants from hilly areas of Pakistan have also been studied in this regard [23].

All around, the zeal of quest for antimicrobials have experienced genuine challenge of obstruction from pathogenic microorganisms to anti-infection agents. Although the expense to treat irresistible infections and to discover new anti-microbial has been, to a great extent, expanded during the previous decades, however, little achievement has been made [10]. Different kinds of solvents like water, ethanol, methanol, chloroform, acetone etc. are being used for extraction of different parts of various native plants and herbs. These plant extracts which are biochemically active are auspicious source of majority of medicines [24]. Antimicrobial effects of *Artemisia absinthium*, observed in the present study have also depicted significant results (Figs 1–3). All the bacteria under study responded differentially with plant extracts e.g. maximum inhibition (57%) was against bacilli by leaf acetone extract (Fig 1). Similarly, ethanol and acetone extract of flower imparted inhibition of 60% against *Acinetobacter* and 57% against bacilli by acetone extract only (Fig 2). Unexpectedly lowest antimicrobial effects have been found with extract of stem suggesting that each plant part has different effect with various solvents, depending upon the presence of various components i.e. Phenols and flavonoids in each part affecting its antimicrobial activity (Fig 3) [25, 26].

Diabetes mellitus is a metabolic disorder characterized by resistance to the action of insulin, insufficient insulin secretion or both. The global diabetes prevalence in 2019 is estimated to be 9.3% (463 million people), rising to 10.2% (578 million) by 2030 and 10.9% (700 million) by 2045. The prevalence is higher in urban (10.8%) than rural (7.2%) areas, and in high-income (10.4%) than low-income countries (4.0%). One in two (50.1%) people living with diabetes do not know that they have diabetes. The global prevalence of impaired glucose tolerance is estimated to be 7.5% (374 million) in 2019 and projected to reach 8.0% (454 million) by 2030 and 8.6% (548 million) by 2045 [27]. Besides, during the previous couple of years a portion of the new bioactive medications confined from plants indicated anti-diabetic action with more viability than oral hypoglycemic specialists utilized in clinical treatment.

In spite of the presence of well-known anti-diabetic drugs in the pharmaceutical market, diabetes and complications related to them kept on being a noteworthy therapeutic issue. Anti-hyperglycemic impacts of these plants are ascribed to their capacity to reestablish the capacity of pancreatic tissues by causing an expansion in insulin yield or restrain the intestinal retention of glucose or to the assistance of metabolites in insulin dependent processes. Likewise, present study has also focused on α- glucosidase inhibitory activities of *Artemisia absinthium*, and significant results have been revealed by all the plant parts i.e. Stem, Flower & Leaf (Fig 4) when dissolved in different organic solvents. Previous study has also proved that different members of family *Asteraceae* exhibits a significant anti-hyperglycemic effect [19, 28].

In RSM, many designs are used to optimize the variables including Box–Behnken (BBD), Central Composite Design (CCD), and Doehlert designs [29]. The contour and surface plots developed after the analysis display optimal conditions of the applied treatments. The experimental variables and region should be properly defined as the number of variables that determine the order of the model that is either factorial or quadratic. These plots help in the visual inspection of the experiment. For quadratic models, the minimum–maximum and critical points are needed to determine the effectiveness of the mathematical function. This method is advantageous because it can properly describe a huge number of variables and their interactions [30].

More than 400 plant species having hypoglycemic activity have been available in literature, and most of plants contain glycosides, alkaloids, terpenoids, flavonoids, carotenoids, etc., that are frequently implicated as having anti-diabetic effect. Present studied plant parts i.e. *Artemisia absinthium* are also suggesting, having that potential and compounds like glycosides and flavonoids. Confirmation of these specific components i.e. phenols in the selected plant parts was concluded by HPLC analysis, measuring/comparing the Retention time of each compound against the synthetic chemicals/standard as mentioned in the results (Table 4). More than 7 main peaks were prominent at different retention times validating the presence of Gallic acid, Catechin & Rutin as a major fraction and other compounds mentioned in the results in the extract of leaf ethanol & methanol.

Present study illustrates that *Artemisia absinthium* has tremendous therapeutic potential owing to the abundance of phytochemicals like phenols and flavonoids which exhibit innumerable biological properties, keeping the in view of that leaf extract of *Artemisia absinthium* dissolved in selected organic solvents was also tested for antiproliferative activity through MTT assay and results depicted that extract with all three solvents have significantly restricted the cell growth, but leaf methanol was best which restricts cell growth by 53%.

Under physiological conditions, cells get their fate deciding through signs from their tissue surrounding environment, principally as polypeptide development factors. Development components i.e. growth factors are mostly engaged with evolvement of protection from restorative regimens, which broadens the jobs for polypeptide elements to exceptionally late periods of tumor progression and opportunities for malignant cancer growth treatment [31]. Different growth factors including interleukin family (IL), transforming growth factor (TGF), epidermal growth factor (EGF), vascular endothelial growth factor (VEGF), tumor necrosis factor alpha (TNF-α), transforming growth factor (TGF-β) i.e. (TGF-β1, TGF-β2, and TGF-β3) and some other growth regulators like MYC, JUN Caspase 8, as well are well known for their important role in the disturbance of normal cell progression and turning those cells to cancer cells [31, 32].

Despite the fact that there is an expanding understanding on how TGF-β1 signaling is related with tumor progression in hepatocellular carcinoma, it isn’t evident whether TGF-β1 signaling is restricted to a specific subgroup of patients with hepatocellular carcinoma or is a key driver of hepatocellular carcinoma during the whole tumorigenesis of hepatocellular carcinoma [33]. Inhibitors of the TGF-β1 signaling have been appeared for the blockage of hepatocellular carcinoma development and its progression by modulating he pathway of EMT in various exploratory models. Similar change in expression of TGF-β1 have been revealed in Huh7 cells progression when treated with methanolic extract of leaf of *Artemisia absinthium* (Fig 6).

Present Studies revealed that apoptotic ability of TGF-β1is accelerated when cells treated with the extract of *Artemisia absinthium* by inhibiting the EGFR and PK13/AKT pathway that leads to cancer activity in normal cells. Previous data suggested that apoptosis induced by TGF-β in the normal cell is intermediated by upregulated pathway of the NADPH oxidase NOX4 but responded by transactivation of another pathway called the epidermal growth factor receptor (EGFR) pathway [33]. Furthermore, attenuation in TGF-β-induced EGFR transactivation and activation of the PI3K/AKT pathway leads to the formation of unwanted growth of cells called tumor. Likewise, extract of *Artemisia absinthium* regulated the expression of TGF-β1 in a way that its up regulation inhibited the growth of cells in Human liver cancer Huh7 cell lines.

The MYC, a proto-oncogene is frequently activated in human cancers through a variety of mechanisms. Its deregulated expression contributes to the progression of tumorigenesis in the normal cells [34]. Similar experiments in the present study revealed the down-regulated expression of MYC in Huh7 cells by regulating levels of CDK4 in cell cycle under the treatment with Leaf methanolic extract (Fig 7). MYC worked as promoter of mRNA levels of CDK4, inducing rapid increase in it [35]. The transcriptional program initiated by MYC must be changed from that of a cell in resting phase, in which expression of cyclin-dependent kinase inhibitors are mostly elevated and expression of cyclin-dependent kinases are suppressed suggesting that MYC simply amplified the cell progression by altering the normal cell cycle and converting it into a tumor cell. Present results with *Artemisia absinthium* exposed the significant change in expression of MYC by lowering its expression levels in the treated cells. This altered expression is attributed that MYC simply amplified the genes which are already suppressed due to its overexpression.

Therefore, present studies suggested that extracts of leaf of *Artemisia absinthium* have that potential to act differently in a way to suppress tumor growth, or cancer cell growth in liver cancer cells by attenuating the expression of several genes and pathways involved in it, calling for further ways/ studies to explore more pathways and genes involved in this uncontrolled process called Cancer.

## Supporting information

S1 Fig Response surface plot for antimicrobial activity in leaf of Artemsia absenthia in response to methanol between a) Klebsiella and Acenitobacter b) Klebsiella and Gram negative Bacilli c) Klebsiella and S. aureus d) Gram negative Bacilli and Acenitobacter e) Acenitobacter and S. aureus f) Gram negative Bacilli and S. aureus.
(PDF)

S2 Fig Response surface plot for antimicrobial activity in leaf of Artemsia absenthia in response to ethanol between a) Klebsiella and Acenitobacter b) Klebsiella and Gram negative Bacilli c) Klebsiella and S. aureus d) Gram negative Bacilli and Acenitobacter e) Acenitobacter and S. aureus f) Gram negative Bacilli and S. aureus.
(PDF)

S3 Fig Response surface plot for antimicrobial activity in leaf of Artemsia absenthia in response to acetone between a) Klebsiella and Acenitobacter b) Klebsiella and Gram negative Bacilli c) Klebsiella and S. aureus d) Gram negative Bacilli and Acenitobacter e) Acenitobacter and S. aureus f) Gram negative Bacilli and S. aureus.
(PDF)

S4 Fig Response surface plot for antimicrobial activity in flower of Artemsia absenthia in response to methanol between a) Klebsiella and Acenitobacter b) Klebsiella and Gram negative Bacilli c) Klebsiella and S. aureus d) Gram negative Bacilli and Acenitobacter e) Acenitobacter and S. aureus f) Gram negative Bacilli and S. aureus.
(PDF)

S5 Fig Response surface plot for antimicrobial activity in flower of Artemsia absenthia in response to ethanol between a) Klebsiella and Acenitobacter b) Klebsiella and Gram negative Bacilli c) Klebsiella and S. aureus d) Gram negative Bacilli and Acenitobacter e) Acenitobacter and S. aureus f) Gram negative Bacilli and S. aureus.
(PDF)

S6 Fig Response surface plot for antimicrobial activity in flower of Artemsia absenthia in response to acetone between a) Klebsiella and Acenitobacter b) Klebsiella and Gram negative Bacilli c) Klebsiella and S. aureus d) Gram negative Bacilli and Acenitobacter e) Acenitobacter and S. aureus f) Gram negative Bacilli and S. aureus.
(PDF)

S7 Fig Response surface plot for antimicrobial activity in stem of Artemsia absenthia in response to methanol between a) Klebsiella and Acenitobacter b) Klebsiella and Gram negative Bacilli c) Klebsiella and S. aureus d) Gram negative Bacilli and Acenitobacter e) Acenitobacter and S. aureus f) Gram negative Bacilli and S. aureus.
(PDF)

S8 Fig Response surface plot for antimicrobial activity in stem of Artemsia absenthia in response to ethanol between a) Klebsiella and Acenitobacter b) Klebsiella and Gram negative Bacilli c) Klebsiella and S. aureus d) Gram negative Bacilli and Acenitobacter e) Acenitobacter and S. aureus f) Gram negative Bacilli and S. aureus.
(PDF)

S9 Fig Response surface plot for antimicrobial activity in stem of Artemsia absenthia in response to acetone between a) Klebsiella and Acenitobacter b) Klebsiella and Gram negative Bacilli c) Klebsiella and S. aureus d) Gram negative Bacilli and Acenitobacter e) Acenitobacter and S. aureus f) Gram negative Bacilli and S. aureus.
(PDF)

S10 Fig Response surface plot for antidiabetic activity of Artemsia absenthia in response to methanol between a) leaf and stem b) leaf and flower and c) stem and flower.
(PDF)

S11 Fig Response surface plot for antidiabetic activity of Artemsia absenthia in response to ethanol between a) leaf and stem b) leaf and flower and c) stem and flower.
(PDF)

S12 Fig Response surface plot for antidiabetic activity of Artemsia absenthia in response to acetone between a) leaf and stem b) leaf and flower and c) stem and flower.
(PDF)

S1 Table Analysis of variance (ANOVA) for antimicrobial activity of *A*. *absenthia* leaf in response to methanol. (DOCX)

S2 Table Analysis of variance (ANOVA) for antimicrobial activity of *A*. *absenthia* leaf in response to ethanol. (DOCX)

S3 Table Analysis of variance (ANOVA) for antimicrobial activity of *A*. *absenthia* leaf in response to acetone. (DOCX)

S4 Table Analysis of variance (ANOVA) for antimicrobial activity of *A*. *absenthia* flower in response to methanol. (DOCX)

S5 Table Analysis of variance (ANOVA) for antimicrobial activity of *A*. *absenthia* flower in response to ethanol. (DOCX)

S6 Table Analysis of variance (ANOVA) for antimicrobial activity of *A*. *absenthia* flower in response to acetone. (DOCX)

S7 Table Analysis of variance (ANOVA) for antimicrobial activity of *A*. *absenthia* stem in response to methanol. (DOCX)

S8 Table Analysis of variance (ANOVA) for antimicrobial activity of *A*. *absenthia* stem in response to ethanol. (DOCX)

S9 Table Analysis of variance (ANOVA) for antimicrobial activity of *A*. *absenthia* stem in response to acetone. (DOCX)

S10 Table Experimental and predicted values of anti-microbial activity of *Artemesia absenthia* leaf in response to methanol. (DOCX)

S11 Table Experimental and predicted values of anti-microbial activity of *Artemesia absenthia* leaf in response to ethanol. (DOCX)

S12 Table Experimental and predicted values of anti-microbial activity of *Artemesia absenthia* leaf in response to acetone. (DOCX)

S13 Table Experimental and predicted values of anti-microbial activity of *Artemesia absenthia* flower in response to methanol. (DOCX)

S14 Table Experimental and predicted values of anti-microbial activity of *Artemesia absenthia* flower in response to ethanol. (DOCX)

S15 Table Experimental and predicted values of anti-microbial activity of *Artemesia absenthia* flower in response to acetone. (DOCX)

S16 Table Experimental and predicted values of anti-microbial activity of *Artemesia absenthia* stem in response to methanol. (DOCX)

S17 Table Experimental and predicted values of anti-microbial activity of *Artemesia absenthia* stem in response to ethanol. (DOCX)

S18 Table Experimental and predicted values of anti-microbial activity of *Artemesia absenthia* stem in response to acetone. (DOCX)

S19 Table Analysis of variance (ANOVA) for antidiabetic activity of *A*. *absenthia* (Leaves, stem & flower) in response to methanol. (DOCX)

S20 Table Analysis of variance (ANOVA) for antidiabetic activity of *A*. *absenthia* (Leaves, stem & flower) in response to ethanol. (DOCX)

S21 Table Analysis of variance (ANOVA) for antidiabetic activity of *A*. *absenthia* (Leaves, stem & flower) in response to acetone. (DOCX)

S22 Table Experimental and predicted values for anti–diabetic activity of *A*. *absenthia* (Leaves, stem & flower). (DOCX)
