# Supplementary figures and images for: Pharmacological activities of Artemisia absinthium and control of hepatic cancer by expression regulation of TGFβ1 and MYC genes
Source: PLoS One. 2023 Apr 13;18(4):e0284244. doi: 10.1371/journal.pone.0284244 (PMC10101520; doi:10.1371/journal.pone.0284244)

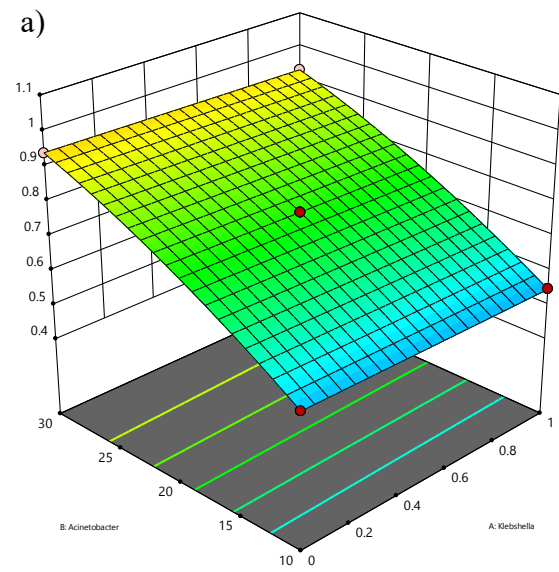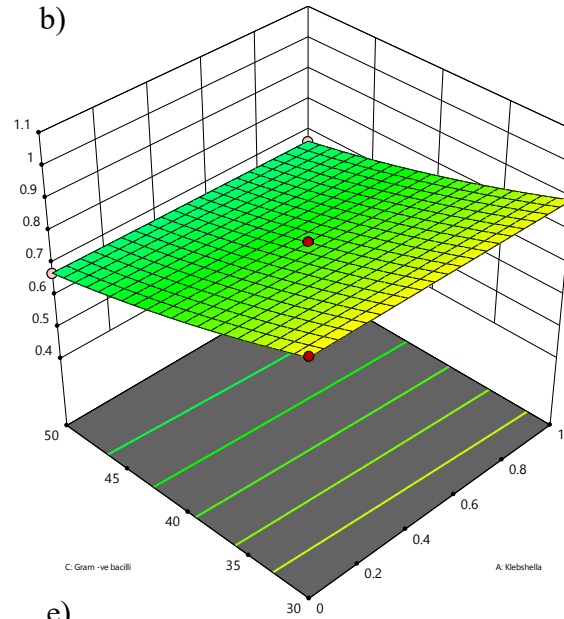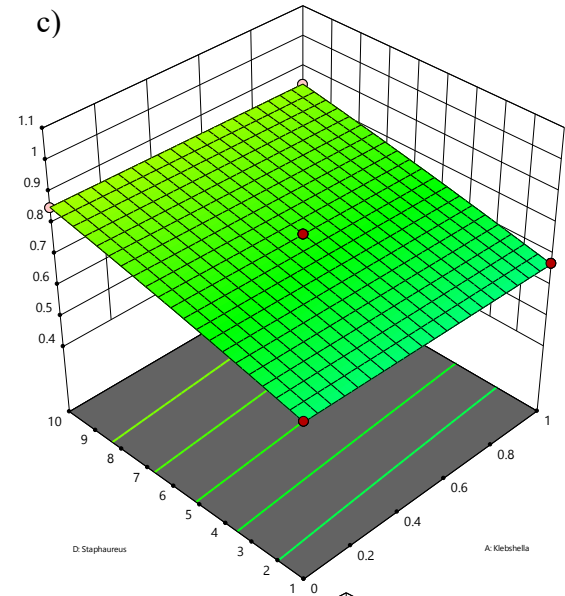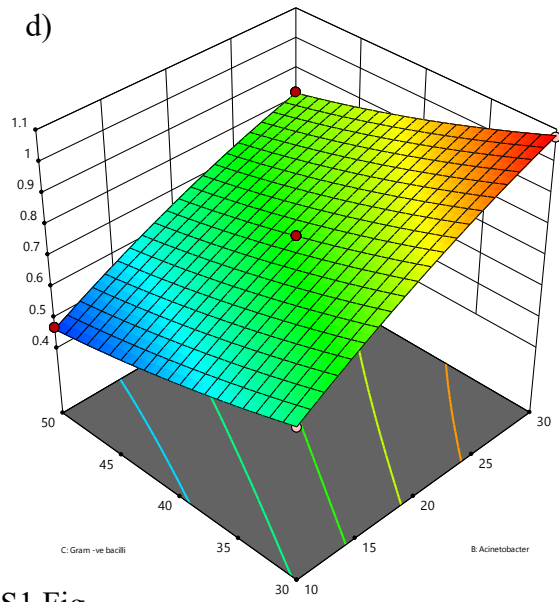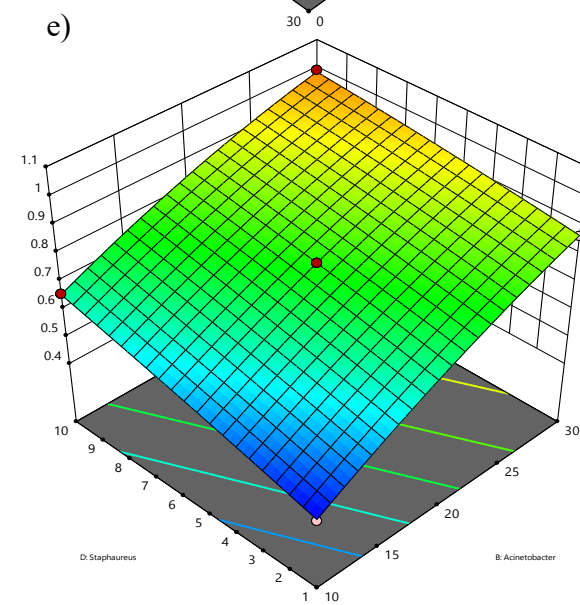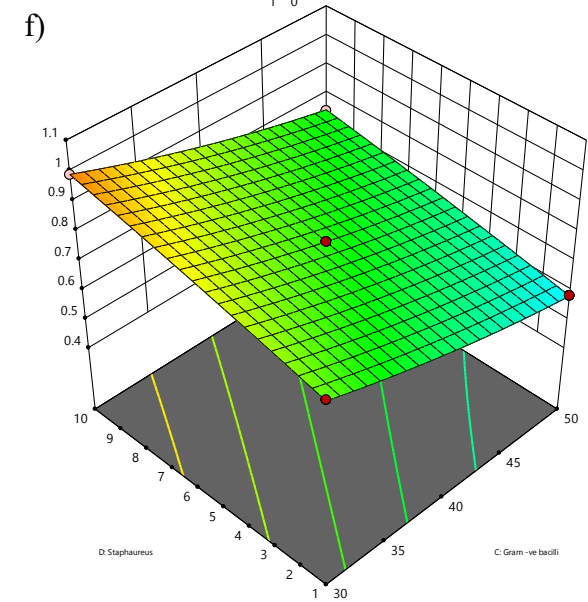

S1 Fig.

Supplement: S1 Fig — Response surface plot for antimicrobial activity in leaf of Artemsia absenthia in response to methanol between a) Klebsiella and Acenitobacter b) Klebsiella and Gram negative Bacilli c) Klebsiella and S. aureus d) Gram negative Bacilli and Acenitobacter e) Acenitobacter and S. aureus f) Gram negative Bacilli and S. aureus. (PDF) [file pone.0284244.s001.pdf]

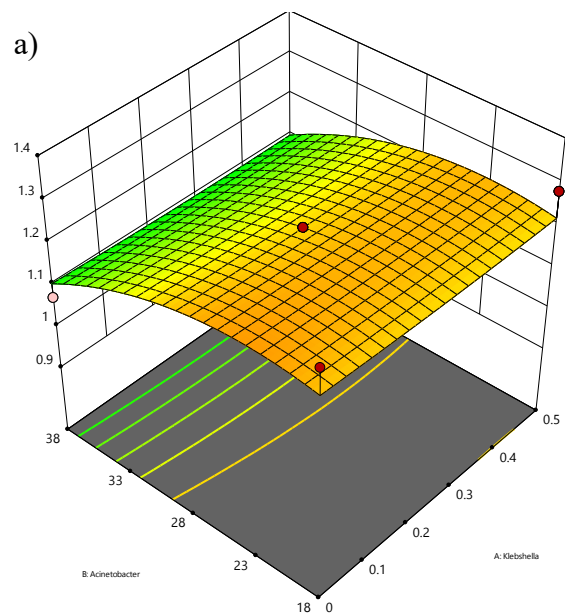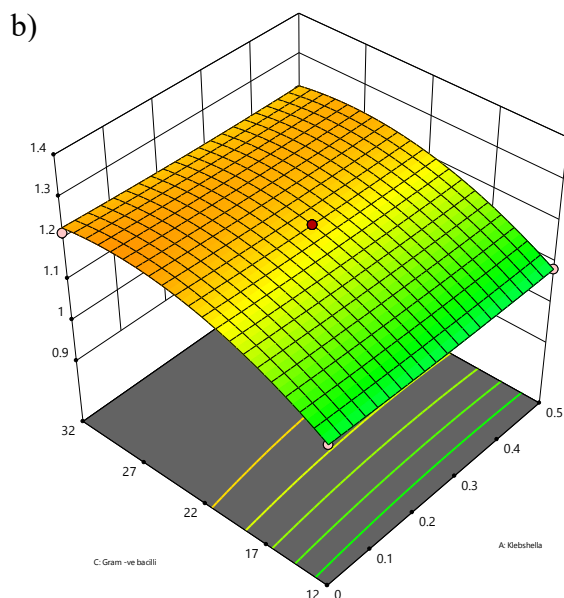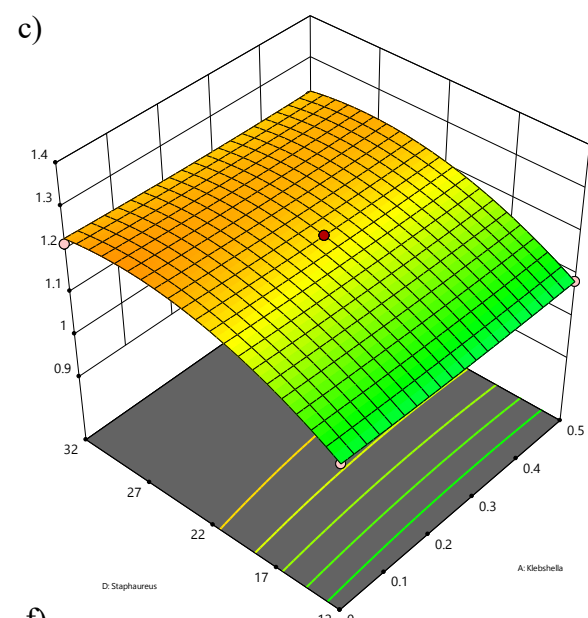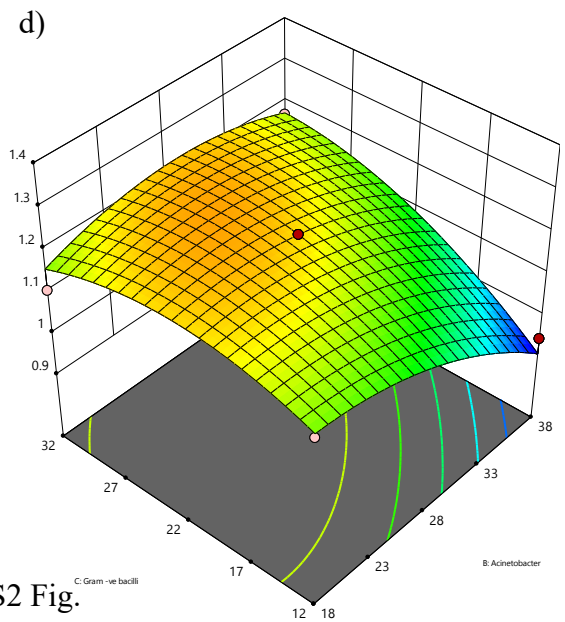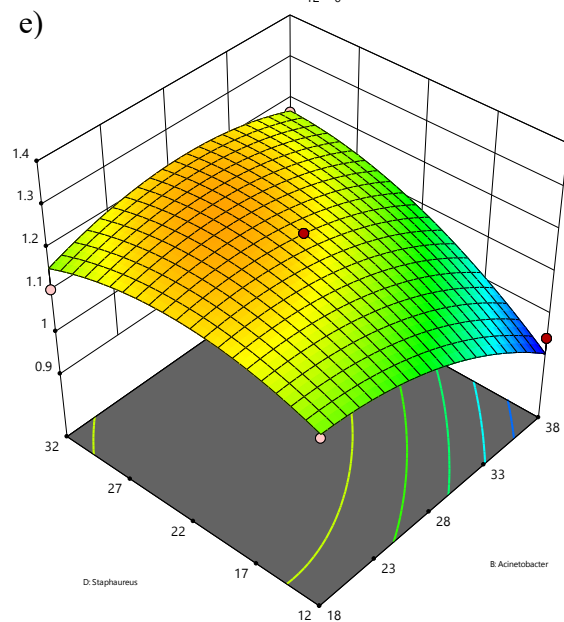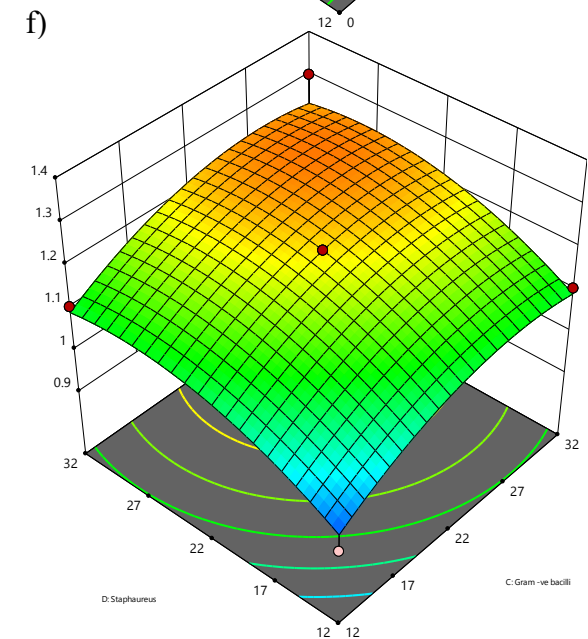

S2 Fig.

Supplement: S2 Fig — Response surface plot for antimicrobial activity in leaf of Artemsia absenthia in response to ethanol between a) Klebsiella and Acenitobacter b) Klebsiella and Gram negative Bacilli c) Klebsiella and S. aureus d) Gram negative Bacilli and Acenitobacter e) Acenitobacter and S. aureus f) Gram negative Bacilli and S. aureus. (PDF) [file pone.0284244.s002.pdf]

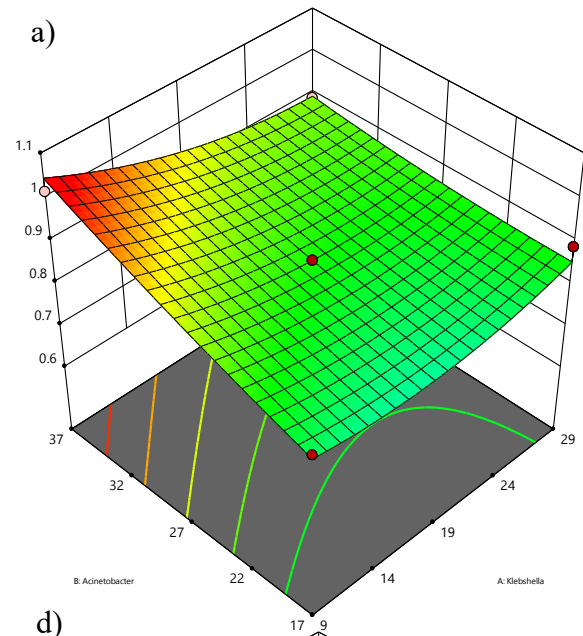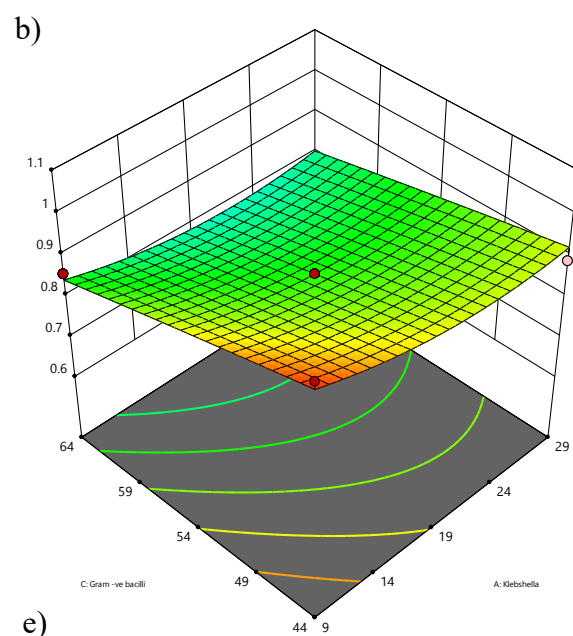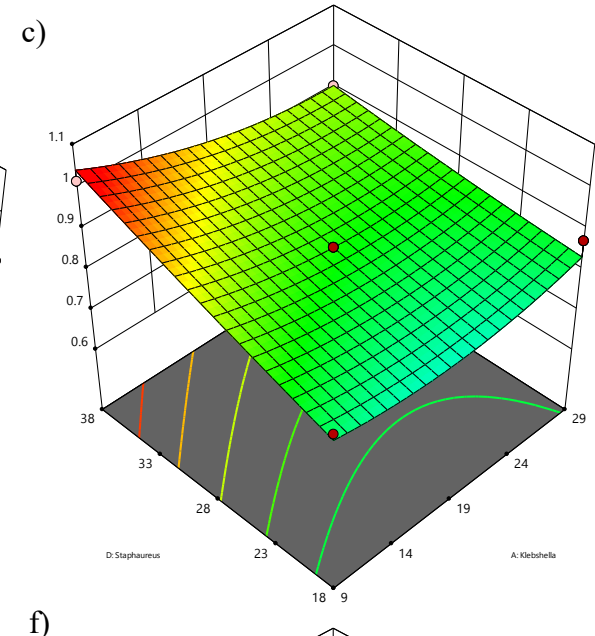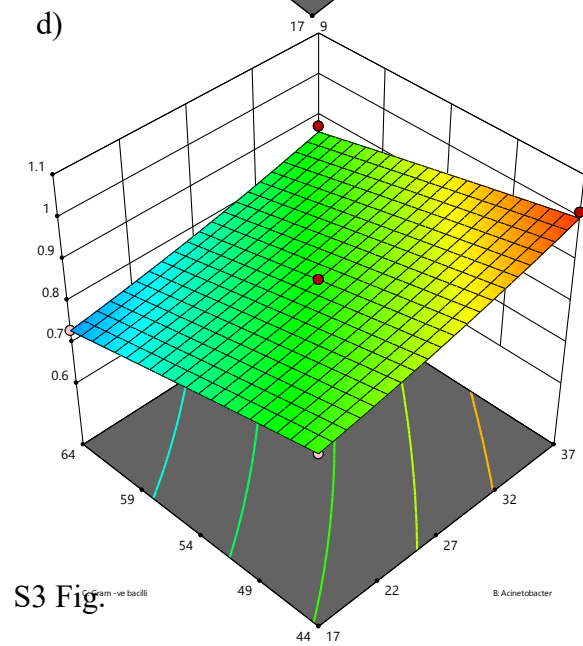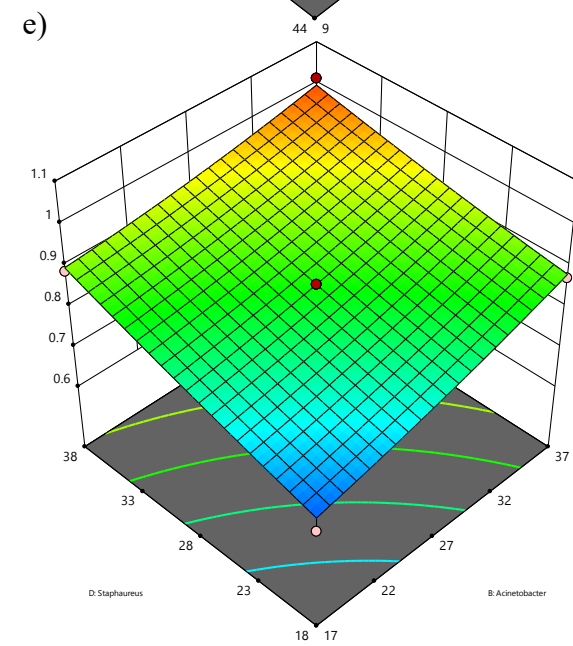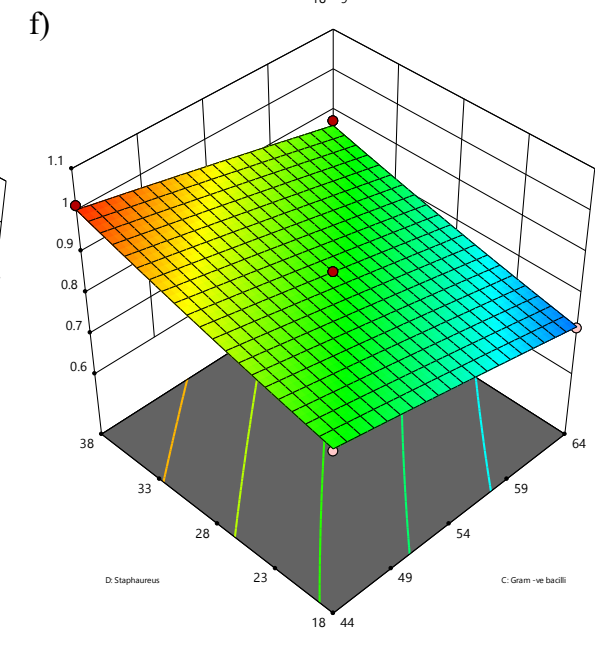

S3 Fig.

Supplement: S3 Fig — Response surface plot for antimicrobial activity in leaf of Artemsia absenthia in response to acetone between a) Klebsiella and Acenitobacter b) Klebsiella and Gram negative Bacilli c) Klebsiella and S. aureus d) Gram negative Bacilli and Acenitobacter e) Acenitobacter and S. aureus f) Gram negative Bacilli and S. aureus. (PDF) [file pone.0284244.s003.pdf]

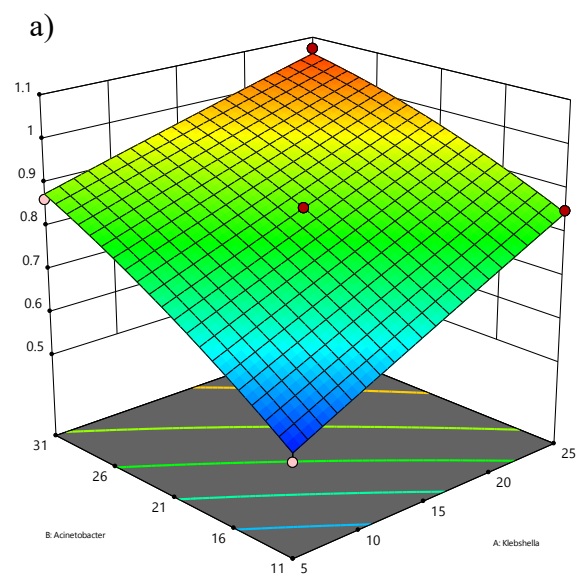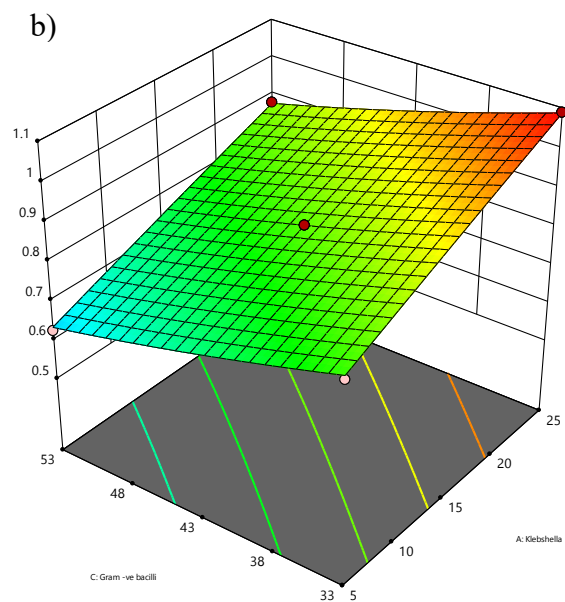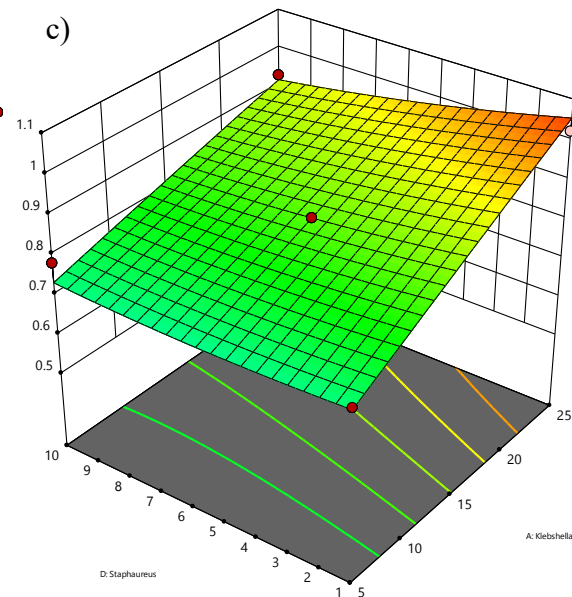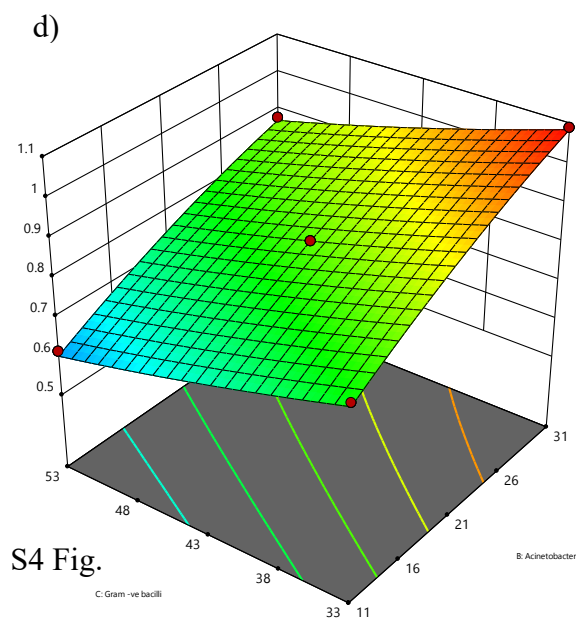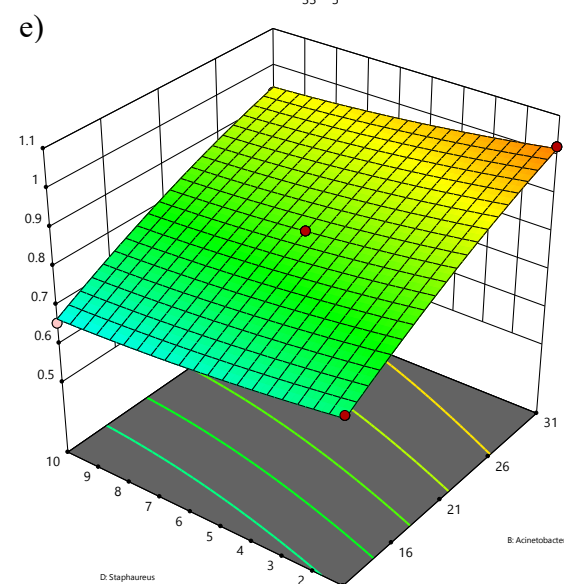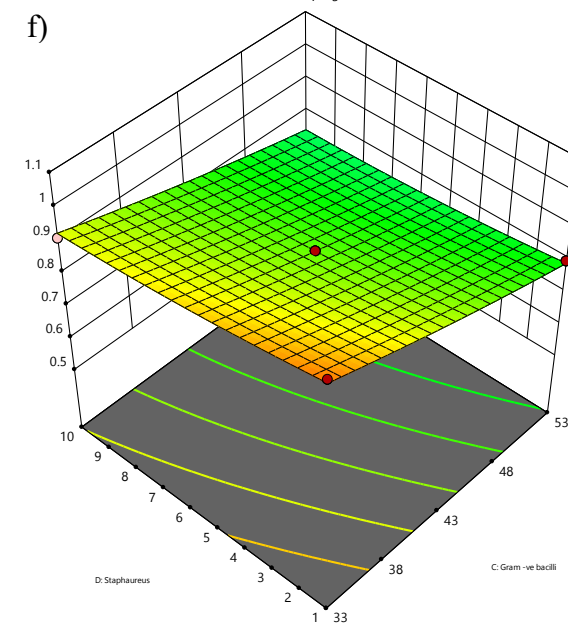

S4 Fig.

Supplement: S4 Fig — Response surface plot for antimicrobial activity in flower of Artemsia absenthia in response to methanol between a) Klebsiella and Acenitobacter b) Klebsiella and Gram negative Bacilli c) Klebsiella and S. aureus d) Gram negative Bacilli and Acenitobacter e) Acenitobacter and S. aureus f) Gram negative Bacilli and S. aureus. (PDF) [file pone.0284244.s004.pdf]

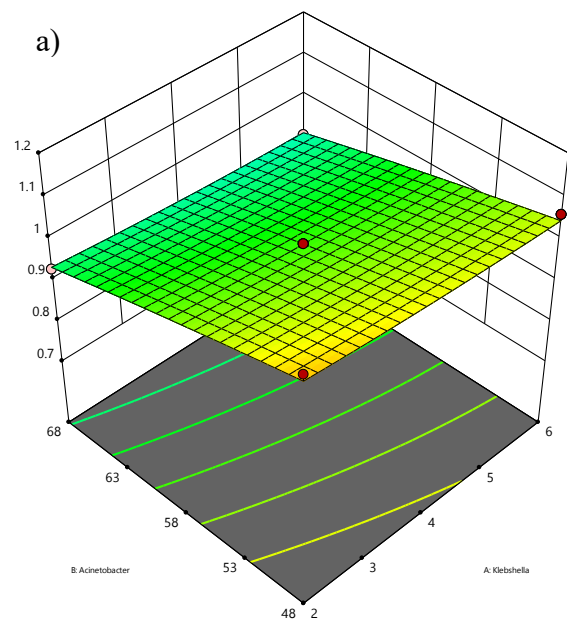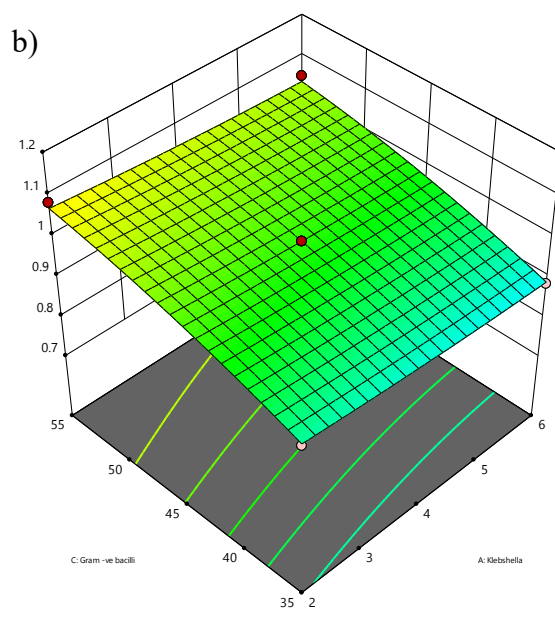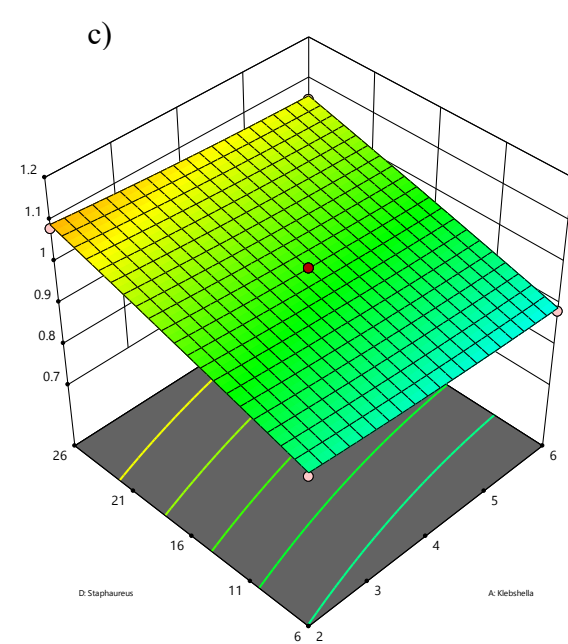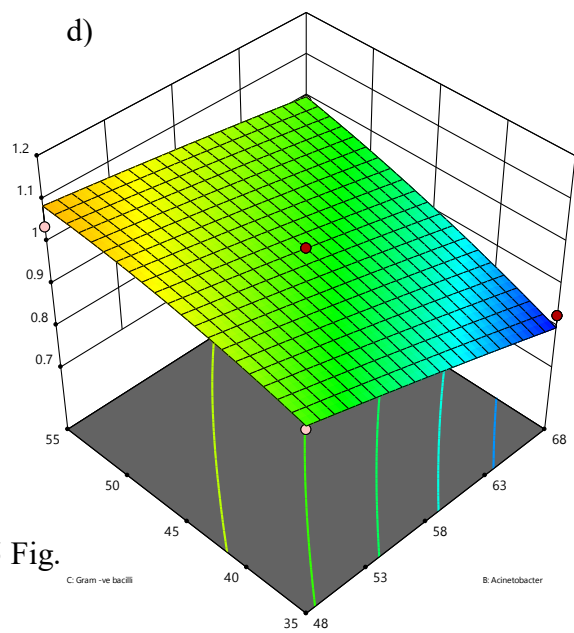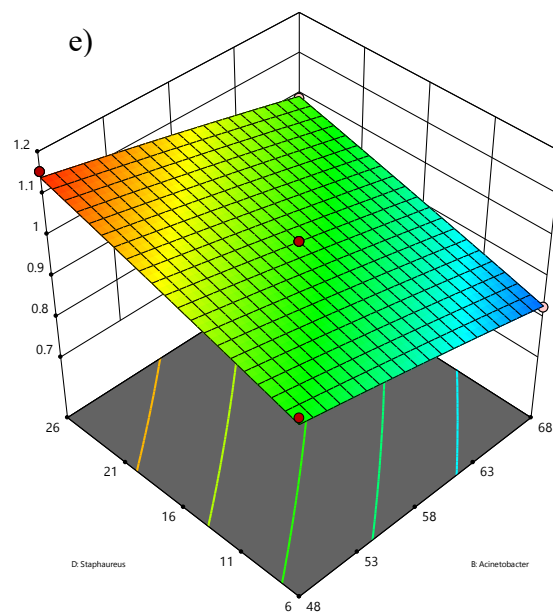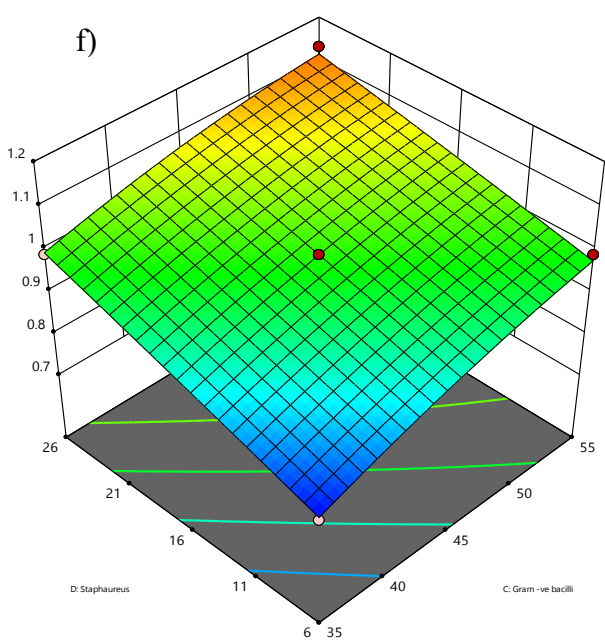

S5 Fig.

Supplement: S5 Fig — Response surface plot for antimicrobial activity in flower of Artemsia absenthia in response to ethanol between a) Klebsiella and Acenitobacter b) Klebsiella and Gram negative Bacilli c) Klebsiella and S. aureus d) Gram negative Bacilli and Acenitobacter e) Acenitobacter and S. aureus f) Gram negative Bacilli and S. aureus. (PDF) [file pone.0284244.s005.pdf]

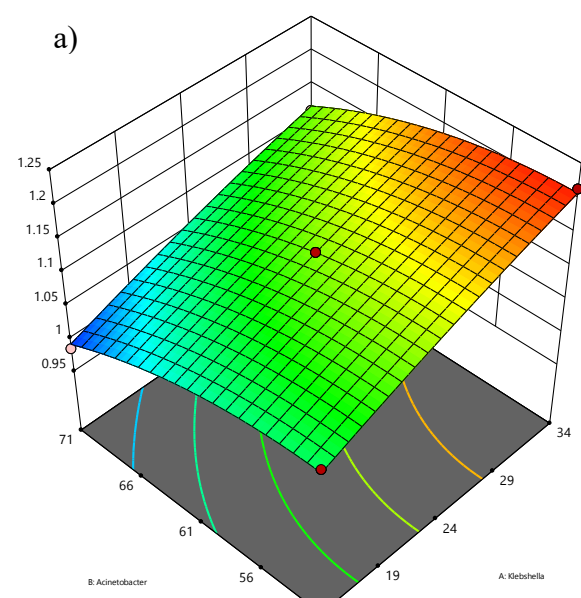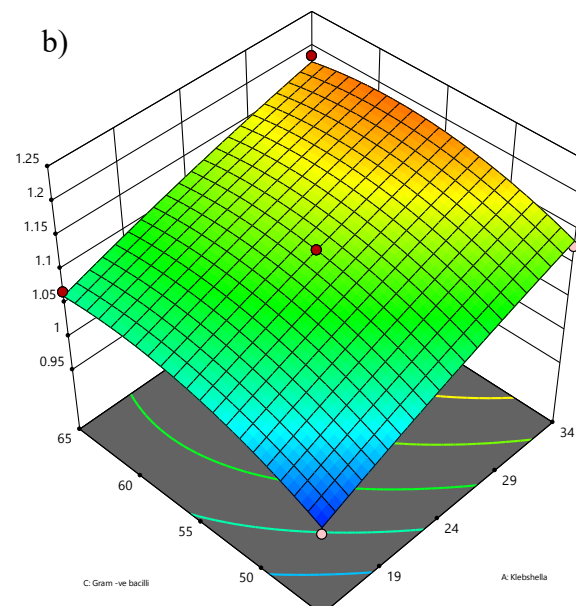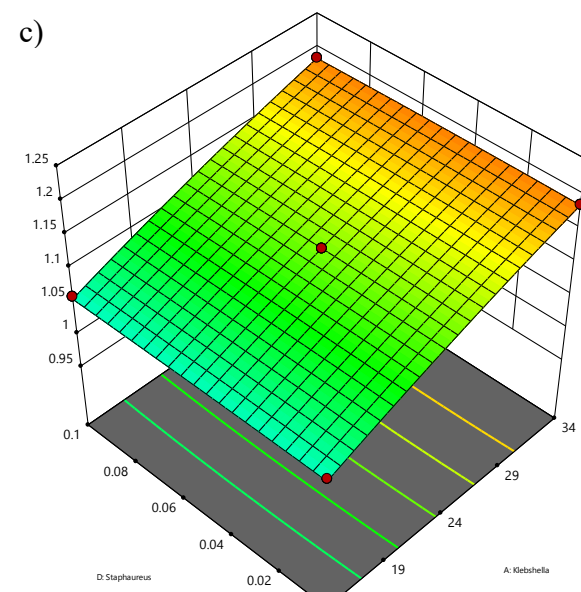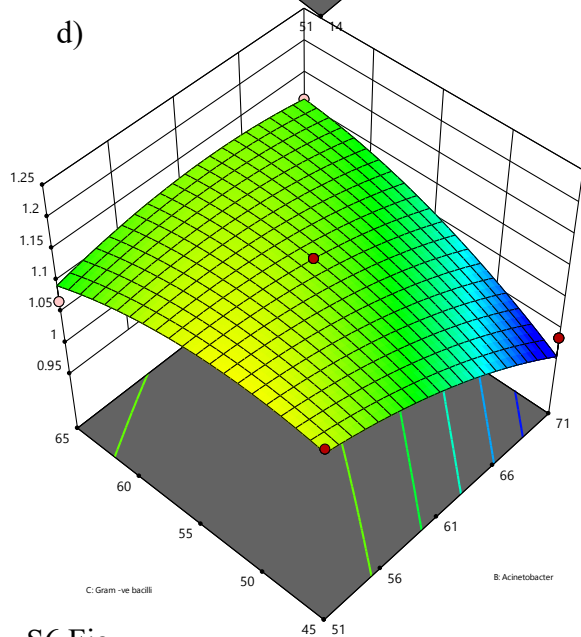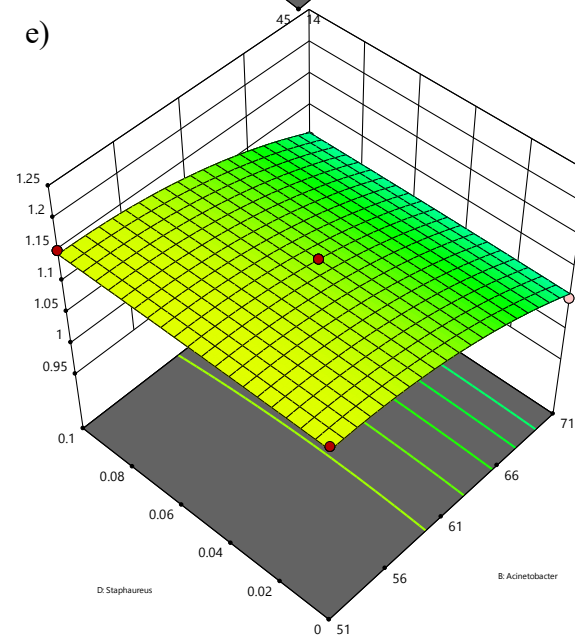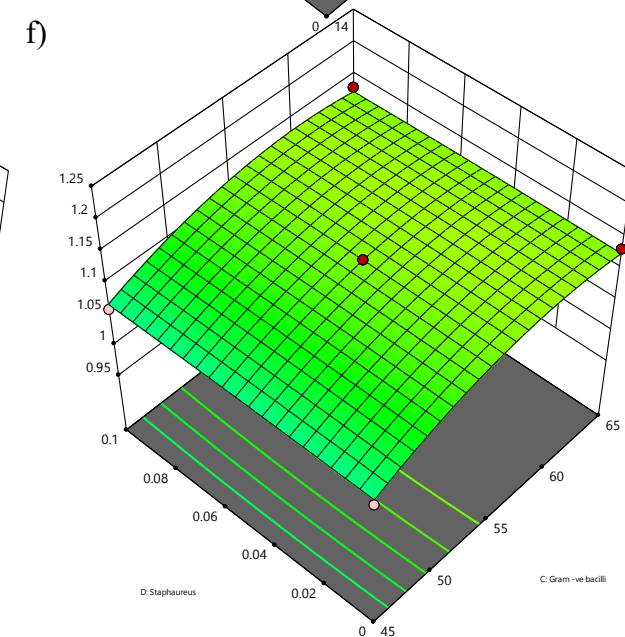

S6 Fig.

Supplement: S6 Fig — Response surface plot for antimicrobial activity in flower of Artemsia absenthia in response to acetone between a) Klebsiella and Acenitobacter b) Klebsiella and Gram negative Bacilli c) Klebsiella and S. aureus d) Gram negative Bacilli and Acenitobacter e) Acenitobacter and S. aureus f) Gram negative Bacilli and S. aureus. (PDF) [file pone.0284244.s006.pdf]

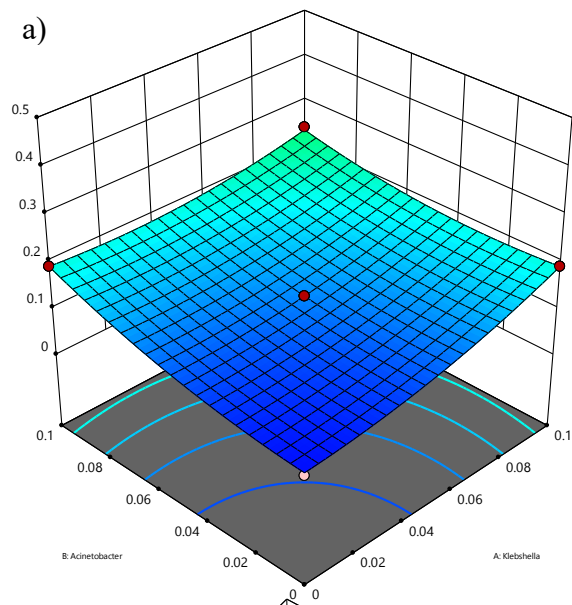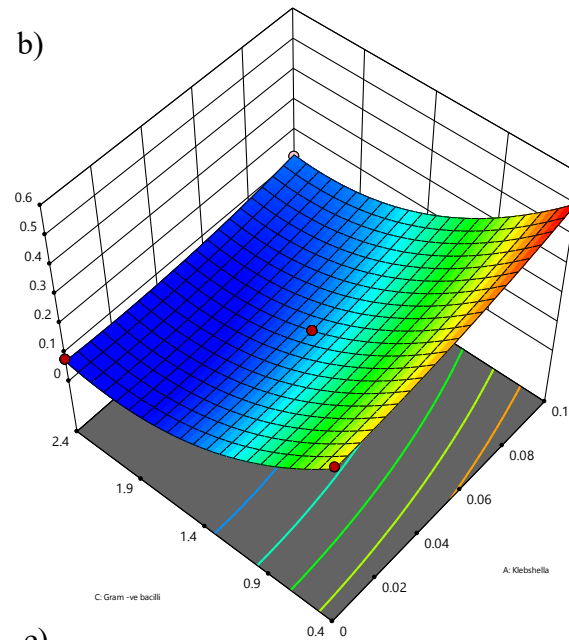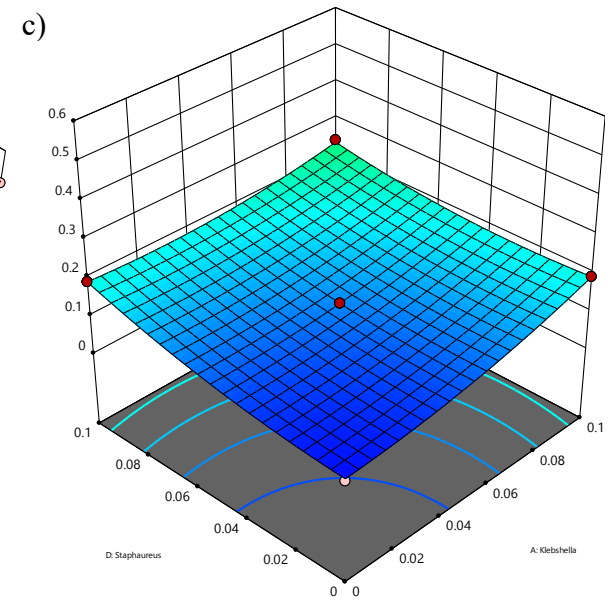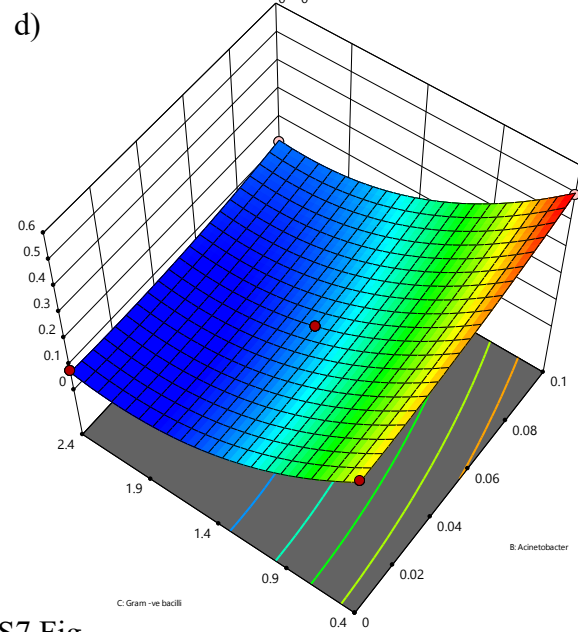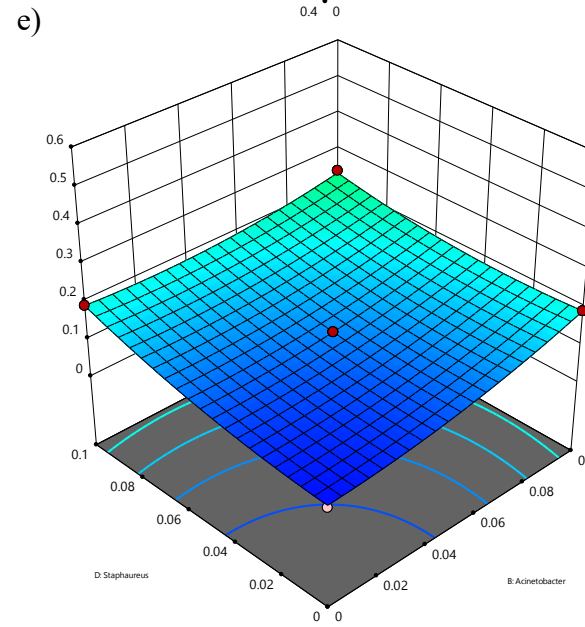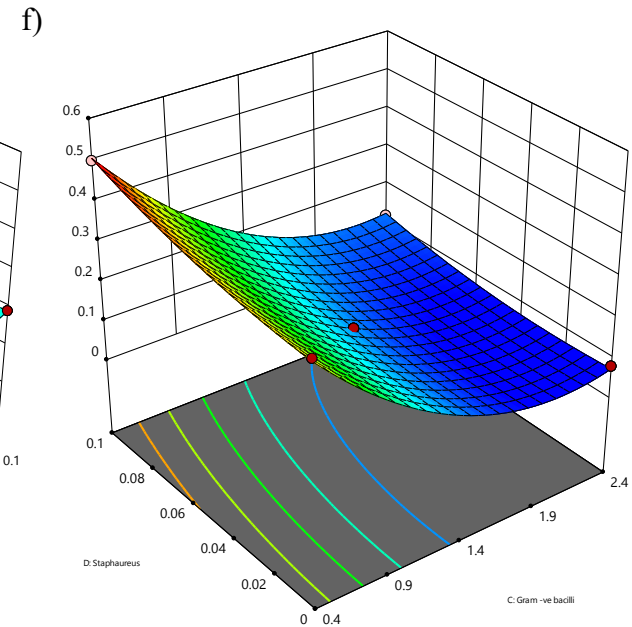

S7 Fig.

Supplement: S7 Fig — Response surface plot for antimicrobial activity in stem of Artemsia absenthia in response to methanol between a) Klebsiella and Acenitobacter b) Klebsiella and Gram negative Bacilli c) Klebsiella and S. aureus d) Gram negative Bacilli and Acenitobacter e) Acenitobacter and S. aureus f) Gram negative Bacilli and S. aureus. (PDF) [file pone.0284244.s007.pdf]

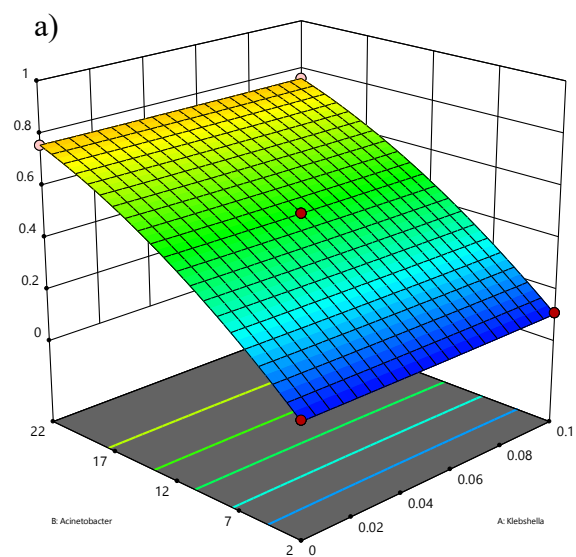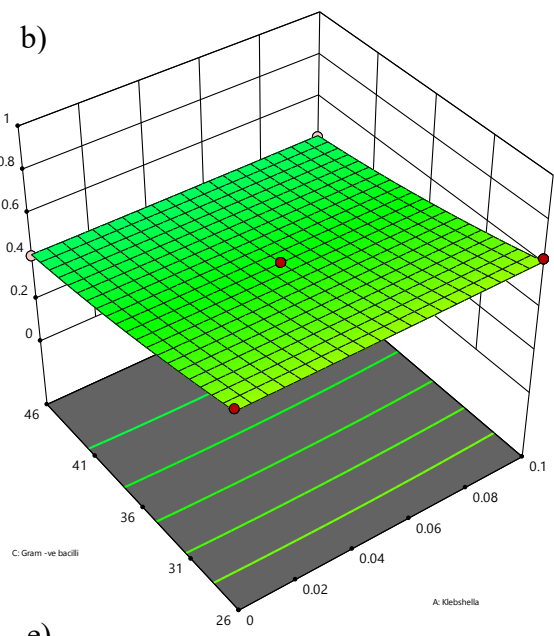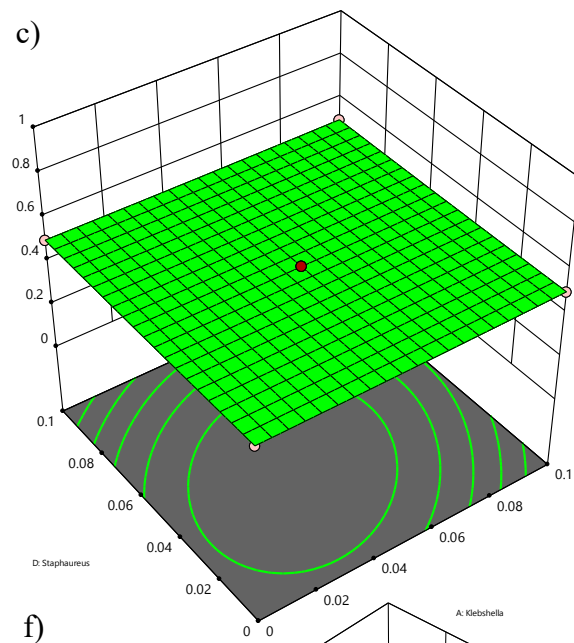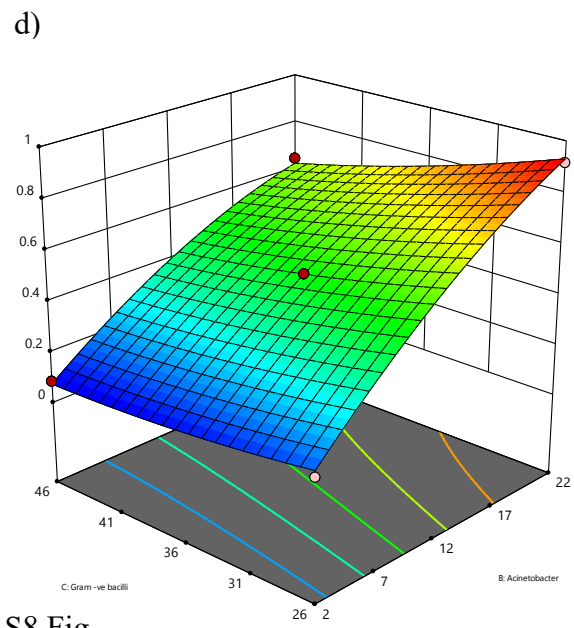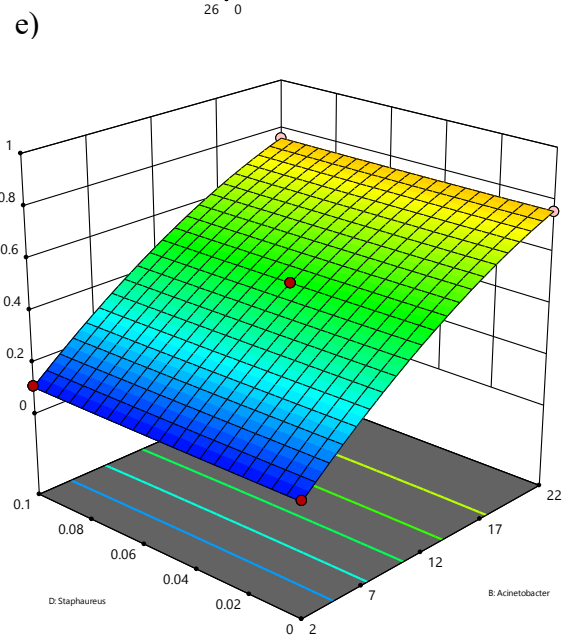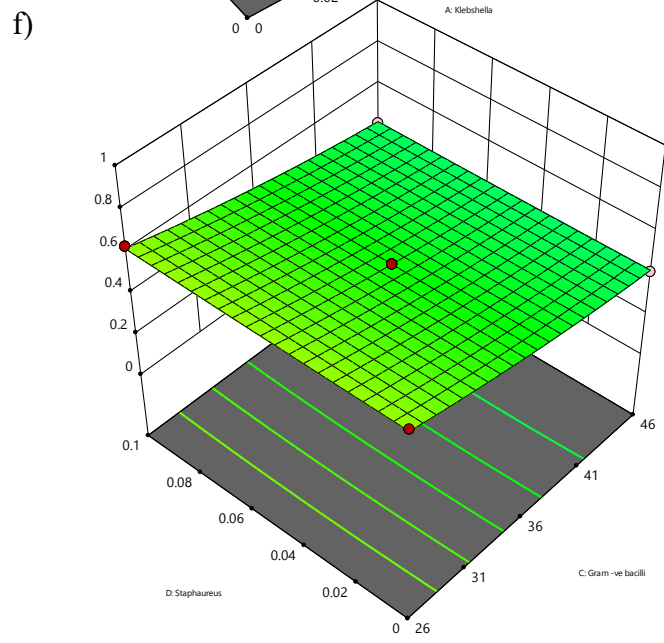

S8 Fig.

Supplement: S8 Fig — Response surface plot for antimicrobial activity in stem of Artemsia absenthia in response to ethanol between a) Klebsiella and Acenitobacter b) Klebsiella and Gram negative Bacilli c) Klebsiella and S. aureus d) Gram negative Bacilli and Acenitobacter e) Acenitobacter and S. aureus f) Gram negative Bacilli and S. aureus. (PDF) [file pone.0284244.s008.pdf]

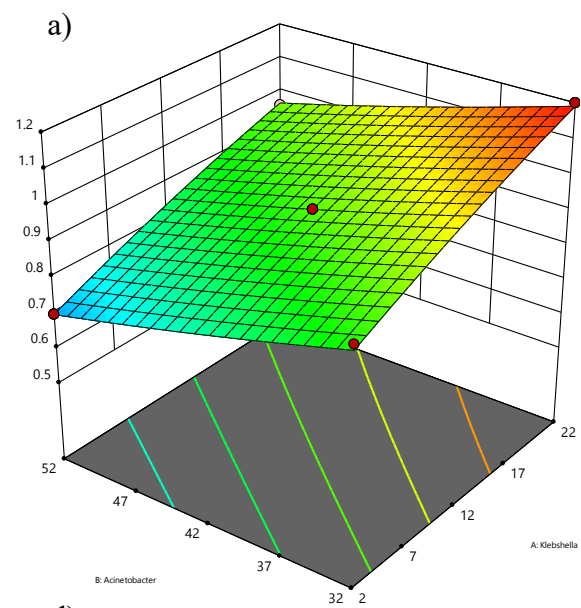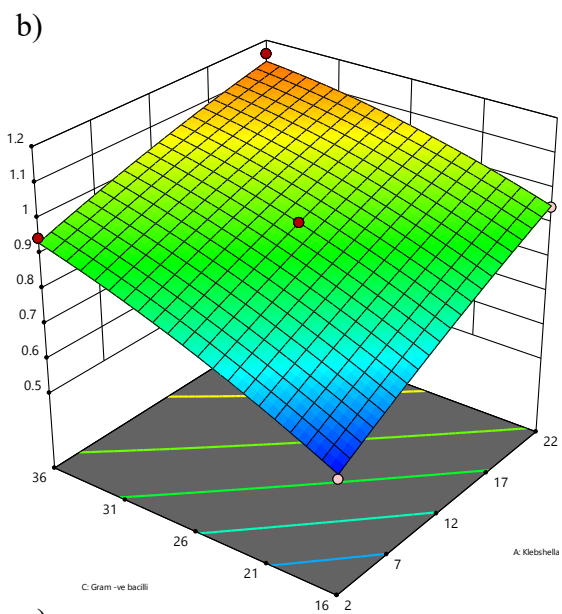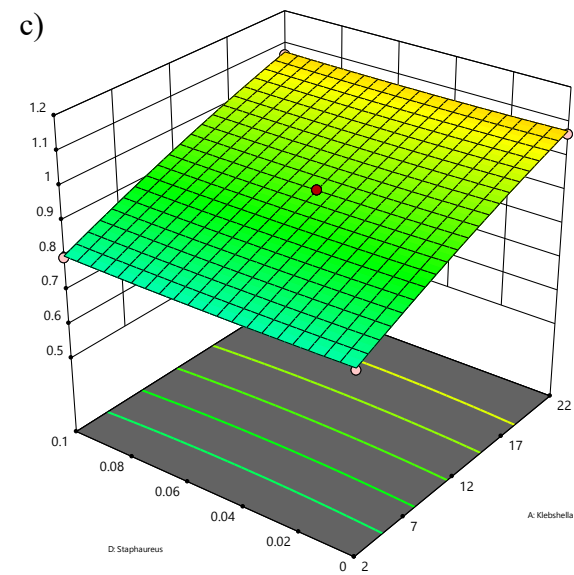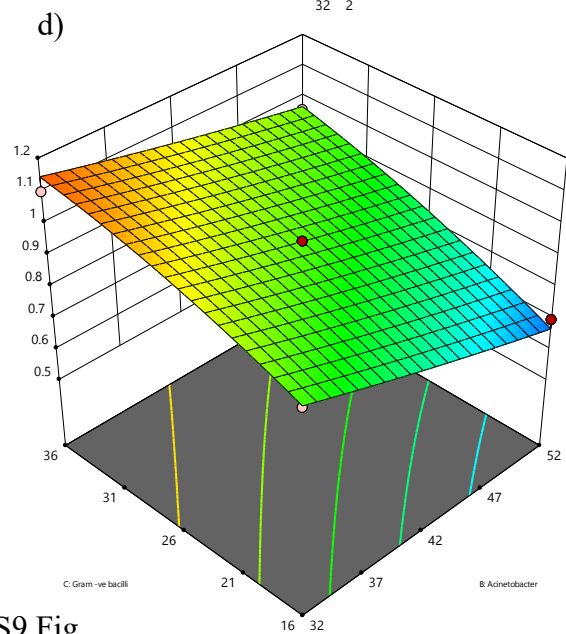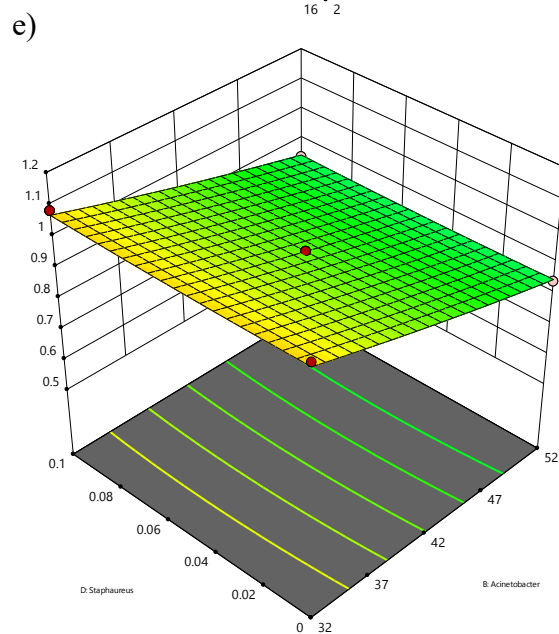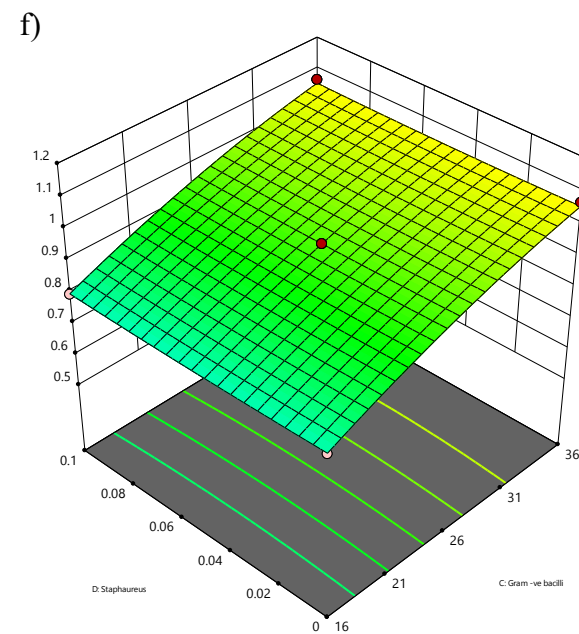

S9 Fig.

Supplement: S9 Fig — Response surface plot for antimicrobial activity in stem of Artemsia absenthia in response to acetone between a) Klebsiella and Acenitobacter b) Klebsiella and Gram negative Bacilli c) Klebsiella and S. aureus d) Gram negative Bacilli and Acenitobacter e) Acenitobacter and S. aureus f) Gram negative Bacilli and S. aureus. (PDF) [file pone.0284244.s009.pdf]

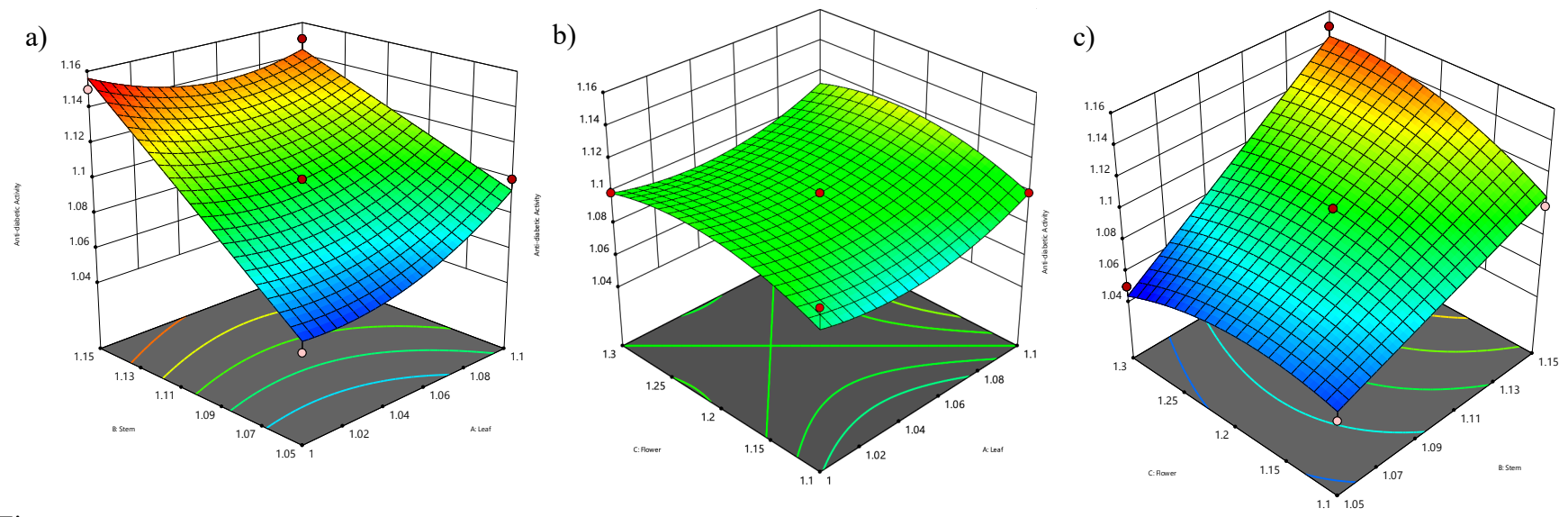

S10 Fig.

Supplement: S10 Fig — Response surface plot for antidiabetic activity of Artemsia absenthia in response to methanol between a) leaf and stem b) leaf and flower and c) stem and flower. (PDF) [file pone.0284244.s010.pdf]

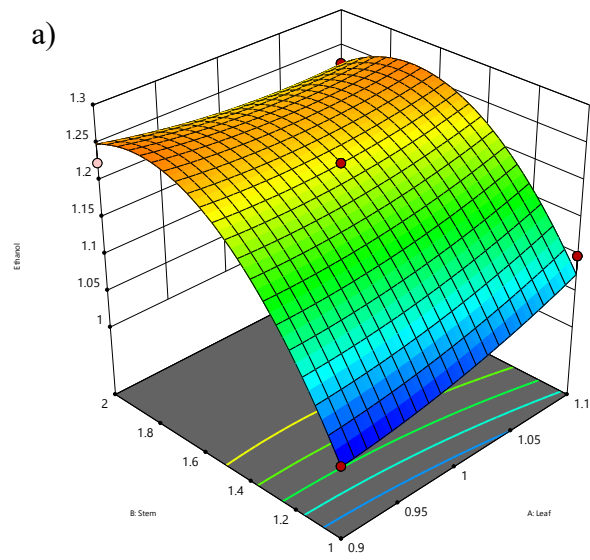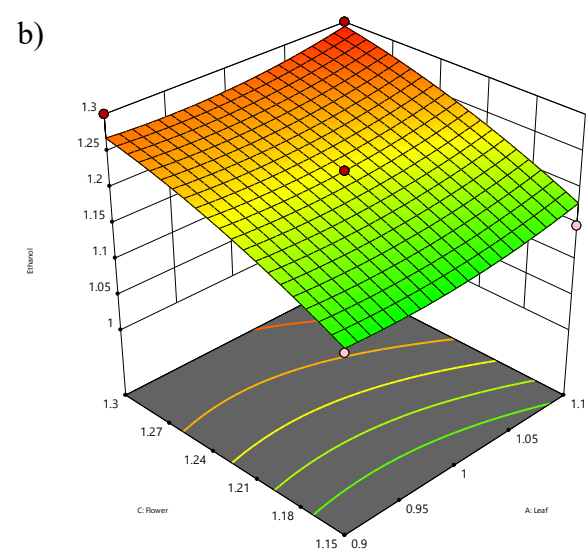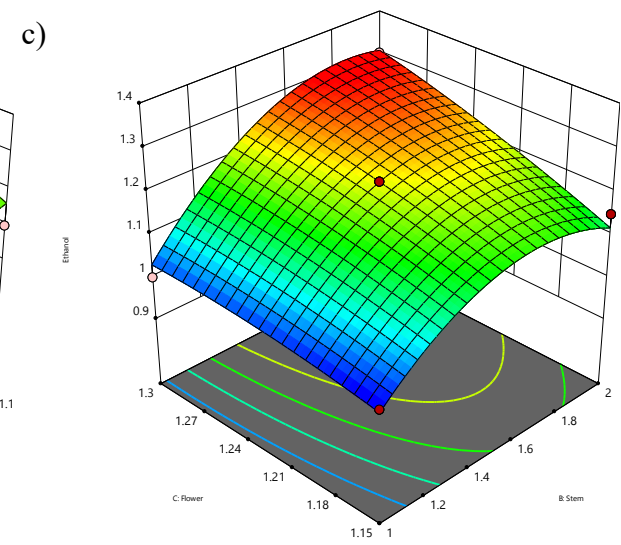

S11 Fig.

Supplement: S11 Fig — Response surface plot for antidiabetic activity of Artemsia absenthia in response to ethanol between a) leaf and stem b) leaf and flower and c) stem and flower. (PDF) [file pone.0284244.s011.pdf]

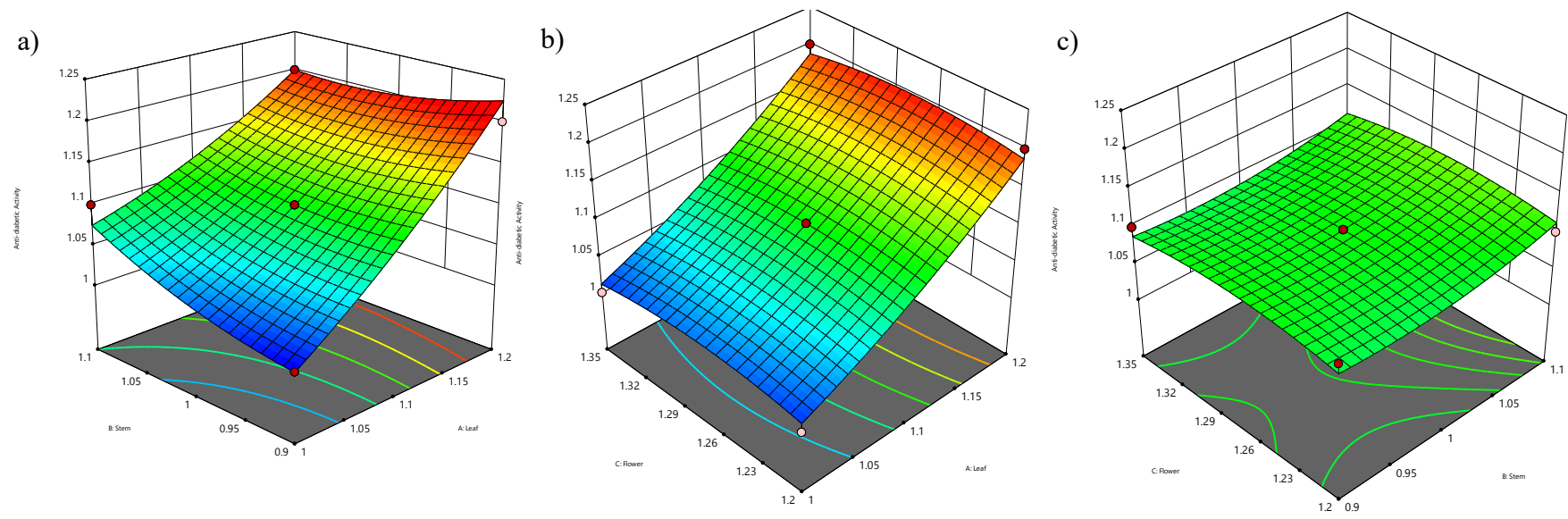

S12 Fig.

Supplement: S12 Fig — Response surface plot for antidiabetic activity of Artemsia absenthia in response to acetone between a) leaf and stem b) leaf and flower and c) stem and flower. (PDF) [file pone.0284244.s012.pdf]
