# Supplementary material for: Pharmacological activities of Artemisia absinthium and control of hepatic cancer by expression regulation of TGFβ1 and MYC genes
Source: PLoS One. 2023 Apr 13;18(4):e0284244. doi: 10.1371/journal.pone.0284244 (PMC10101520; doi:10.1371/journal.pone.0284244)
Supplement: S1 Table — (DOCX) [file pone.0284244.s013.docx]

Table S1:

| **Source** | **df** | **Sum of Squares** | **Mean Square** | **F–value** | **p–value** |
| --- | --- | --- | --- | --- | --- |
| **Model** | 14 | 0.7049 | 0.0503 | 3091.17 | < 0.0001 |
| A–Klebsiella | 1 | 0.0004 | 0.0004 | 25.39 | 0.0002 |
| B–Acinetobacter | 1 | 0.4376 | 0.4376 | 26867.10 | < 0.0001 |
| C–Gram –ve bacilli | 1 | 0.1653 | 0.1653 | 10147.34 | < 0.0001 |
| D–S. aureus | 1 | 0.0886 | 0.0886 | 5438.66 | < 0.0001 |
| AB | 1 | 1.448E–06 | 1.448E–06 | 0.0889 | 0.7699 |
| AC | 1 | 0.0000 | 0.0000 | 0.8482 | 0.3726 |
| AD | 1 | 2.339E–07 | 2.339E–07 | 0.0144 | 0.9063 |
| BC | 1 | 0.0005 | 0.0005 | 32.42 | < 0.0001 |
| BD | 1 | 0.0029 | 0.0029 | 176.77 | < 0.0001 |
| CD | 1 | 0.0001 | 0.0001 | 5.12 | 0.0400 |
| A² | 1 | 7.742E–07 | 7.742E–07 | 0.0475 | 0.8306 |
| B² | 1 | 0.0056 | 0.0056 | 343.40 | < 0.0001 |
| C² | 1 | 0.0021 | 0.0021 | 128.75 | < 0.0001 |
| D² | 1 | 0.0002 | 0.0002 | 13.99 | 0.0022 |
| **Residual** | 14 | 0.0002 | 0.0000 |  |  |
| Lack of Fit | 10 | 0.0002 | 0.0000 |  |  |
| Pure Error | 4 | 0.0000 | 0.0000 |  |  |
| **Cor Total** | 28 | 0.7051 |  |  |  |

R^2^ = 0.99
