# Supplementary material for: Pharmacological activities of Artemisia absinthium and control of hepatic cancer by expression regulation of TGFβ1 and MYC genes
Source: PLoS One. 2023 Apr 13;18(4):e0284244. doi: 10.1371/journal.pone.0284244 (PMC10101520; doi:10.1371/journal.pone.0284244)
Supplement: S2 Table — (DOCX) [file pone.0284244.s014.docx]

Table S2:

| **Source** | **Sum of Squares** | **df** | **Mean Square** | **F-value** | **p-value** |
| --- | --- | --- | --- | --- | --- |
| **Model** | 0.2485 | 14 | 0.0177 | 9.66 | < 0.0001 |
| A-Klebsiella | 0.0002 | 1 | 0.0002 | 0.1096 | 0.7455 |
| B-Acinetobacter | 0.0429 | 1 | 0.0429 | 23.38 | 0.0003 |
| C-Gram -ve bacilli | 0.0557 | 1 | 0.0557 | 30.32 | < 0.0001 |
| D-S. aureus | 0.0557 | 1 | 0.0557 | 30.32 | < 0.0001 |
| AB | 3.645E-06 | 1 | 3.645E-06 | 0.0020 | 0.9651 |
| AC | 4.783E-07 | 1 | 4.783E-07 | 0.0003 | 0.9874 |
| AD | 4.783E-07 | 1 | 4.783E-07 | 0.0003 | 0.9874 |
| BC | 0.0183 | 1 | 0.0183 | 9.97 | 0.0070 |
| BD | 0.0183 | 1 | 0.0183 | 9.97 | 0.0070 |
| CD | 6.102E-06 | 1 | 6.102E-06 | 0.0033 | 0.9549 |
| A² | 0.0001 | 1 | 0.0001 | 0.0626 | 0.8061 |
| B² | 0.0218 | 1 | 0.0218 | 11.89 | 0.0039 |
| C² | 0.0245 | 1 | 0.0245 | 13.32 | 0.0026 |
| D² | 0.0245 | 1 | 0.0245 | 13.32 | 0.0026 |
| **Residual** | 0.0257 | 14 | 0.0018 |  |  |
| Lack of Fit | 0.0257 | 10 | 0.0026 |  |  |
| Pure Error | 0.0000 | 4 | 0.0000 |  |  |
| **Cor Total** | 0.2742 | 28 |  |  |  |

R^2^ = 0.91
