# Supplementary material for: Pharmacological activities of Artemisia absinthium and control of hepatic cancer by expression regulation of TGFβ1 and MYC genes
Source: PLoS One. 2023 Apr 13;18(4):e0284244. doi: 10.1371/journal.pone.0284244 (PMC10101520; doi:10.1371/journal.pone.0284244)
Supplement: S3 Table — (DOCX) [file pone.0284244.s015.docx]

Table S3:

| **Source** | **Sum of Squares** | **df** | **Mean Square** | **F-value** | **p-value** |
| --- | --- | --- | --- | --- | --- |
| **Model** | 0.2071 | 14 | 0.0148 | 21.46 | < 0.0001 |
| A-Klebsiella | 0.0103 | 1 | 0.0103 | 14.98 | 0.0017 |
| B-Acinetobacter | 0.0514 | 1 | 0.0514 | 74.60 | < 0.0001 |
| C-Gram -ve bacilli | 0.0565 | 1 | 0.0565 | 81.91 | < 0.0001 |
| D-S. aureus | 0.0645 | 1 | 0.0645 | 93.64 | < 0.0001 |
| AB | 0.0082 | 1 | 0.0082 | 11.89 | 0.0039 |
| AC | 0.0004 | 1 | 0.0004 | 0.5457 | 0.4723 |
| AD | 0.0058 | 1 | 0.0058 | 8.37 | 0.0118 |
| BC | 3.914E-07 | 1 | 3.914E-07 | 0.0006 | 0.9813 |
| BD | 0.0011 | 1 | 0.0011 | 1.62 | 0.2232 |
| CD | 4.295E-06 | 1 | 4.295E-06 | 0.0062 | 0.9382 |
| A² | 0.0083 | 1 | 0.0083 | 12.06 | 0.0037 |
| B² | 0.0007 | 1 | 0.0007 | 0.9714 | 0.3410 |
| C² | 0.0000 | 1 | 0.0000 | 0.0182 | 0.8946 |
| D² | 0.0002 | 1 | 0.0002 | 0.2981 | 0.5937 |
| **Residual** | 0.0096 | 14 | 0.0007 |  |  |
| Lack of Fit | 0.0096 | 10 | 0.0010 |  |  |
| Pure Error | 0.0000 | 4 | 0.0000 |  |  |
| **Cor Total** | 0.2168 | 28 |  |  |  |

R^2^ = 0.95
