# Supplementary material for: Pharmacological activities of Artemisia absinthium and control of hepatic cancer by expression regulation of TGFβ1 and MYC genes
Source: PLoS One. 2023 Apr 13;18(4):e0284244. doi: 10.1371/journal.pone.0284244 (PMC10101520; doi:10.1371/journal.pone.0284244)
Supplement: S4 Table — (DOCX) [file pone.0284244.s016.docx]

Table S4:

| **Source** | **Sum of Squares** | **df** | **Mean Square** | **F-value** | **p-value** |
| --- | --- | --- | --- | --- | --- |
| **Model** | 0.5702 | 14 | 0.0407 | 94.15 | < 0.0001 |
| A-Klebsiella | 0.1856 | 1 | 0.1856 | 429.05 | < 0.0001 |
| B-Acinetobacter | 0.2324 | 1 | 0.2324 | 537.24 | < 0.0001 |
| C-Gram -ve bacilli | 0.1230 | 1 | 0.1230 | 284.27 | < 0.0001 |
| D-S. aureus | 0.0146 | 1 | 0.0146 | 33.73 | < 0.0001 |
| AB | 0.0044 | 1 | 0.0044 | 10.16 | 0.0066 |
| AC | 0.0000 | 1 | 0.0000 | 0.1115 | 0.7434 |
| AD | 0.0045 | 1 | 0.0045 | 10.51 | 0.0059 |
| BC | 0.0001 | 1 | 0.0001 | 0.1782 | 0.6794 |
| BD | 0.0000 | 1 | 0.0000 | 0.0398 | 0.8447 |
| CD | 0.0005 | 1 | 0.0005 | 1.14 | 0.3033 |
| A² | 0.0000 | 1 | 0.0000 | 0.0463 | 0.8327 |
| B² | 0.0029 | 1 | 0.0029 | 6.61 | 0.0222 |
| C² | 0.0004 | 1 | 0.0004 | 1.00 | 0.3342 |
| D² | 0.0008 | 1 | 0.0008 | 1.83 | 0.1978 |
| **Residual** | 0.0061 | 14 | 0.0004 |  |  |
| Lack of Fit | 0.0061 | 10 | 0.0006 |  |  |
| Pure Error | 0.0000 | 4 | 0.0000 |  |  |
| **Cor Total** | 0.5762 | 28 |  |  |  |

R2 = 0.98
