# Supplementary material for: Pharmacological activities of Artemisia absinthium and control of hepatic cancer by expression regulation of TGFβ1 and MYC genes
Source: PLoS One. 2023 Apr 13;18(4):e0284244. doi: 10.1371/journal.pone.0284244 (PMC10101520; doi:10.1371/journal.pone.0284244)
Supplement: S5 Table — (DOCX) [file pone.0284244.s017.docx]

Table S5:

| **Source** | **Sum of Squares** | **df** | **Mean Square** | **F-value** | **p-value** |
| --- | --- | --- | --- | --- | --- |
| **Model** | 0.2175 | 14 | 0.0155 | 38.39 | < 0.0001 |
| A-Klebsiella | 0.0031 | 1 | 0.0031 | 7.75 | 0.0146 |
| B-Acinetobacter | 0.0611 | 1 | 0.0611 | 150.97 | < 0.0001 |
| C-Gram -ve bacilli | 0.0646 | 1 | 0.0646 | 159.79 | < 0.0001 |
| D-S. aureus | 0.0824 | 1 | 0.0824 | 203.76 | < 0.0001 |
| AB | 0.0000 | 1 | 0.0000 | 0.0699 | 0.7953 |
| AC | 6.023E-09 | 1 | 6.023E-09 | 0.0000 | 0.9970 |
| AD | 6.023E-09 | 1 | 6.023E-09 | 0.0000 | 0.9970 |
| BC | 0.0032 | 1 | 0.0032 | 7.94 | 0.0137 |
| BD | 4.875E-08 | 1 | 4.875E-08 | 0.0001 | 0.9914 |
| CD | 0.0007 | 1 | 0.0007 | 1.85 | 0.1950 |
| A² | 0.0002 | 1 | 0.0002 | 0.4096 | 0.5325 |
| B² | 0.0001 | 1 | 0.0001 | 0.1291 | 0.7247 |
| C² | 0.0017 | 1 | 0.0017 | 4.25 | 0.0583 |
| D² | 0.0000 | 1 | 0.0000 | 0.0679 | 0.7982 |
| **Residual** | 0.0057 | 14 | 0.0004 |  |  |
| Lack of Fit | 0.0057 | 10 | 0.0006 |  |  |
| Pure Error | 0.0000 | 4 | 0.0000 |  |  |
| **Cor Total** | 0.2231 | 28 |  |  |  |

R^2^ = 0.97
