# Supplementary material for: Pharmacological activities of Artemisia absinthium and control of hepatic cancer by expression regulation of TGFβ1 and MYC genes
Source: PLoS One. 2023 Apr 13;18(4):e0284244. doi: 10.1371/journal.pone.0284244 (PMC10101520; doi:10.1371/journal.pone.0284244)
Supplement: S6 Table — (DOCX) [file pone.0284244.s018.docx]

Table S6:

| **Source** | **Sum of Squares** | **df** | **Mean Square** | **F-value** | **p-value** |
| --- | --- | --- | --- | --- | --- |
| **Model** | 0.1001 | 14 | 0.0072 | 37.73 | < 0.0001 |
| A-Klebsiella | 0.0493 | 1 | 0.0493 | 260.26 | < 0.0001 |
| B-Acinetobacter | 0.0228 | 1 | 0.0228 | 120.32 | < 0.0001 |
| C-Gram -ve bacilli | 0.0096 | 1 | 0.0096 | 50.54 | < 0.0001 |
| D-S. aureus | 1.893E-06 | 1 | 1.893E-06 | 0.0100 | 0.9218 |
| AB | 4.368E-06 | 1 | 4.368E-06 | 0.0230 | 0.8815 |
| AC | 0.0002 | 1 | 0.0002 | 1.12 | 0.3075 |
| AD | 1.607E-10 | 1 | 1.607E-10 | 8.481E-07 | 0.9993 |
| BC | 0.0105 | 1 | 0.0105 | 55.58 | < 0.0001 |
| BD | 8.569E-09 | 1 | 8.569E-09 | 0.0000 | 0.9947 |
| CD | 9.268E-10 | 1 | 9.268E-10 | 4.890E-06 | 0.9983 |
| A² | 0.0002 | 1 | 0.0002 | 1.17 | 0.2974 |
| B² | 0.0033 | 1 | 0.0033 | 17.33 | 0.0010 |
| C² | 0.0053 | 1 | 0.0053 | 28.19 | 0.0001 |
| D² | 8.909E-06 | 1 | 8.909E-06 | 0.0470 | 0.8315 |
| **Residual** | 0.0027 | 14 | 0.0002 |  |  |
| Lack of Fit | 0.0027 | 10 | 0.0003 |  |  |
| Pure Error | 0.0000 | 4 | 0.0000 |  |  |
| **Cor Total** | 0.1028 | 28 |  |  |  |

R^2^ = 0.97
