# Supplementary material for: Pharmacological activities of Artemisia absinthium and control of hepatic cancer by expression regulation of TGFβ1 and MYC genes
Source: PLoS One. 2023 Apr 13;18(4):e0284244. doi: 10.1371/journal.pone.0284244 (PMC10101520; doi:10.1371/journal.pone.0284244)
Supplement: S7 Table — (DOCX) [file pone.0284244.s019.docx]

Table S7:

| **Source** | **Sum of Squares** | **df** | **Mean Square** | **F-value** | **p-value** |
| --- | --- | --- | --- | --- | --- |
| **Model** | 0.5345 | 14 | 0.0382 | 737.46 | < 0.0001 |
| A-Klebsiella | 0.0194 | 1 | 0.0194 | 374.42 | < 0.0001 |
| B-Acinetobacter | 0.0194 | 1 | 0.0194 | 374.42 | < 0.0001 |
| C-Gram -ve bacilli | 0.3724 | 1 | 0.3724 | 7192.24 | < 0.0001 |
| D-S. aureus | 0.0194 | 1 | 0.0194 | 374.42 | < 0.0001 |
| AB | 0.0014 | 1 | 0.0014 | 27.68 | 0.0001 |
| AC | 0.0010 | 1 | 0.0010 | 20.16 | 0.0005 |
| AD | 0.0014 | 1 | 0.0014 | 27.68 | 0.0001 |
| BC | 0.0010 | 1 | 0.0010 | 20.16 | 0.0005 |
| BD | 0.0014 | 1 | 0.0014 | 27.68 | 0.0001 |
| CD | 0.0010 | 1 | 0.0010 | 20.16 | 0.0005 |
| A² | 0.0034 | 1 | 0.0034 | 65.10 | < 0.0001 |
| B² | 0.0034 | 1 | 0.0034 | 65.10 | < 0.0001 |
| C² | 0.0966 | 1 | 0.0966 | 1865.37 | < 0.0001 |
| D² | 0.0034 | 1 | 0.0034 | 65.10 | < 0.0001 |
| **Residual** | 0.0007 | 14 | 0.0001 |  |  |
| Lack of Fit | 0.0007 | 10 | 0.0001 |  |  |
| Pure Error | 0.0000 | 4 | 0.0000 |  |  |
| **Cor Total** | 0.5352 | 28 |  |  |  |

R^2^ = 0.99
