# Supplementary material for: Pharmacological activities of Artemisia absinthium and control of hepatic cancer by expression regulation of TGFβ1 and MYC genes
Source: PLoS One. 2023 Apr 13;18(4):e0284244. doi: 10.1371/journal.pone.0284244 (PMC10101520; doi:10.1371/journal.pone.0284244)
Supplement: S8 Table — (DOCX) [file pone.0284244.s020.docx]

Table S8:

| **Source** | **Sum of Squares** | **df** | **Mean Square** | **F-value** | **p-value** |
| --- | --- | --- | --- | --- | --- |
| **Model** | 1.46 | 14 | 0.1045 | 683.23 | < 0.0001 |
| A-Klebsiella | 3.846E-06 | 1 | 3.846E-06 | 0.0251 | 0.8763 |
| B-Acinetobacter | 1.29 | 1 | 1.29 | 8441.78 | < 0.0001 |
| C-Gram -ve bacilli | 0.1205 | 1 | 0.1205 | 787.75 | < 0.0001 |
| D-S. aureus | 3.846E-06 | 1 | 3.846E-06 | 0.0251 | 0.8763 |
| AB | 9.269E-07 | 1 | 9.269E-07 | 0.0061 | 0.9391 |
| AC | 5.004E-10 | 1 | 5.004E-10 | 3.270E-06 | 0.9986 |
| AD | 4.331E-06 | 1 | 4.331E-06 | 0.0283 | 0.8688 |
| BC | 0.0109 | 1 | 0.0109 | 71.09 | < 0.0001 |
| BD | 9.269E-07 | 1 | 9.269E-07 | 0.0061 | 0.9391 |
| CD | 5.004E-10 | 1 | 5.004E-10 | 3.270E-06 | 0.9986 |
| A² | 0.0000 | 1 | 0.0000 | 0.1508 | 0.7036 |
| B² | 0.0317 | 1 | 0.0317 | 207.01 | < 0.0001 |
| C² | 0.0027 | 1 | 0.0027 | 17.76 | 0.0009 |
| D² | 0.0000 | 1 | 0.0000 | 0.1508 | 0.7036 |
| **Residual** | 0.0021 | 14 | 0.0002 |  |  |
| Lack of Fit | 0.0021 | 10 | 0.0002 |  |  |
| Pure Error | 0.0000 | 4 | 0.0000 |  |  |
| **Cor Total** | 1.47 | 28 |  |  |  |

R^2^ = 0.99
