# Supplementary material for: Pharmacological activities of Artemisia absinthium and control of hepatic cancer by expression regulation of TGFβ1 and MYC genes
Source: PLoS One. 2023 Apr 13;18(4):e0284244. doi: 10.1371/journal.pone.0284244 (PMC10101520; doi:10.1371/journal.pone.0284244)
Supplement: S9 Table — (DOCX) [file pone.0284244.s021.docx]

Table S9:

| **Source** | **Sum of Squares** | **df** | **Mean Square** | **F-value** | **p-value** |
| --- | --- | --- | --- | --- | --- |
| **Model** | 0.5715 | 14 | 0.0408 | 108.77 | < 0.0001 |
| A-Klebsiella | 0.2187 | 1 | 0.2187 | 582.63 | < 0.0001 |
| B-Acinetobacter | 0.1468 | 1 | 0.1468 | 391.26 | < 0.0001 |
| C-Gram -ve bacilli | 0.1925 | 1 | 0.1925 | 512.96 | < 0.0001 |
| D-S. aureus | 4.244E-06 | 1 | 4.244E-06 | 0.0113 | 0.9168 |
| AB | 0.0000 | 1 | 0.0000 | 0.0302 | 0.8645 |
| AC | 0.0049 | 1 | 0.0049 | 13.04 | 0.0028 |
| AD | 4.546E-10 | 1 | 4.546E-10 | 1.211E-06 | 0.9991 |
| BC | 0.0022 | 1 | 0.0022 | 5.79 | 0.0305 |
| BD | 9.351E-08 | 1 | 9.351E-08 | 0.0002 | 0.9876 |
| CD | 4.546E-10 | 1 | 4.546E-10 | 1.211E-06 | 0.9991 |
| A² | 0.0011 | 1 | 0.0011 | 2.95 | 0.1081 |
| B² | 0.0003 | 1 | 0.0003 | 0.8340 | 0.3766 |
| C² | 0.0042 | 1 | 0.0042 | 11.28 | 0.0047 |
| D² | 0.0001 | 1 | 0.0001 | 0.3451 | 0.5663 |
| **Residual** | 0.0053 | 14 | 0.0004 |  |  |
| Lack of Fit | 0.0053 | 10 | 0.0005 |  |  |
| Pure Error | 0.0000 | 4 | 0.0000 |  |  |
| **Cor Total** | 0.5768 | 28 |  |  |  |

R^2^ = 0.99
