# Supplementary material for: Pharmacological activities of Artemisia absinthium and control of hepatic cancer by expression regulation of TGFβ1 and MYC genes
Source: PLoS One. 2023 Apr 13;18(4):e0284244. doi: 10.1371/journal.pone.0284244 (PMC10101520; doi:10.1371/journal.pone.0284244)
Supplement: S10 Table — (DOCX) [file pone.0284244.s022.docx]

Table S10:

| Runs | Klebsiella | Acinetobacter | Gram -ve bacilli | S. aureus | Anti-microbial activity | |
| --- | --- | --- | --- | --- | --- | --- |
|  |  |  |  |  | Actual | Predicted |
| **1** | **1** | **20** | **30** | **5.5** | **0.902655** | **0.9007** |
| **2** | **0.5** | **20** | **30** | **10** | **0.991736** | **0.9926** |
| 3 | 0 | 10 | 40 | 5.5 | 0.558559 | 0.558 |
| **4** | **0.5** | **30** | **30** | **5.5** | **1.075758** | **1.08** |
| 5 | 0.5 | 20 | 40 | 5.5 | 0.772727 | 0.7727 |
| 6 | 0 | 20 | 50 | 5.5 | 0.675497 | 0.6777 |
| **7** | **1** | **30** | **40** | **5.5** | **0.928105** | **0.9282** |
| 8 | 0.5 | 10 | 30 | 5.5 | 0.673913 | 0.6762 |
| 9 | 0.5 | 20 | 50 | 10 | 0.745342 | 0.7488 |
| 10 | 0.5 | 10 | 50 | 5.5 | 0.469697 | 0.4645 |
| 11 | 0.5 | 20 | 40 | 5.5 | 0.772727 | 0.7727 |
| 12 | 0.5 | 30 | 40 | 10 | 0.993789 | 0.9875 |
| 13 | 0.5 | 10 | 40 | 10 | 0.661157 | 0.6592 |
| 14 | 0.5 | 30 | 50 | 5.5 | 0.825581 | 0.8235 |
| **15** | **0** | **20** | **30** | **5.5** | **0.918919** | **0.9161** |
| 16 | 0 | 20 | 40 | 1 | 0.688525 | 0.6869 |
| 17 | 0.5 | 30 | 40 | 1 | 0.867133 | 0.8693 |
| 18 | 0.5 | 20 | 40 | 5.5 | 0.772727 | 0.7727 |
| 19 | 0.5 | 20 | 40 | 5.5 | 0.772727 | 0.7727 |
| 20 | 1 | 20 | 40 | 1 | 0.677419 | 0.6756 |
| 21 | 0 | 20 | 40 | 10 | 0.857143 | 0.8592 |
| 22 | 0.5 | 20 | 30 | 1 | 0.815534 | 0.8117 |
| 23 | 1 | 10 | 40 | 5.5 | 0.548673 | 0.5475 |
| 24 | 1 | 20 | 50 | 5.5 | 0.666667 | 0.6697 |
| **25** | **0** | **30** | **40** | **5.5** | **0.940397** | **0.9411** |
| 26 | 0.5 | 20 | 50 | 1 | 0.587413 | 0.5861 |
| 27 | 1 | 20 | 40 | 10 | 0.84507 | 0.847 |
| 28 | 0.5 | 10 | 40 | 1 | 0.427184 | 0.4337 |
| 29 | 0.5 | 20 | 40 | 5.5 | 0.772727 | 0.7727 |
