# Supplementary material for: Pharmacological activities of Artemisia absinthium and control of hepatic cancer by expression regulation of TGFβ1 and MYC genes
Source: PLoS One. 2023 Apr 13;18(4):e0284244. doi: 10.1371/journal.pone.0284244 (PMC10101520; doi:10.1371/journal.pone.0284244)
Supplement: S11 Table — (DOCX) [file pone.0284244.s023.docx]

Table S11:

| Runs | Klebsiella | Acinetobacter | Gram -ve bacilli | S. aureus | Anti-microbial activity | |
| --- | --- | --- | --- | --- | --- | --- |
|  |  |  |  |  | Actual | Predicted |
| 1 | 0.25 | 18 | 22 | 32 | 1.107266 | 1.16 |
| **2** | **0** | **18** | **22** | **22** | **1.290323** | **1.23** |
| 3 | 0.5 | 28 | 22 | 12 | 1.088 | 1.09 |
| **4** | **0.25** | **28** | **32** | **32** | **1.300813** | **1.23** |
| **5** | **0.25** | **28** | **22** | **22** | **1.217993** | **1.22** |
| **6** | **0.5** | **28** | **22** | **32** | **1.212121** | **1.23** |
| 7 | 0.25 | 38 | 12 | 22 | 0.941176 | 0.903 |
| 8 | 0.25 | 38 | 32 | 22 | 1.170732 | 1.17 |
| 9 | 0.25 | 28 | 12 | 32 | 1.107266 | 1.09 |
| 10 | 0.25 | 18 | 12 | 22 | 1.148325 | 1.16 |
| **11** | **0** | **28** | **22** | **32** | **1.219512** | **1.23** |
| 12 | 0 | 28 | 12 | 22 | 1.096774 | 1.1 |
| 13 | 0.25 | 28 | 32 | 12 | 1.107266 | 1.09 |
| 14 | 0.25 | 28 | 22 | 22 | 1.217993 | 1.22 |
| 15 | 0.25 | 38 | 22 | 32 | 1.170732 | 1.17 |
| 16 | 0.25 | 28 | 22 | 22 | 1.217993 | 1.22 |
| 17 | 0.5 | 18 | 22 | 22 | 1.28 | 1.22 |
| 18 | 0.25 | 18 | 22 | 12 | 1.148325 | 1.16 |
| 19 | 0 | 38 | 22 | 22 | 1.073171 | 1.11 |
| **20** | **0.5** | **28** | **32** | **22** | **1.212121** | **1.23** |
| **21** | **0.25** | **28** | **22** | **22** | **1.217993** | **1.22** |
| 22 | 0 | 28 | 22 | 12 | 1.096774 | 1.1 |
| 23 | 0.25 | 28 | 12 | 12 | 0.91866 | 0.9601 |
| 24 | 0.25 | 38 | 22 | 12 | 0.941176 | 0.903 |
| 25 | 0.5 | 28 | 12 | 22 | 1.088 | 1.09 |
| **26** | **0.25** | **28** | **22** | **22** | **1.217993** | **1.22** |
| 27 | 0.5 | 38 | 22 | 22 | 1.066667 | 1.1 |
| **28** | **0** | **28** | **32** | **22** | **1.219512** | **1.23** |
| 29 | 0.25 | 18 | 32 | 22 | 1.107266 | 1.16 |
