# Supplementary material for: Pharmacological activities of Artemisia absinthium and control of hepatic cancer by expression regulation of TGFβ1 and MYC genes
Source: PLoS One. 2023 Apr 13;18(4):e0284244. doi: 10.1371/journal.pone.0284244 (PMC10101520; doi:10.1371/journal.pone.0284244)
Supplement: S12 Table — (DOCX) [file pone.0284244.s024.docx]

Table S12:

| Runs | Klebsiella | Acinetobacter | Gram -ve bacilli | Staphauerus | Anti-microbial activity | |
| --- | --- | --- | --- | --- | --- | --- |
|  |  |  |  |  | Actual | Predicted |
| 1 | 29 | 27 | 64 | 28 | 0.77027 | 0.8056 |
| **2** | **19** | **37** | **54** | **18** | **0.875** | **0.884** |
| **3** | **19** | **17** | **44** | **28** | **0.87037** | **0.871** |
| 4 | 19 | 27 | 54 | 28 | 0.859375 | 0.8594 |
| **5** | **19** | **17** | **54** | **38** | **0.890625** | **0.8998** |
| **6** | **29** | **17** | **54** | **28** | **0.890625** | **0.8558** |
| 7 | 9 | 27 | 54 | 38 | **1.015625** | 1.04 |
| 8 | 29 | 27 | 44 | 28 | **0.890625** | 0.9233 |
| 9 | 9 | 17 | 54 | 28 | 0.833333 | 0.8239 |
| 10 | 19 | 17 | 54 | 18 | 0.685185 | 0.7196 |
| **11** | **19** | **27** | **64** | **38** | **0.878378** | **0.8673** |
| **12** | **9** | **27** | **44** | **28** | **1.018519** | **1** |
| **13** | **29** | **27** | **54** | **38** | **0.905405** | **0.9068** |
| **14** | **19** | **27** | **44** | **38** | **1.015625** | **1.01** |
| 15 | 9 | 27 | 64 | 28 | 0.859375 | 0.8448 |
| 16 | 19 | 27 | 54 | 28 | 0.859375 | 0.8594 |
| 17 | 19 | 17 | 64 | 28 | 0.734375 | 0.7344 |
| **18** | **29** | **37** | **54** | **28** | **0.891892** | **0.8962** |
| **19** | **29** | **27** | **54** | **18** | **0.875** | **0.8361** |
| 20 | 9 | 37 | 54 | 28 | **1.015625** | 1.05 |
| 21 | 9 | 27 | 54 | 18 | 0.833333 | 0.8188 |
| 22 | 19 | 27 | 64 | 18 | 0.71875 | 0.7227 |
| 23 | 19 | 27 | 44 | 18 | 0.851852 | 0.8578 |
| 24 | 19 | 27 | 54 | 28 | 0.859375 | 0.8594 |
| 25 | 19 | 27 | 54 | 28 | 0.859375 | 0.8594 |
| **26** | **19** | **37** | **54** | **38** | **1.013514** | **0.9972** |
| **27** | **19** | **37** | **64** | **28** | **0.878378** | **0.8647** |
| **28** | **19** | **37** | **44** | **28** | **1.015625** | **1** |
| 29 | 19 | 27 | 54 | 28 | 0.859375 | 0.8594 |
