# Supplementary material for: Pharmacological activities of Artemisia absinthium and control of hepatic cancer by expression regulation of TGFβ1 and MYC genes
Source: PLoS One. 2023 Apr 13;18(4):e0284244. doi: 10.1371/journal.pone.0284244 (PMC10101520; doi:10.1371/journal.pone.0284244)
Supplement: S13 Table — (DOCX) [file pone.0284244.s025.docx]

Table S13:

| Runs | Klebsiella | Acinetobacter | Gram -ve bacilli | S. aureus | Anti-microbial activity | |
| --- | --- | --- | --- | --- | --- | --- |
|  |  |  |  |  | Actual | Predicted |
| **1** | **25** | **21** | **33** | **5.5** | **1.088757** | **1.09** |
| 2 | 15 | 11 | 43 | 1 | 0.742857 | 0.7357 |
| 3 | 15 | 21 | 43 | 5.5 | 0.852071 | 0.8521 |
| 4 | 15 | 11 | 43 | 10 | 0.658228 | 0.6702 |
| 5 | 15 | 21 | 43 | 5.5 | 0.852071 | 0.8521 |
| 6 | 15 | 31 | 43 | 10 | 0.929293 | 0.9443 |
| 7 | 5 | 21 | 43 | 1 | 0.742857 | 0.7381 |
| 8 | 15 | 21 | 43 | 5.5 | 0.852071 | 0.8521 |
| **9** | **15** | **31** | **43** | **1** | **1.022222** | **1.02** |
| 10 | 15 | 31 | 53 | 5.5 | 0.880383 | 0.8728 |
| 11 | 15 | 11 | 33 | 5.5 | 0.806202 | 0.7969 |
| **12** | **25** | **21** | **43** | **1** | **1.022222** | **1.05** |
| 13 | 5 | 21 | 33 | 5.5 | 0.821705 | 0.8319 |
| 14 | 5 | 21 | 53 | 5.5 | 0.627219 | 0.6364 |
| 15 | 5 | 11 | 43 | 5.5 | 0.511628 | 0.5326 |
| 16 | 25 | 21 | 53 | 5.5 | 0.880383 | 0.8781 |
| **17** | **15** | **31** | **33** | **5.5** | **1.088757** | **1.08** |
| 18 | 15 | 21 | 53 | 10 | 0.727273 | 0.7463 |
| 19 | 5 | 21 | 43 | 10 | 0.78481 | 0.7358 |
| 20 | 5 | 31 | 43 | 5.5 | 0.863905 | 0.8773 |
| 21 | 15 | 21 | 33 | 10 | 0.911392 | 0.9265 |
| **22** | **15** | **21** | **33** | **1** | **1.028571** | **1.02** |
| 23 | 25 | 21 | 43 | 10 | 0.929293 | 0.9171 |
| 24 | 15 | 21 | 43 | 5.5 | 0.852071 | 0.8521 |
| 25 | 25 | 11 | 43 | 5.5 | 0.852071 | 0.8477 |
| 26 | 15 | 21 | 53 | 1 | 0.8 | 0.7938 |
| **27** | **25** | **31** | **43** | **5.5** | **1.07177** | **1.06** |
| 28 | 15 | 11 | 53 | 5.5 | 0.615385 | 0.6032 |
| 29 | 15 | 21 | 43 | 5.5 | 0.852071 | 0.8521 |
