# Supplementary material for: Pharmacological activities of Artemisia absinthium and control of hepatic cancer by expression regulation of TGFβ1 and MYC genes
Source: PLoS One. 2023 Apr 13;18(4):e0284244. doi: 10.1371/journal.pone.0284244 (PMC10101520; doi:10.1371/journal.pone.0284244)
Supplement: S14 Table — (DOCX) [file pone.0284244.s026.docx]

Table S14:

| Runs | Klebsiella | Acinetobacter | Gram -ve bacilli | S. aureus | Antimicrobial activity | |
| --- | --- | --- | --- | --- | --- | --- |
|  |  |  |  |  | Actual | Predicted |
| 1 | 4 | 58 | 35 | 26 | 0.99187 | 0.9967 |
| **2** | **2** | **58** | **45** | **26** | **1.083969** | **1.09** |
| 3 | 4 | 58 | 45 | 16 | 0.99187 | 0.9919 |
| 4 | 4 | 58 | 35 | 6 | 0.796117 | 0.8036 |
| **5** | **4** | **48** | **55** | **16** | **1.04065** | **1.09** |
| **6** | **4** | **58** | **55** | **26** | **1.132867** | **1.12** |
| **7** | **6** | **58** | **45** | **26** | **1.051852** | **1.06** |
| 8 | 2 | 68 | 45 | 16 | 0.931298 | 0.9362 |
| **9** | **6** | **48** | **45** | **16** | **1.06087** | **1.05** |
| 10 | 4 | 58 | 45 | 16 | 0.99187 | 0.9919 |
| **11** | **6** | **58** | **55** | **16** | **1.051852** | **1.04** |
| 12 | 4 | 68 | 45 | 6 | 0.829268 | 0.8329 |
| 13 | 4 | 48 | 45 | 6 | 0.990291 | 0.9753 |
| 14 | 4 | 68 | 35 | 16 | 0.829268 | 0.7997 |
| 15 | 2 | 58 | 35 | 16 | 0.918919 | 0.9234 |
| 16 | 6 | 68 | 45 | 16 | 0.903704 | 0.9092 |
| 17 | 6 | 58 | 45 | 6 | 0.886957 | 0.8959 |
| 18 | 4 | 58 | 45 | 16 | 0.99187 | 0.9919 |
| 19 | 6 | 58 | 35 | 16 | 0.886957 | 0.8911 |
| **20** | **2** | **48** | **45** | **16** | **1.099099** | **1.08** |
| 21 | 4 | 68 | 45 | 26 | 0.993007 | 0.9984 |
| 22 | 4 | 58 | 45 | 16 | 0.99187 | 0.9919 |
| 23 | 2 | 58 | 45 | 6 | 0.918919 | 0.9281 |
| 24 | 4 | 58 | 45 | 16 | 0.99187 | 0.9919 |
| 25 | 4 | 48 | 35 | 16 | 0.990291 | 0.999 |
| **26** | **4** | **48** | **45** | **26** | **1.154472** | **1.14** |
| 27 | 4 | 58 | 55 | 6 | 0.99187 | 0.9777 |
| 28 | 4 | 68 | 55 | 16 | 0.993007 | 1 |
| **29** | **2** | **58** | **55** | **16** | **1.083969** | **1.07** |
