# Supplementary material for: Pharmacological activities of Artemisia absinthium and control of hepatic cancer by expression regulation of TGFβ1 and MYC genes
Source: PLoS One. 2023 Apr 13;18(4):e0284244. doi: 10.1371/journal.pone.0284244 (PMC10101520; doi:10.1371/journal.pone.0284244)
Supplement: S15 Table — (DOCX) [file pone.0284244.s027.docx]

Table S15:

| Runs | Klebsiella | Acinetobacter | Gram -ve bacilli | S. aureus | Antimicrobial activity | |
| --- | --- | --- | --- | --- | --- | --- |
|  |  |  |  |  | Actual | Predicted |
| 1 | 14 | 61 | 55 | 0.1 | 1.060723 | 1.06 |
| 2 | 24 | 71 | 55 | 0 | 1.053333 | 1.06 |
| 3 | 24 | 51 | 65 | 0.05 | 1.071046 | 1.1 |
| **4** | **24** | **71** | **65** | **0.05** | **1.112152** | **1.11** |
| **5** | **24** | **61** | **55** | **0.05** | **1.128169** | **1.13** |
| 6 | 24 | 71 | 45 | 0.05 | 0.985362 | 0.9538 |
| **7** | **24** | **61** | **55** | **0.05** | **1.128169** | **1.13** |
| **8** | **24** | **61** | **65** | **0** | **1.133333** | **1.13** |
| **9** | **24** | **61** | **65** | **0.1** | **1.132578** | **1.13** |
| 10 | 14 | 61 | 65 | 0.05 | 1.071046 | 1.07 |
| 11 | 14 | 61 | 55 | 0 | 1.061538 | 1.06 |
| **12** | **24** | **61** | **55** | **0.05** | **1.128169** | **1.13** |
| **13** | **34** | **71** | **55** | **0.05** | **1.112152** | **1.12** |
| **14** | **34** | **61** | **55** | **0.1** | **1.185876** | **1.18** |
| **15** | **24** | **51** | **55** | **0** | **1.153846** | **1.15** |
| 16 | 14 | 51 | 55 | 0.05 | 1.082882 | 1.08 |
| **17** | **34** | **61** | **45** | **0.05** | **1.128169** | **1.14** |
| 18 | 14 | 61 | 45 | 0.05 | 0.982924 | 0.994 |
| **19** | **24** | **51** | **55** | **0.1** | **1.152959** | **1.15** |
| 20 | 14 | 71 | 55 | 0.05 | 0.985362 | 0.9932 |
| **21** | **34** | **61** | **65** | **0.05** | **1.187129** | **1.18** |
| **22** | **34** | **61** | **55** | **0** | **1.186667** | **1.19** |
| 23 | 24 | 71 | 55 | 0.1 | 1.052632 | 1.06 |
| **24** | **24** | **61** | **55** | **0.05** | **1.128169** | **1.13** |
| **25** | **24** | **61** | **55** | **0.05** | **1.128169** | **1.13** |
| 26 | 24 | 61 | 45 | 0.1 | 1.060723 | 1.07 |
| **27** | **24** | **51** | **45** | **0.05** | **1.149521** | **1.14** |
| 28 | 24 | 61 | 45 | 0 | 1.061538 | 1.07 |
| **29** | **34** | **51** | **55** | **0.05** | **1.213852** | **1.21** |
