# Supplementary material for: Pharmacological activities of Artemisia absinthium and control of hepatic cancer by expression regulation of TGFβ1 and MYC genes
Source: PLoS One. 2023 Apr 13;18(4):e0284244. doi: 10.1371/journal.pone.0284244 (PMC10101520; doi:10.1371/journal.pone.0284244)
Supplement: S16 Table — (DOCX) [file pone.0284244.s028.docx]

Table S16:

| Runs | Klebsiella | Acinetobacter | Gram -ve bacilli | S. aureus | Antimicrobial activity | |
| --- | --- | --- | --- | --- | --- | --- |
|  |  |  |  |  | Actual | Predicted |
| 1 | 0 | 0.05 | 1.4 | 0.1 | 0.193548 | 0.1935 |
| 2 | 0.05 | 0 | 1.4 | 0 | 0.068966 | 0.0753 |
| 3 | 0.1 | 0.05 | 1.4 | 0.1 | 0.242424 | 0.2361 |
| 4 | 0.05 | 0.1 | 2.4 | 0.05 | 0.115385 | 0.1217 |
| 5 | 0.1 | 0.1 | 1.4 | 0.05 | 0.242424 | 0.2361 |
| 6 | 0.05 | 0.05 | 1.4 | 0.05 | 0.129032 | 0.129 |
| 7 | 0.05 | 0.1 | 1.4 | 0 | 0.193548 | 0.1935 |
| **8** | **0.05** | **0.1** | **0.4** | **0.05** | **0.5** | **0.5063** |
| 9 | 0.05 | 0.1 | 1.4 | 0.1 | 0.242424 | 0.2361 |
| 10 | 0.05 | 0.05 | 1.4 | 0.05 | 0.129032 | 0.129 |
| **11** | **0.05** | **0.05** | **0.4** | **0** | **0.4** | **0.3937** |
| 12 | 0.05 | 0.05 | 2.4 | 0 | 0.08 | 0.0737 |
| 13 | 0.05 | 0 | 1.4 | 0.1 | 0.193548 | 0.1935 |
| 14 | 0.05 | 0 | 2.4 | 0.05 | 0.08 | 0.0737 |
| 15 | 0.1 | 0 | 1.4 | 0.05 | 0.193548 | 0.1935 |
| 16 | 0 | 0.05 | 1.4 | 0 | 0.068966 | 0.0753 |
| **17** | **0.05** | **0** | **0.4** | **0.05** | **0.4** | **0.3937** |
| 18 | 0 | 0.05 | 2.4 | 0.05 | 0.08 | 0.0737 |
| 19 | 0.1 | 0.05 | 1.4 | 0 | 0.193548 | 0.1935 |
| 20 | 0 | 0 | 1.4 | 0.05 | 0.068966 | 0.0753 |
| **21** | **0** | **0.05** | **0.4** | **0.05** | **0.4** | **0.3937** |
| 22 | 0.1 | 0.05 | 2.4 | 0.05 | 0.115385 | 0.1217 |
| 23 | 0.05 | 0.05 | 1.4 | 0.05 | 0.129032 | 0.129 |
| 24 | 0.05 | 0.05 | 1.4 | 0.05 | 0.129032 | 0.129 |
| **25** | **0.1** | **0.05** | **0.4** | **0.05** | **0.5** | **0.5063** |
| 26 | 0.05 | 0.05 | 2.4 | 0.1 | 0.115385 | 0.1217 |
| 27 | 0 | 0.1 | 1.4 | 0.05 | 0.193548 | 0.1935 |
| **28** | **0.05** | **0.05** | **0.4** | **0.1** | **0.5** | **0.5063** |
| 29 | 0.05 | 0.05 | 1.4 | 0.05 | 0.129032 | 0.129 |
