# Supplementary material for: Pharmacological activities of Artemisia absinthium and control of hepatic cancer by expression regulation of TGFβ1 and MYC genes
Source: PLoS One. 2023 Apr 13;18(4):e0284244. doi: 10.1371/journal.pone.0284244 (PMC10101520; doi:10.1371/journal.pone.0284244)
Supplement: S17 Table — (DOCX) [file pone.0284244.s029.docx]

Table S17:

| Runs | Klebsiella | Acinetobacter | Gram -ve bacilli | S. aureus | Antimicrobial activity | |
| --- | --- | --- | --- | --- | --- | --- |
|  |  |  |  |  | Actual | Predicted |
| **1** | **0.1** | **12** | **26** | **0.05** | **0.634338** | **0.6242** |
| 2 | 0.1 | 2 | 36 | 0.05 | 0.110092 | 0.106 |
| **3** | **0** | **12** | **26** | **0.05** | **0.633377** | **0.6231** |
| 4 | 0 | 12 | 36 | 0 | 0.5 | 0.5026 |
| 5 | 0.05 | 2 | 46 | 0.05 | 0.085239 | 0.0754 |
| 6 | 0.1 | 12 | 36 | 0 | 0.503119 | 0.5059 |
| 7 | 0.05 | 12 | 36 | 0.05 | 0.50104 | 0.501 |
| 8 | 0.05 | 12 | 36 | 0.05 | 0.50104 | 0.501 |
| 9 | 0.05 | 2 | 36 | 0 | 0.107753 | 0.1039 |
| 10 | 0.05 | 2 | 26 | 0.05 | 0.145907 | 0.1716 |
| **11** | **0.05** | **22** | **36** | **0.1** | **0.760103** | **0.7612** |
| **12** | **0.05** | **22** | **46** | **0.05** | **0.647577** | **0.6273** |
| 13 | 0 | 2 | 36 | 0.05 | 0.107753 | 0.1039 |
| **14** | **0.05** | **12** | **26** | **0.1** | **0.634338** | **0.6242** |
| 15 | 0.05 | 2 | 36 | 0.1 | 0.110092 | 0.106 |
| 16 | 0.1 | 12 | 46 | 0.05 | 0.416165 | 0.4237 |
| 17 | 0.05 | 12 | 36 | 0.05 | 0.50104 | 0.501 |
| 18 | 0 | 22 | 36 | 0.05 | 0.75969 | 0.7611 |
| 19 | 0 | 12 | 36 | 0.1 | 0.503119 | 0.5059 |
| 20 | 0.05 | 12 | 36 | 0.05 | 0.50104 | 0.501 |
| 21 | 0.1 | 12 | 36 | 0.1 | 0.502075 | 0.5049 |
| 22 | 0 | 12 | 46 | 0.05 | 0.415159 | 0.4226 |
| 23 | 0.05 | 12 | 36 | 0.05 | 0.50104 | 0.501 |
| **24** | **0.05** | **22** | **36** | **0** | **0.75969** | **0.7611** |
| 25 | 0.05 | 12 | 26 | 0 | 0.633377 | 0.6231 |
| **26** | **0.05** | **22** | **26** | **0.05** | **0.91684** | **0.9321** |
| 27 | 0.05 | 12 | 46 | 0 | 0.415159 | 0.4226 |
| **28** | **0.1** | **22** | **36** | **0.05** | **0.760103** | **0.7612** |
| 29 | 0.05 | 12 | 46 | 0.1 | 0.416165 | 0.4237 |
