# Supplementary material for: Pharmacological activities of Artemisia absinthium and control of hepatic cancer by expression regulation of TGFβ1 and MYC genes
Source: PLoS One. 2023 Apr 13;18(4):e0284244. doi: 10.1371/journal.pone.0284244 (PMC10101520; doi:10.1371/journal.pone.0284244)
Supplement: S18 Table — (DOCX) [file pone.0284244.s030.docx]

Table S18:

| Runs | Klebsiella | Acinetobacter | Gram -ve bacilli | S. aureus | Antimicrobial activity | |
| --- | --- | --- | --- | --- | --- | --- |
|  |  |  |  |  | Actual | Predicted |
| **1** | **22** | **42** | **26** | **0.1** | **1.065483** | **1.08** |
| **2** | **12** | **42** | **36** | **0** | **1.066667** | **1.06** |
| **3** | **22** | **42** | **26** | **0** | **1.066667** | **1.08** |
| 4 | 22 | 42 | 16 | 0.05 | 0.949407 | 0.9541 |
| 5 | 2 | 42 | 26 | 0 | 0.8 | 0.8064 |
| **6** | **12** | **32** | **26** | **0.1** | **1.084165** | **1.07** |
| 7 | 2 | 32 | 26 | 0.05 | 0.932556 | 0.9172 |
| **8** | **12** | **32** | **36** | **0.05** | **1.099313** | **1.14** |
| 9 | 12 | 42 | 26 | 0.05 | 0.949407 | 0.9494 |
| 10 | 12 | 32 | 16 | 0.05 | 0.932556 | 0.9381 |
| **11** | **12** | **42** | **36** | **0.1** | **1.065483** | **1.05** |
| 12 | 22 | 52 | 26 | 0.05 | 0.95952 | 0.966 |
| **13** | **12** | **32** | **26** | **0** | **1.085714** | **1.07** |
| 14 | 12 | 52 | 26 | 0 | 0.844444 | 0.8506 |
| 15 | 12 | 42 | 16 | 0.1 | 0.798859 | 0.8011 |
| 16 | 2 | 42 | 16 | 0.05 | 0.5995 | 0.6142 |
| 17 | 12 | 52 | 16 | 0.05 | 0.699563 | 0.6702 |
| 18 | 12 | 42 | 16 | 0 | 0.8 | 0.8022 |
| 19 | 12 | 52 | 36 | 0.05 | 0.95952 | 0.9701 |
| 20 | 2 | 52 | 26 | 0.05 | 0.699563 | 0.6994 |
| 21 | 2 | 42 | 26 | 0.1 | 0.798859 | 0.8052 |
| 22 | 12 | 42 | 26 | 0.05 | 0.949407 | 0.9494 |
| 23 | 12 | 42 | 26 | 0.05 | 0.949407 | 0.9494 |
| **24** | **22** | **32** | **26** | **0.05** | **1.19925** | **1.19** |
| 25 | 12 | 42 | 26 | 0.05 | 0.949407 | 0.9494 |
| 26 | 12 | 42 | 26 | 0.05 | 0.949407 | 0.9494 |
| 27 | 2 | 42 | 36 | 0.05 | 0.949407 | 0.9374 |
| 28 | 12 | 52 | 26 | 0.1 | 0.843507 | 0.8498 |
| **29** | **22** | **42** | **36** | **0.05** | **1.15942** | **1.14** |
