# Supplementary material for: Pharmacological activities of Artemisia absinthium and control of hepatic cancer by expression regulation of TGFβ1 and MYC genes
Source: PLoS One. 2023 Apr 13;18(4):e0284244. doi: 10.1371/journal.pone.0284244 (PMC10101520; doi:10.1371/journal.pone.0284244)
Supplement: S20 Table — (DOCX) [file pone.0284244.s032.docx]

Table S20:

| **Source** | **df** | **Sum of Squares** | **Mean Square** | **F–value** | **p–value** |
| --- | --- | --- | --- | --- | --- |
| **Model** | 9 | 0.1583 | 0.0176 | 23.17 | 0.0002 |
| A–Leaf | 1 | 0.0013 | 0.0013 | 1.65 | 0.2402 |
| B–Stem | 1 | 0.0800 | 0.0800 | 105.41 | < 0.0001 |
| C–Flower | 1 | 0.0253 | 0.0253 | 33.35 | 0.0007 |
| AB | 1 | 0.0025 | 0.0025 | 3.29 | 0.1124 |
| AC | 1 | 0.0000 | 0.0000 | 0.0000 | 1.0000 |
| BC | 1 | 0.0056 | 0.0056 | 7.41 | 0.0297 |
| A² | 1 | 0.0007 | 0.0007 | 0.8669 | 0.3828 |
| B² | 1 | 0.0421 | 0.0421 | 55.48 | 0.0001 |
| C² | 1 | 0.0007 | 0.0007 | 0.8669 | 0.3828 |
| **Residual** | 7 | 0.0053 | 0.0008 |  |  |
| Lack of Fit | 3 | 0.0053 | 0.0018 |  |  |
| Pure Error | 4 | 0.0000 | 0.0000 |  |  |
| **Cor Total** | 16 | 0.1636 |  |  |  |

R^2^ = 0.97
