# Supplementary material for: Pharmacological activities of Artemisia absinthium and control of hepatic cancer by expression regulation of TGFβ1 and MYC genes
Source: PLoS One. 2023 Apr 13;18(4):e0284244. doi: 10.1371/journal.pone.0284244 (PMC10101520; doi:10.1371/journal.pone.0284244)
Supplement: S21 Table — (DOCX) [file pone.0284244.s033.docx]

Table S21:

| **Source** | **df** | **Sum of Squares** | **Mean Square** | **F–value** | **p–value** |
| --- | --- | --- | --- | --- | --- |
| **Model** | 9 | 0.0669 | 0.0074 | 20.82 | 0.0003 |
| A–Leaf | 1 | 0.0612 | 0.0612 | 171.50 | < 0.0001 |
| B–Stem | 1 | 0.0013 | 0.0013 | 3.50 | 0.1036 |
| C–Flower | 1 | 0.0000 | 0.0000 | 0.0000 | 1.0000 |
| AB | 1 | 0.0025 | 0.0025 | 7.00 | 0.0331 |
| AC | 1 | 0.0000 | 0.0000 | 0.0000 | 1.0000 |
| BC | 1 | 0.0000 | 0.0000 | 0.0000 | 1.0000 |
| A² | 1 | 0.0007 | 0.0007 | 1.84 | 0.2168 |
| B² | 1 | 0.0007 | 0.0007 | 1.84 | 0.2168 |
| C² | 1 | 0.0007 | 0.0007 | 1.84 | 0.2168 |
| **Residual** | 7 | 0.0025 | 0.0004 |  |  |
| Lack of Fit | 3 | 0.0025 | 0.0008 |  |  |
| Pure Error | 4 | 0.0000 | 0.0000 |  |  |
| **Cor Total** | 16 | 0.0694 |  |  |  |

R^2^ = 0.96
