# Supplementary material for: Pharmacological activities of Artemisia absinthium and control of hepatic cancer by expression regulation of TGFβ1 and MYC genes
Source: PLoS One. 2023 Apr 13;18(4):e0284244. doi: 10.1371/journal.pone.0284244 (PMC10101520; doi:10.1371/journal.pone.0284244)
Supplement: S22 Table — (DOCX) [file pone.0284244.s034.docx]

Table S22:

|  | **A–Leaves** | **B–Stem** | **C–Flower** | **Methanol** | | **Ethanol** | | **Acetone** | |
| --- | --- | --- | --- | --- | --- | --- | --- | --- | --- |
|  |  |  |  | **Experimental value** | **Predicted Value** | **Experimental value** | **Predicted Value** | **Experimental value** | **Predicted Value** |
| 1 | 1 | 1.1 | 1.1 | 1.1 | 1.09 | 1.1 | 1.09 | 1.1 | 1.09 |
| 2 | 1.05 | 1.1 | 1.2 | 1.1 | 1.1 | 1.1 | 1.1 | 1.1 | 1.1 |
| 3 | 1.05 | 1.15 | 1.1 | 1.1 | 1.11 | 1.1 | 1.11 | 1.1 | 1.11 |
| 4 | 1 | 1.15 | 1.2 | 1.15 | 1.16 | 1.15 | 1.16 | 1.15 | 1.16 |
| 5 | 1.05 | 1.1 | 1.2 | 1.1 | 1.1 | 1.1 | 1.1 | 1.1 | 1.1 |
| 6 | 1 | 1.05 | 1.2 | 1.05 | 1.06 | 1.05 | 1.06 | 1.05 | 1.06 |
| 7 | 1.05 | 1.1 | 1.2 | 1.1 | 1.1 | 1.1 | 1.1 | 1.1 | 1.1 |
| 8 | 1.05 | 1.05 | 1.3 | 1.05 | 1.04 | 1.05 | 1.04 | 1.05 | 1.04 |
| 9 | 1.05 | 1.1 | 1.2 | 1.1 | 1.1 | 1.1 | 1.1 | 1.1 | 1.1 |
| 10 | 1.1 | 1.05 | 1.2 | 1.1 | 1.09 | 1.1 | 1.09 | 1.1 | 1.09 |
| 11 | 1.05 | 1.1 | 1.2 | 1.1 | 1.1 | 1.1 | 1.1 | 1.1 | 1.1 |
| 12 | 1.1 | 1.1 | 1.3 | 1.1 | 1.11 | 1.1 | 1.11 | 1.1 | 1.11 |
| 13 | 1.05 | 1.15 | 1.3 | 1.15 | 1.14 | 1.15 | 1.14 | 1.15 | 1.14 |
| 14 | 1.1 | 1.1 | 1.1 | 1.1 | 1.1 | 1.1 | 1.1 | 1.1 | 1.1 |
| 15 | 1.1 | 1.15 | 1.2 | 1.15 | 1.14 | 1.15 | 1.14 | 1.15 | 1.14 |
| 16 | 1.05 | 1.05 | 1.1 | 1.05 | 1.06 | 1.05 | 1.06 | 1.05 | 1.06 |
| 17 | 1 | 1.1 | 1.3 | 1.1 | 1.1 | 1.1 | 1.1 | 1.1 | 1.1 |
